# Assessing tobacco-related ischemic stroke in Pakistan (1990–2019): Insights from the Global Burden of Disease Study

**DOI:** 10.18332/tid/185566

**Published:** 2024-03-28

**Authors:** Shuai Hou, Meng Pang, Yifeng Zhang, Yulei Xia, Yanqiang Wang, Gang Wang

**Affiliations:** 1 Emergency Department, Affiliated Hospital of Shandong Second Medical University, Weifang, People’s Republic of China; 2 School of Clinical Medicine, Shandong Second Medical University, Weifang, People’s Republic of China; 3 Department II of Neurology, Affiliated Hospital of Shandong Second Medical University, Weifang, People’s Republic of China

**Keywords:** ischemic stroke, active smoking, secondhand smoke, disease burden, Pakistan

## Abstract

**INTRODUCTION:**

This study analyzes the impact of active smoking and secondhand smoke on the ischemic stroke burden of Pakistan, 1990–2019.

**METHODS:**

We used data from the Global Burden of Disease (GBD) database to conduct a comprehensive evaluation of ischemic stroke-related indicators in Pakistan, including the number of deaths, mortality rates, disability-adjusted life years (DALYs), DALY rates, and the estimated annual percentage change (EAPC). Joinpoint analysis was applied to assess sex-specific temporal trends in the burden of active smoking and secondhand smoke in Pakistan and regions of Pakistan. These assessments incorporated the Socio-Demographic Index (SDI) and we have made comparative analyses of epidemiological differences between active smoking and secondhand smoke exposure.

**RESULTS:**

The burden of ischemic stroke related to tobacco use is presented in terms of the age-standardized mortality rate (ASMR) and the age-standardized disability-adjusted life year rate (ASDR) per 100000 population. The results (ASMR/ASDR) for Pakistan were 6.04/130.81, in the middle SDI region 7.69/176.54, and low-middle SDI region 5.64/124.22. Pakistan’s ASMR is higher than the global average of 5.85, while ASDR is lower than the global average of 140.23. From 1990 to 2019, a downward trend in both ASMR and ASDR was observed, indicating progress in controlling tobacco-related stroke burdens. Individuals aged ≥70 years experienced the highest rates of stroke (ASMR: 66.31; ASDR: 1091.20). Gender disparities were evident: men were more affected by active smoking (ASMR: 3.08; ASDR: 78.47) than women (ASMR: 0.79; ASDR: 20.76), while women faced a higher burden from secondhand smoke (ASMR: 0.66; ASDR: 16.33) compared to men (ASMR: 0.79; ASDR: 9.93). Regional differences within Pakistan show fluctuating death and DALY rates. Notably, an increasing trend in female ASDR due to secondhand smoke in the Khyber Pakhtunkhwa Region (annual percentage change, APC=0.17 from 2010 to 2019) calls for focused health interventions.

**CONCLUSIONS:**

The study finds ASMR for tobacco-related ischemic stroke in Pakistan exceeds global averages, with significant gender and age disparities in exposure to smoke, highlighting the need for targeted health interventions.

## INTRODUCTION

Ischemic stroke stands as a leading cause of death and disability globally, presenting a formidable public health concern in Pakistan^1,2^. Over the past three decades, the country has witnessed notable epidemiological shifts in this domain. To comprehensively understand these changes, encompassing mortality, incidence, and contributing risk factors, an in-depth investigation is essential. This is particularly vital in the context of tobacco consumption, including both active smoking and exposure to secondhand smoke, and its role in intensifying the burden of ischemic stroke^3,4^.

The detrimental role of tobacco use in fostering various chronic conditions, notably cardiovascular and cerebrovascular diseases, is well-established^5,6^. Nonetheless, the specific impact of tobacco on ischemic stroke is multifaceted, influenced by demographic shifts, public health policies, and societal attitudes toward smoking^7,8^. In Pakistan, with its evolving patterns of tobacco consumption, delineating tobacco’s specific contribution to ischemic stroke is imperative for designing effective public health strategies.

In the face of a globally evolving ischemic stroke burden, accentuated by factors like environmental pollution and tobacco use^9,10^, Pakistan’s significant rate of tobacco consumption presents a crucial case for investigation^11^. The insights gained from this study not only augment the existing understanding of ischemic stroke epidemiology in Pakistan but also hold critical implications for public health initiatives and policy formulation aimed at reducing the tobacco-induced stroke burden.

This study seeks to intricately examine the relationship between tobacco use and ischemic stroke in Pakistan and in the regions of Pakistan, from 1990 to 2019. Utilizing data from the Global Burden of Disease (GBD) study, this research investigates mortality rates, disability-adjusted life years (DALYs), DALY rates, and the estimated annual percentage change (EAPC), providing a comprehensive view of related trends and patterns. By contrasting the epidemiological differences between active smoking and secondhand smoke exposure, the study offers unique insights into tobacco’s impact on ischemic stroke across various age groups and gender.

## METHODS

### Research design and data acquisition

Our study is a secondary dataset analysis of GBD data. In this study, data pertaining to the burden of ischemic stroke disease linked to tobacco usage were acquired using the Global Health Data Exchange query tool developed by the Global Burden of Disease (GBD) Collaborators^12^. For smokers, our reference case definition was current daily or occasional use of any smoking tobacco product and previous use of any smoking tobacco product. We include all smoking products like cigarettes, pipes, cigars, shisha, bidis, kretek, and other local smoking products. We did not include smokeless tobacco, e-cigarettes (also known as e-cigarettes), vaping products, or heated tobacco products^13,14^. This data set encompasses standardized disease definitions, numbers of deaths, mortality rates, and disability-adjusted life years (DALYs) and their respective rates. We utilized the 2019 GBD study, which conducted an extensive evaluation of 369 diseases and injuries across 204 countries and territories from 1990 to 2019, encompassing incidence, mortality rates, DALYs, and corresponding uncertainty intervals^12,15^. We first used GBD (Global Burden of Disease) data to screen the ranking of ischemic stroke attributable to active smoking and secondhand smoke among all diseases (selecting all level 4 causes) caused by active and secondhand smoking. The rankings were determined based on the values of ASMR (age-standardized mortality rate) and ASDR (age-standardized disability-adjusted life year rate). Then, this study analyzed the age distribution of ischemic stroke patients affected by tobacco use, categorized into the age groups: 25–49, 50–69, and ≥70 years. The study employed linear regression to calculate the average annual percentage changes (EAPCs) in these groups^13^. Finally, and importantly, as our focal point of analysis, we examined the gender differences in Pakistan and its various regions through mortality and DALY indicators, including EAPC and joinpoint regression analysis.

### Socio-Demographic Index

The Socio-Demographic Index (SDI) serves as a critical measure of a country or region’s developmental level based on variables such as fertility rate, education level, and per capita income^12^. The SDI, which ranges from 0 to 1, uses higher values to indicate greater socio-economic development. Research has linked SDI to disease incidence and mortality rates. For the purpose of this study, countries and regions were classified into five SDI categories: low, low-middle, middle, high-middle, and high^16^. Regarding the specific country groupings of SDI, the GBD Collaborators gave very detailed groupings^17^. This categorization facilitated the comparison of ischemic stroke burden between Pakistan and other global regions with varying levels of economic development.

### Statistical analysis

This study processed all data using R Studio software (version 4.3.1). Key metrics analyzed included the number of deaths, mortality rates, DALYs, DALY rates, and their 30-year change rates attributed to the burden of ischemic stroke disease from tobacco use. We determined the 95% confidence intervals (95% CIs) for EAPCs using a linear model^18^. A downward trend in a rate is indicated when both the EAPC and the upper limit of its 95% CI are negative; conversely, an upward trend is suggested when both the EAPC and the lower limit of its 95% CI are positive. Furthermore, we employed the population attributable fraction (PAF) to estimate the proportion of events due to specific risk factors, like tobacco use^19^. This estimation helps quantify the preventable disease burden if such risk factors are eliminated, thereby assessing their impact on public health. Our analysis particularly focused on comparing age-standardized mortality and DALY rates for Pakistan, globally, and across regions of Pakistan with different economic development levels, estimating their temporal trends through calculating EAPC analysis. To analyze temporal trends in age-standardized mortality rate (ASMR) and age-standardized disability-adjusted life year rate (ASDR) from 1990 to 2019, we utilized the estimated annual percentage change (EAPC) based on age-standardized rates (ASR) for each year. The EAPC assumes a linear relationship between ASRs and time, modeled as y = α + βx + ε, where y represents log_10_ (ASR), x the calendar year, and β the regression coefficient. The EAPC is calculated using the formula EAPC = 100×(10^β^ -1). In contrast to the 95% uncertainty interval (UI) used for other estimates, the EAPC is accompanied by a 95% confidence interval (95% CI). An ASR is considered to exhibit an upward trend if the EAPC and the lower bound of its 95% CI are both greater than zero, and vice versa^20^.

To address the limitations of the estimated annual percentage change (EAPC) in capturing localized variations, we calculated the standard errors for Global Burden of Disease (GBD) estimates by dividing the width of the 95% uncertainty interval (UI) by 3.92^21^. By employing the Delta method to calculate these standard errors, we constructed 95% confidence intervals (CIs) and conducted trend analyses^21^. The joinpoint regression model was employed to examine the temporal trends in tobacco use, encompassing both active and passive smoking associated with ischemic stroke. Data analysis and visualization were performed using the *Joinpoint* software, adopting a log-linear model with a significance level set at α=0.05^13,22^. The default approach for modeling in joinpoint regression is the Grid Search Method (GSM), supplemented by the Monte Carlo Permutation method for model selection. The joinpoint model yielded the annual percentage change (APC) and the average annual percentage change (AAPC) over the study period, complete with their 95% CIs. Trends were classified as increasing (indicating a worsening situation) or decreasing (indicating improvement) if the APC or AAPC significantly deviated from zero^21^. Conversely, a lack of significant difference denoted a stable or unchanged trend. The level of statistical significance for all analyses conducted was set at p<0.05, and tests were two-tailed. This approach underscores the meticulous evaluation of trend directions and reinforces the rigor of our analysis.

## RESULTS

### Ranking of diseases attributable to active smoking and secondhand smoke

In the GBD database, by selecting all level 4 causes, the indicators attributed to active smoking correspond to 28 diseases. The top five diseases ranked by ASMR from highest to lowest are intracerebral hemorrhage, drug-susceptible tuberculosis, ischemic stroke, diabetes mellitus type 2, and subarachnoid hemorrhage, with ischemic stroke ranking third. The top five diseases ranked by ASDR from highest to lowest are drug-susceptible tuberculosis, intracerebral hemorrhage, diabetes mellitus type 2, ischemic stroke, and subarachnoid hemorrhage, with ischemic stroke ranking fourth. However, the indicators attributed to secondhand smoke only correspond to 4 diseases, with the rankings by both ASMR and ASDR being diabetes mellitus type 2, intracerebral hemorrhage, ischemic stroke, and subarachnoid hemorrhage, with ischemic stroke ranking third (Supplementary file Tables 1–3).

### Burden of ischemic stroke disease attributable to tobacco use

**Figure 1:**
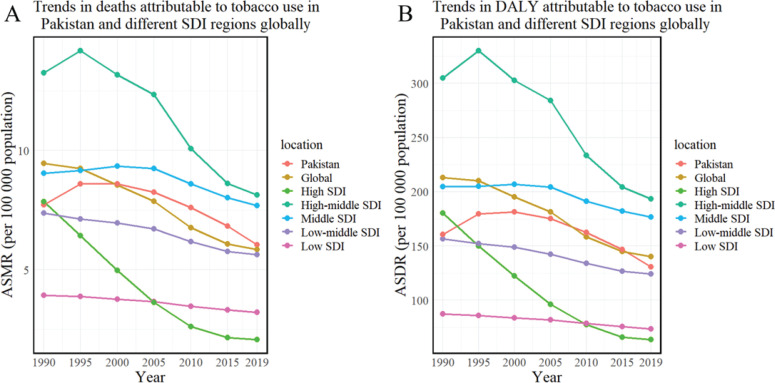
Age-standardized mortality and DALY rates of ischemic stroke attributed to tobacco in Pakistan, globally, and across SDI regions (1990–2019): A) Trends in deaths attributable to tobacco use in Pakistan and different SDI regions globally; B) Trends in DALY rates attributable to tobacco use in Pakistan and different SDI regions globally

Between 1990 and 2019, the age-standardized mortality rate (ASMR) and the age-standardized disability-adjusted life year (DALY) rate for ischemic stroke attributable to tobacco use in Pakistan, as well as globally and across different Socio-Demographic Index (SDI) regions of Pakistan, exhibited a decreasing trend. However, the decline in Pakistan was relatively modest within the low SDI category, with the EAPC being -0.95 (95% CI: -1.22 – -0.68) for ASMR and -0.81 (95% CI: -1.08 – -0.54) for ASDR. By 2019, the ASMR and ASDR per 100000 people in Pakistan were 6.04 and 130.81, respectively. This placed Pakistan’s ASMR above the global average (5.85) and its ASDR below the global average (140.23). Comparatively, high SDI regions reported 2.08 for ASMR and 63.88 for ASDR; high-middle SDI regions, 8.14 and 193.41; middle SDI regions, 7.69 and 176.54; low-middle SDI regions, 5.64 and 124.22; and low SDI regions, 3.21 and 73.55. These figures suggest that Pakistan’s burden of tobacco-related ischemic stroke disease is between the middle and low-middle SDI levels. These findings are detailed in Figure 1, Table 1, and Supplementary file Figure 1.

**Table 1:** Evolution of disease burden for ischemic stroke attributed to tobacco, smoking, and secondhand smoke in Pakistan, globally, and across different SDI regions (1990–2019)

*Risk factor*	ASMR*			ASDR*		
	*1990*	*2019*	*EAPC (95% CI)*	*1990*	*2019*	*EAPC (95% CI)*
**Tobacco**						
Pakistan	7.72	6.04	-0.95 (-1.22 – -0.68)	160.78	130.81	-0.81 (-1.08 – -0.54)
Global	9.46	5.85	-1.91 (-2.03 – -1.79)	213.08	140.23	-1.68 (-1.78 – -1.57)
High SDI	7.86	2.08	-5.09 (-5.31 – -4.86)	180.27	63.88	-3.86 (-4.02 – -3.7)
High-middle SDI	13.24	8.14	-2.11 (-2.37 – -1.85)	304.83	193.41	-2.01 (-2.25 – -1.77)
Middle SDI	9.05	7.69	-0.63 (-0.77 – -0.49)	204.71	176.54	-0.57 (-0.67 – -0.47)
Low-middle SDI	7.37	5.64	-1.02 (-1.11 – -0.92)	156.62	124.22	-0.89 (-0.95 – -0.82)
Low SDI	3.93	3.21	-0.74 (-0.8 – -0.68)	87.31	73.55	-0.62 (-0.66 – -0.57)
**Smoking**						
Pakistan	6.48	4.78	-1.07 (-1.36 – -0.79)	137.09	106.34	-0.91 (-1.19 – -0.62)
Global	8.05	4.89	-1.98 (-2.1 – -1.85)	185.31	120.37	-1.72 (-1.83 – -1.61)
High SDI	7.24	1.87	-5.16 (-5.37 – -4.94)	168.29	58.79	-3.89 (-4.05 – -3.74)
High-middle SDI	11.03	6.89	-2.06 (-2.33 – -1.79)	263.11	169.12	-1.97 (-2.21 – -1.73)
Middle SDI	7.27	6.29	-0.59 (-0.74 – -0.43)	169.28	148.32	-0.53 (-0.64 – -0.41)
Low-middle SDI	6.11	4.54	-1.11 (-1.21 – -1.02)	132.58	102.65	-0.96 (-1.04 – -0.89)
Low SDI	3.18	2.54	-0.79 (-0.85 – -0.73)	72.40	59.72	-0.67 (-0.71 – -0.62)
**Secondhand smoke**						
Pakistan	1.43	1.39	-0.49 (-0.73 – -0.25)	28.08	27.67	-0.46 (-0.7 – -0.22)
Global	1.63	1.10	-1.59 (-1.7 – -1.49)	33.50	23.47	-1.46 (-1.55 – -1.36)
High SDI	0.76	0.24	-4.56 (-4.82 – -4.31)	16.34	6.37	-3.68 (-3.92 – -3.44)
High-middle SDI	2.55	1.45	-2.35 (-2.58 – -2.11)	50.55	29.63	-2.26 (-2.49 – -2.03)
Middle SDI	2.02	1.60	-0.83 (-0.9 – -0.75)	41.37	32.91	-0.82 (-0.87 – -0.76)
Low-middle SDI	1.44	1.23	-0.68 (-0.77 – -0.59)	28.18	24.59	-0.58 (-0.64 – -0.52)
Low SDI	0.83	0.73	-0.54 (-0.6 – -0.48)	16.79	15.45	-0.4 (-0.45 – -0.35)

* ASMR: age-standardized mortality rate per 100000 population. ASDR: age-standardized disability-adjusted life year rate per 100000 population. EAPC: estimated annual percentage change.

### Trends in tobacco-attributable ischemic stroke deaths

In 2019, approximately 13.98% of ischemic stroke deaths in Pakistan were attributed to tobacco use, totaling 5457 deaths at a rate of 2.44 per 100000. Gender disparities were evident, with males accounting for a higher number of tobacco-attributable deaths (3913 cases; 3.41) compared to females (1544 cases; 1.41). A clear age-related increase was noted in both the number and rate of tobacco-related deaths, peaking in individuals aged ≥70 years.

**Figure 2:**
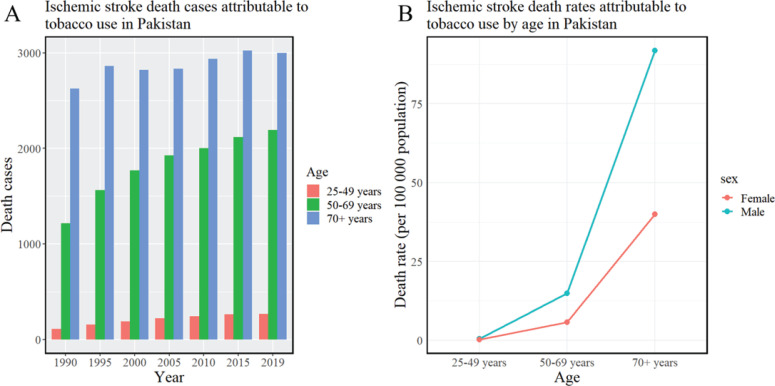
Trends in ischemic stroke mortality attributed to tobacco in Pakistan (1990–2019): A) Ischemic stroke death cases attributable to tobacco use; B) Ischemic stroke death rates attributable to tobacco use by age

From 1990 to 2019, the total number of tobacco-related ischemic stroke deaths increased by 38.13%, while the overall mortality rate decreased by 30.44%. The increase in male deaths was 28.77%, accompanied by a 33.71% decrease in the male mortality rate. In contrast, the number of female deaths rose by 69.32%, significantly outpacing the male rate, although the mortality rate among females only decreased by 16.73%. These data are shown in Figure 2, Table 2, and Supplementary file Figure 2.

**Table 2:** Trends in ischemic stroke mortality attributed to tobacco, smoking, and secondhand smoke by age, year, and gender, Pakistan (1990–2019)

*Risk factors*	*PAF (%)*			*Death cases*			Death rate*		
	*All*	*Male*	*Female*	*All*	*Male*	*Female*	*All*	*Male*	*Female*
**Tobacco**									
**Age 15–49 years**									
1990	26.47	37.39	17.67	111	70	41	0.39	0.48	0.3
2000	25.52	35.44	16.68	190	124	66	0.51	0.64	0.36
2010	24.21	33.34	15.07	242	167	75	0.48	0.64	0.3
2019	20.55	28.56	13.42	269	176	93	0.41	0.53	0.28
Rate of change, %	-22.36	-23.62	-24.05	142.25	149.76	129.25	3.39	10.86	-6.10
**Age 50–69 years**									
1990	27.18	34.93	16.17	1216	921	294	12.7	17.69	6.75
2000	25.91	33.44	15.39	1771	1335	436	15.18	21.42	8.02
2010	24.35	31.63	14.29	1999	1512	487	13.1	18.79	6.76
2019	20.77	28.02	11.97	2192	1626	565	10.63	14.94	5.8
Rate of change, %	-23.58	-19.78	-25.97	80.3	76.52	92.14	-16.36	-15.53	-13.99
**Age ≥70 years**									
1990	15	21.4	7.29	2624	2047	577	89.01	122.64	45.14
2000	14.29	20.53	7.5	2820	2111	709	95.9	132.62	52.56
2010	13.67	20.14	7.58	2935	2096	839	84.21	117.05	49.5
2019	11.15	17.42	6.01	2997	2111	886	66.31	91.81	39.91
Rate of change, %	-25.67	-18.6	-17.56	14.20	3.12	53.48	-25.51	-25.14	-11.6
**All ages**									
1990	17.5	24.38	9.04	3951	3039	912	3.5	5.14	1.7
2000	17.38	24.27	9.45	4780	3570	1210	3.36	4.84	1.77
2010	16.73	23.88	9.27	5176	3775	1401	2.83	4	1.58
2019	13.98	20.95	7.59	5457	3913	1544	2.44	3.41	1.41
Rate of change, %	-20.11	-14.07	-16.04	38.13	28.77	69.32	-30.44	-33.71	-16.73
**Smoking**									
**Age 15–49 years**									
1990	20.14	34.51	8.55	85	65	20	0.3	0.44	0.15
2000	19.34	32.46	7.64	144	114	30	0.38	0.59	0.17
2010	18.52	30.24	6.8	185	151	34	0.36	0.58	0.14
2019	14.73	25.23	5.38	193	155	37	0.29	0.47	0.11
Rate of change, %	-26.86	-26.89	-37.08	127.83	139.05	90.73	-2.77	6.10	-21.87
**Age 50–69 years**									
1990	23.54	32.63	10.63	1054	860	193	11.01	16.52	4.44
2000	22.28	31.04	10.04	1524	1239	284	13.06	19.88	5.23
2010	20.99	29.25	9.57	1724	1398	327	11.3	17.37	4.53
2019	17.37	25.56	7.44	1835	1484	351	8.9	13.63	3.61
Rate of change, %	-26.21	-21.67	-30.01	74.11	72.42	81.62	-19.23	-17.5	-18.7
**Age ≥70 years**									
1990	12.61	19.66	4.12	2206	1880	326	74.82	112.63	25.49
2000	11.81	18.76	4.25	2330	1928	402	79.25	121.17	29.77
2010	11.35	18.46	4.67	2438	1921	516	69.94	107.27	30.48
2019	8.84	15.71	3.2	2376	1903	472	52.56	82.78	21.26
rate of change, %	-29.9	-20.09	-22.33	7.68	1.24	44.82	-29.76	-26.50	-16.59
**All ages**									
1990	14.81	22.51	5.34	3344	2805	539	2.96	4.75	1
2000	14.53	22.31	5.59	3998	3282	716	2.81	4.44	1.05
2010	14.05	21.95	5.8	4347	3470	877	2.38	3.68	0.99
2019	11.27	18.97	4.23	4403	3542	861	1.97	3.08	0.79
Rate of change, %	-23.90	-15.73	-20.79	31.65	26.26	59.69	-33.70	-35.00	-21.46
**Secondhand smoke**									
**Age 50–69 years**									
1990	7.37	4.18	9.93	31	8	23	0.11	0.05	0.17
2000	7.15	4.22	9.75	53	15	38	0.14	0.08	0.21
2010	6.55	4.25	8.85	65	21	44	0.13	0.08	0.18
2019	6.51	4.3	8.47	85	26	59	0.13	0.08	0.18
Rate of change, %	-11.67	2.87	-14.70	177.14	236.15	156.88	18.28	49.20	5.22
**Age 50–69 years**									
1990	4.57	3.44	6.19	203	91	113	2.13	1.74	2.58
2000	4.51	3.49	5.94	308	140	168	2.64	2.24	3.09
2010	4.15	3.39	5.2	340	162	177	2.23	2.02	2.46
2019	4.03	3.32	4.88	424	193	231	2.05	1.77	2.37
Rate of change, %	-11.82	-3.49	-21.16	108.38	112.58	104.98	-3.33	1.72	-8.24
**Age ≥70 years**									
1990	2.72	2.21	3.33	476	211	264	16.13	12.66	20.66
2000	2.79	2.22	3.42	551	228	323	18.75	14.32	23.97
2010	2.61	2.1	3.08	559	218	340	16.03	12.18	20.1
2019	2.53	2.06	2.91	679	249	430	15.03	10.85	19.35
Rate of change, %	-6.99	-6.79	-12.61	42.84	18.11	62.60	-6.83	-14.26	-6.34
**All ages**									
1990	3.15	2.49	3.96	710	310	400	0.63	0.52	0.74
2000	3.32	2.6	4.14	912	382	530	0.64	0.52	0.77
2010	3.12	2.54	3.72	964	402	562	0.53	0.43	0.63
2019	3.05	2.51	3.53	1188	469	719	0.53	0.41	0.66
Rate of change, %	-3.17	0.80	-10.86	67.42	51.31	79.91	-15.69	-22.11	-11.51

PAF: population attributable fraction.

* Per 100000 population.

**Figure 3:**
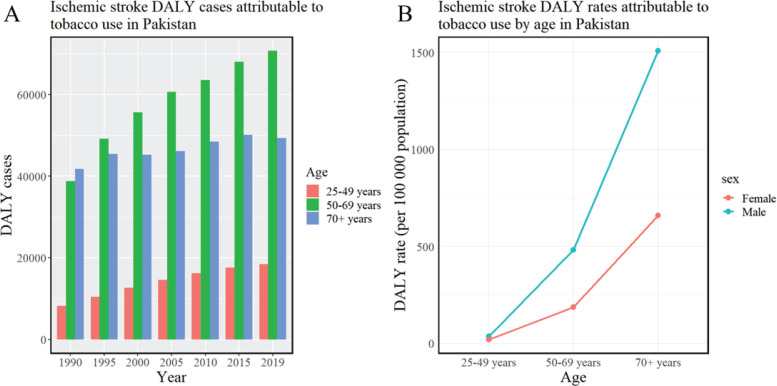
Trends in ischemic stroke DALYs attributed to tobacco in Pakistan (1990–2019): A) Ischemic stroke DALY cases attributable to tobacco use; B) Ischemic stroke DALY rates attributable to tobacco use by age

**Figure 4:**
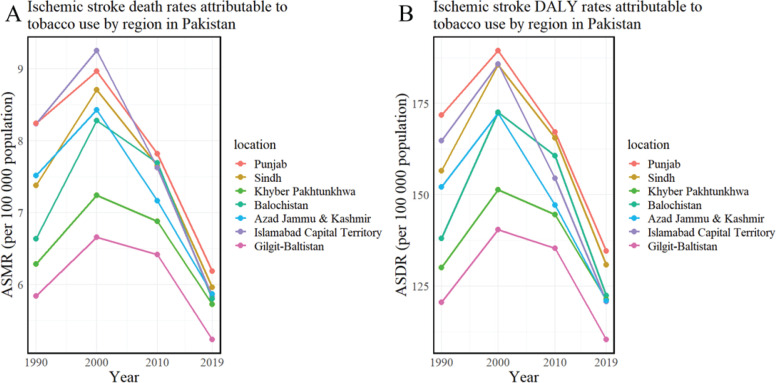
Age-standardized mortality and DALY rates of ischemic stroke attributed to tobacco in regions of Pakistan (1990–2019): A) Ischemic stroke death rates attributable to tobacco use; B) Ischemic stroke DALY rates attributable to tobacco use

Regionally within Pakistan, the highest tobacco-related ASMR per 100000 was observed in Punjab (6.19), with the lowest in Gilgit-Baltistan (5.24). The highest ASMR due to active smoking and secondhand smoke exposure was recorded in Sindh (4.93) and Khyber Pakhtunkhwa (1.61), respectively, while the lowest rates were seen in Gilgit-Baltistan (3.85) and Sindh (1.17). During the period from 1990 to 2019, there was a general decrease in the age-standardized mortality rate (ASMR) of ischemic stroke due to tobacco and active smoking in several regions of Pakistan, evidenced by negative estimated annual percentage changes (EAPCs). In contrast, in Khyber Pakhtunkhwa and Gilgit-Baltistan, the ASMR associated with exposure to secondhand smoke showed an increasing trend. The EAPC in Khyber Pakhtunkhwa was 0.28 (95% CI: 0.12–0.44), and in Gilgit-Baltistan 0.28 (95% CI: -0.01–0.57) (Figures 3 and 4, and Table 3).

**Table 3:** Regional trends in ischemic stroke disease burden attributed to tobacco, by regions of Pakistan (1990–2019)

*Risk factors*	ASMR*			ASDR*		
	*1990*	*2019*	*EAPC (95% CI)*	*1990*	*2019*	*EAPC (95% CI)*
**Tobacco**						
Pakistan	7.72	6.04	-0.95 (-1.22 – -0.68)	160.78	130.81	-0.81 (-1.08 – -0.54)
Punjab	8.24	6.19	-1.1 (-1.35 – -0.85)	171.84	134.62	-0.95 (-1.2 – -0.7)
Sindh	7.38	5.96	-0.87 (-1.2 – -0.53)	156.53	130.77	-0.74 (-1.07 – -0.41)
Khyber Pakhtunkhwa	6.28	5.73	-0.37 (-0.64 – -0.11)	130.08	121.34	-0.29 (-0.55 – -0.02)
Balochistan	6.64	5.8	-0.49 (-0.89 – -0.09)	138.06	122.33	-0.45 (-0.84 – -0.05)
Azad Jammu and Kashmir	7.52	5.87	-1.05 (-1.32 – -0.78)	152.13	121.02	-0.98 (-1.25 – -0.71)
Islamabad Capital Territory	8.24	5.83	-1.3 (-1.62 – -0.99)	164.74	120.73	-1.2 (-1.5 – -0.89)
Gilgit-Baltistan	5.84	5.24	-0.27 (-0.58–0.04)	120.51	110.41	-0.23 (-0.55–0.1)
**Smoking**						
Pakistan	6.48	4.78	-1.07 (-1.36 – -0.79)	137.09	106.34	-0.91 (-1.19 – -0.62)
Punjab	6.93	4.91	-1.21 (-1.48 – -0.95)	146.71	109.76	-1.04 (-1.3 – -0.78)
Sindh	6.35	4.93	-0.95 (-1.29 – -0.6)	136.8	110.54	-0.8 (-1.14 – -0.47)
Khyber Pakhtunkhwa	5.08	4.25	-0.59 (-0.9 – -0.28)	106.75	93.11	-0.44 (-0.74 – -0.13)
Balochistan	5.53	4.58	-0.61 (-1.05 – -0.17)	116.94	98.03	-0.57 (-1 – -0.13)
Azad Jammu and Kashmir	6.29	4.52	-1.27 (-1.56 – -0.99)	128.45	95.15	-1.16 (-1.45 – -0.87)
Islamabad Capital Territory	6.73	4.42	-1.5 (-1.85 – -1.16)	137.49	94.49	-1.36 (-1.7 – -1.03)
Gilgit-Baltistan	4.64	3.85	-0.47 (-0.79 – -0.14)	96.72	83.18	-0.38 (-0.73 – -0.03)
**Secondhand smoke**						
Pakistan	1.43	1.39	-0.49 (-0.73 – -0.25)	28.08	27.67	-0.46 (-0.7 – -0.22)
Punjab	1.52	1.41	-0.68 (-0.92 – -0.45)	29.8	28.11	-0.64 (-0.88 – -0.41)
Sindh	1.22	1.17	-0.58 (-0.9 – -0.25)	24.19	23.55	-0.5 (-0.82 – -0.19)
Khyber Pakhtunkhwa	1.36	1.61	0.28 (0.12–0.44)	26.86	31.23	0.21 (0.03–0.38)
Balochistan	1.27	1.35	-0.1 (-0.36–0.16)	24.8	27.21	0.0 (-0.27–0.26)
Azad Jammu and Kashmir	1.42	1.47	-0.28 (-0.52 – -0.04)	27.8	28.66	-0.32 (-0.56 – -0.08)
Islamabad Capital Territory	1.71	1.54	-0.67 (-0.92 – -0.42)	31.76	29.11	-0.62 (-0.86 – -0.37)
Gilgit-Baltistan	1.34	1.5	0.28 (-0.01–0.57)	26.83	29.75	0.22 (-0.05–0.5)

* ASMR: age-standardized mortality rate per 100000 population. ASDR: age-standardized disability-adjusted life year rate per 100000 population. EAPC: estimated annual percentage change.

### Trends in tobacco-attributable ischemic stroke burden

**Figure 5:**
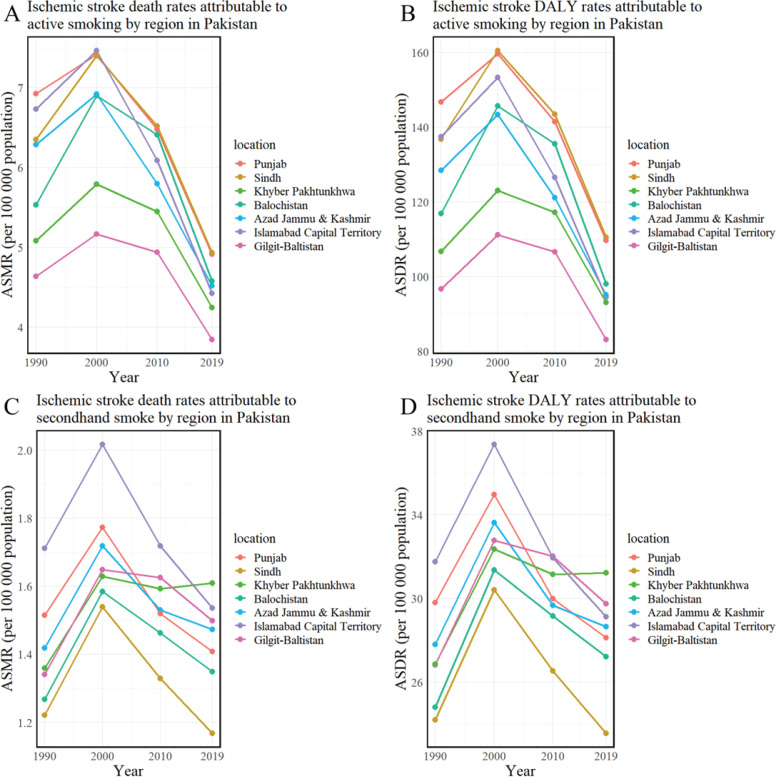
Age-standardized mortality and DALY rates of ischemic stroke attributed to active smoking and secondhand smoke in regions of Pakistan (1990–2019): A) Ischemic stroke death rates attributable to active smoking; B) Ischemic stroke DALY rates attributable to active smoking; C) Ischemic stroke death rates attributable to secondhand smoking; D) Ischemic stroke DALY rates attributable to secondhand smoking

By 2019, the disability-adjusted life years (DALYs) and DALY rate for ischemic stroke due to tobacco in Pakistan were recorded at 138431 person-years and 61.78 per 100000 people, respectively. A notable gender disparity was observed, with males exhibiting significantly higher DALYs (98905 person-years; 86.12) compared to females (39525 person-years; 36.19). As age increased, both DALYs and DALY rates demonstrated an upward trend, predominantly concentrating in those aged 50–69 years, while the highest DALY rate was noted in individuals aged ≥70 years (Figure 5 and Table 4).

**Table 4:** Evolution of disease burden from ischemic stroke attributed to tobacco, smoking, and secondhand smoke, by age, year, and gender, Pakistan (1990–2019)

*Risk factors*	*DALY cases*			DALY rate*		
	*All*	*Male*	*Female*	*All*	*Male*	*Female*
**Tobacco**						
**Age 15–49 years**						
1990	8176	5131	3045	29.06	34.99	22.61
2000	12649	8064	4585	33.67	41.45	25.31
2010	16193	10758	5434	31.84	41.56	21.77
2019	18416	11732	6684	27.93	35.51	20.32
Rate of change, %	125.24	128.66	119.48	-3.87	1.49	-10.10
**Age 50–69 years**						
1990	38805	29176	9628	405.58	560.26	220.83
2000	55602	41693	13909	476.64	668.81	256.08
2010	63593	47905	15688	416.72	595.39	217.46
2019	70692	52502	18191	342.78	482.42	186.75
Rate of change, %	82.17	79.95	88.92	-15.48	-13.89	-15.43
**Age ≥70 years**						
1990	41814	32241	9574	1418.25	1931.68	748.38
2000	45219	33525	11693	1537.98	2106.63	867
2010	48474	34334	14139	1390.87	1917.09	834.59
2019	49323	34672	14651	1091.2	1508.05	659.68
Rate of change, %	17.96	7.54	53.03	-23.06	-21.93	-11.85
**All ages**						
1990	88795	66548	22247	78.69	112.56	41.42
2000	113470	83282	30187	79.81	112.79	44.17
2010	128259	92997	35262	70.08	98.65	39.74
2019	138431	98905	39525	61.78	86.12	36.19
Rate of change, %	55.90	48.62	77.66	-21.49	-23.49	-12.62
**Smoking**						
**Age 15–49 years**						
1990	6227	4716	1511	22.13	32.16	11.22
2000	9495	7350	2145	25.27	37.78	11.85
2010	12237	9716	2521	24.06	37.54	10.1
2019	13139	10340	2799	19.93	31.29	8.51
Rate of change, %	111.00	119.27	85.21	-9.95	-2.68	-24.13
**Age 50–69 years**						
1990	33818	27404	6414	353.46	526.22	147.11
2000	48064	38912	9152	412.02	624.21	168.49
2010	55145	44562	10582	361.36	553.85	146.68
2019	59691	48272	11419	289.43	443.55	117.23
Rate of change, %	76.51	76.15	78.02	-18.11	-15.71	-20.31
**Age ≥70 years**						
1990	35676	29828	5848	1210.06	1787.15	457.13
2000	38057	30881	7176	1294.39	1940.44	532.08
2010	41017	31707	9310	1176.91	1770.42	549.5
2019	39963	31510	8454	884.13	1370.51	380.63
Rate of change, %	12.02	5.64	44.56	-26.93	-23.31	-16.73
**All ages**						
1990	75721	61948	13773	67.11	104.78	25.64
2000	95617	77143	18474	67.25	104.48	27.03
2010	108399	85986	22413	59.23	91.21	25.26
2019	112792	90121	22671	50.34	78.47	20.76
Rate of change, %	48.96	45.48	64.60	-24.99	-25.11	-19.04
**Secondhand smoke**						
**Age 15–49 years**						
1990	2226	572	1654	7.91	3.9	12.28
2000	3580	968	2611	9.53	4.98	14.42
2010	4468	1376	3093	8.79	5.31	12.39
2019	5822	1748	4075	8.83	5.29	12.39
Rate of change, %	161.54	205.53	146.33	11.62	35.61	0.90
**Age 50–69 years**						
1990	6263	2680	3583	65.46	51.46	82.17
2000	9376	4109	5268	80.38	65.91	96.98
2010	10438	4821	5616	68.4	59.92	77.85
2019	13081	5793	7288	63.43	53.23	74.82
Rate of change, %	108.87	116.16	103.41	-3.10	3.44	-8.95
**Age ≥70 years**						
1990	7078	3121	3957	240.06	186.97	309.33
2000	8183	3383	4800	278.31	212.57	355.87
2010	8496	3342	5154	243.79	186.63	304.22
2019	10338	3860	6477	228.7	167.9	291.65
Rate of change, %	46.06	23.70	63.69	-4.73	-10.20	-5.72
**All ages**						
1990	15567	6372	9194	13.8	10.78	17.12
2000	21139	8460	12679	14.87	11.46	18.55
2010	23402	9539	13863	12.79	10.12	15.62
2019	29241	11401	17840	13.05	9.93	16.33
Rate of change, %	87.84	78.91	94.04	-5.40	-7.90	-4.57

* Per 100000 population.

From 1990 to 2019, the overall DALYs increased by 55.90%, whereas the DALY rate decreased by 21.49%. The gender-specific analysis revealed a 48.62% increase in DALYs for males, with a 23.49% reduction in the rate. Conversely, female DALYs increased by 77.66%, which is substantially higher than male DALYs, but the rate decrease was only 12.62% (Table 4).

Regionally, Punjab reported the highest tobacco-related age-standardized DALY rate (ASDR) at 134.62 per 100000 people, with the lowest in Gilgit-Baltistan at 110.41. The highest ASDR for active smoking and secondhand smoke exposure was observed in Sindh (110.54) and Khyber Pakhtunkhwa (31.23), respectively, while Gilgit-Baltistan (83.18) and Sindh (23.55) had the lowest. From 1990 to 2019, the age-standardized death rate (ASDR) for ischemic stroke, attributed to active smoking and tobacco use, showed a declining trend in several Pakistani regions, as evidenced by estimated annual percentage changes (EAPCs) below zero. In contrast, the ASDR related to secondhand smoke exposure in Khyber Pakhtunkhwa and Gilgit-Baltistan was increasing. The EAPC for Khyber Pakhtunkhwa was 0.21 (95% CI: 0.03–0.38), while Gilgit-Baltistan’s EAPC was slightly higher at 0.22 (95% CI: -0.05–0.50) (Figures 3 and 4, and Table 3).

### Comparative analysis of active smoking and secondhand smoke attributable stroke burden

In 2019, marked gender differences were evident in the burden of ischemic stroke disease caused by both active smoking and secondhand smoke in Pakistan, as detailed in Tables 2 and 4 and Supplementary file Figures 2 and 3. Among male residents, 18.97% of ischemic stroke deaths were attributed to active smoking, compared to 4.23% in females. For strokes caused by secondhand smoke, the proportions were 2.51% for males and 3.53% for females.

Regarding burden indicators due to active smoking, males had higher figures than females. Specifically, male deaths numbered 3542 (mortality rate of 3.08 per 100000 people), while females accounted for 861 deaths with mortality rate of 0.79. The male DALYs were 90121 (DALY rate 78.47), and for females it was 22671 (20.76). Conversely, for indicators related to secondhand smoke, females showed higher figures than males. Male deaths totaled 469 and females 719, with mortality rates per 100000 people of 0.41 and 0.66, respectively. The male DALYs were 11401 (9.93), and for females were 17840 (16.33). The highest mortality rate, DALYs, and DALY rate for strokes attributed to both active smoking and secondhand smoke were in those aged ≥70 years, while the highest DALYs were noted in those aged 50–69 years.

From 1990 to 2019, an increase in the number of deaths and DALYs was observed, alongside a decrease in both the mortality rate and DALY rate. Notably, the most significant increases in deaths and DALYs due to secondhand smoke in females were 79.91% and 94.04%, respectively, with the smallest decreases in mortality rate and DALY rate were 11.51% and 4.57%, respectively. Among males, the smallest increase in deaths and DALYs attributed to active smoking was paired with the largest decrease in mortality rate and DALY rate, at 26.26%, 45.48%, 35.00%, and 25.11%, respectively.

From 1990 to 2019, the overall average annual percentage change (AAPC) for Pakistan shows that, except for the AAPC of DALYs attributable to secondhand smoke in males, which was not statistically significant with a 95% confidence interval spanning zero, all AAPC values were less than zero (Supplementary file Table 4). This indicates that from 1990 to 2019, the incidence and mortality rates of ischemic stroke attributable to tobacco use in Pakistan did not show a declining trend. In Supplementary file Figures 4 and 5, it can be observed that for active smoking, the annual percentage change (APC) was positive before it turned negative around 1997 for males and 2003 for females. This means that for active smoking, the age-standardized mortality rate (ASMR) and age-standardized disability-adjusted life year rates (ASDR) for males were on an increasing trend from 1990 to 1997, and then on a decreasing trend from 1997 to 2019; for females, these rates increased from 1990 to 2003 and then decreased from 2003 to 2019. Similarly, the male ASMR attributable to secondhand smoke was increasing before 1996 and then showed a decreasing trend from 1996 to 2019. Before 1999, the ASDR showed an increasing trend, and from 1999 to 2019, the ASDR showed a decreasing trend. The female ASMR and ASDR attributable to secondhand smoke both showed an increasing trend from 1990 to 1998 and then a decreasing trend from 1998 to 2019.

For the AAPC from 1990 to 2019 across regions in Pakistan, except for the AAPC of female ASMR attributable to active smoking, which was not statistically significant with a 95% confidence interval spanning zero, both ASMR and ASDR showed a decreasing trend (AAPC<0). This suggests that from 1990 to 2019, the incidence and mortality rates of ischemic stroke attributable to tobacco use in various regions of Pakistan showed a declining trend.

However, for ischemic stroke attributable to secondhand smoke, as shown in Supplementary file Table 5, among the statistically significant data, the ASDR for males in Azad Jammu and Kashmir showed an increasing trend (AAPC=0.1; 95% CI: 0.0–0.2); in Balochistan, the male ASDR showed an increasing trend (AAPC=0.3; 95% CI: 0.1–0.5), and for females, both ASMR (AAPC=0.4; 95% CI: 0.3–0.5) and ASDR (AAPC=0.4; 95% CI: 0.2–0.5) showed an increasing trend; in Gilgit-Baltistan, the male ASDR showed an increasing trend (AAPC=0.3; 95% CI: 0.2–0.5), and for females, both ASMR (AAPC=0.5; 95% CI: 0.2–0.8) and ASDR (AAPC=0.4; 95% CI: 0.1–0.6) showed an increasing trend. However, Supplementary file Figure 5 shows that the most recent trend lines up to 2019 for Azad Jammu and Kashmir, Balochistan, and Gilgit-Baltistan are all on a downward trend, indicating that despite the AAPC showing an upward trend, the recent years have seen a downward trend. In the Khyber Pakhtunkhwa region, the AAPC values for both male and female ASMR and ASDR were above zero. However, as shown in Supplementary file Figures 6 and 7, for mortality indicators, the Annual Percentage Change (APC) of the ASMR for both males and females in the Khyber Pakhtunkhwa region was not statistically significant, with a 95% Confidence Interval (CI) spanning zero. This indicates that the mortality rate in the region has been relatively stable in recent years. For DALY rates, recent data are statistically significant; the male ASDR has shown a downward trend (APC= -0.48 from 2009 to 2019), while the female ASDR has exhibited a noticeable upward trend in recent years (APC=0.17 from 2010 to 2019).

## DISCUSSION

This study’s exploration of the ischemic stroke burden attributable to tobacco use in Pakistan from 1990 to 2019 unveils significant insights into the changing epidemiology of this major public health challenge. The findings highlight the urgent need for ongoing vigilance and the implementation of focused public health measures to combat the persistent challenge of tobacco-related ischemic stroke in Pakistan. Our findings underscore a concerning increase of 38.13% in stroke deaths due to tobacco over the study period, which aligns with global trends observed in other Global Burden of Disease (GBD) studies. For instance, comparable studies have demonstrated the global impact of smoking and secondhand smoke on the burden of ischemic heart disease (IHD) and stroke, highlighting similar patterns of gender disparities and the differential impacts of direct and passive smoking^23,24^. Notably, Pakistan’s age-standardized mortality rate (ASMR) surpasses the global average, signaling a deepening health crisis that demands immediate attention and intervention.

Our study sheds light on the gender disparities in the incidence of tobacco-related ischemic stroke. A recent assessment of the economic ramifications of smoking-related diseases in Pakistan has brought to the fore a striking imbalance. It was discerned that approximately two-thirds of the aggregate economic burden engendered by smoking-related diseases and mortality in individuals aged ≥35 years is predominantly borne by the rural populace. Moreover, this analysis indicates that nearly 90% of this economic impact is sustained by men^25^. These observations highlight the pronounced and differential effects of smoking across various demographic segments within the nation. Further, our findings reveal a gender-specific pattern in tobacco exposure and its consequent health outcomes. We observed that men are primarily affected by strokes associated with active smoking, while women are more susceptible to the effects of secondhand smoke, which echoes findings from other regions. A study by Zheng et al.^26^ on the impact of smoking on morbidity in China found analogous gender disparities, indicating a universal trend that necessitates gender-sensitive interventions in tobacco control policies. This distinct divergence necessitates the implementation of gender-targeted public health interventions. The elevated incidence of active smoking among men and its direct correlation with a higher stroke rate underscores the critical need for male-centered smoking cessation programs. Conversely, the significant influence of secondhand smoke on women amplifies the call for comprehensive public health strategies.

Age emerges as a critical factor in the tobacco-attributable stroke burden, with the most significant increase in deaths and mortality rates noted among those aged ≥70 years. This is consistent with the study by Wu et al.^27^ who produced longitudinal age curves documenting increases in CVD, IHD, and stroke mortality from smoking around the world. This trend suggests the cumulative impact of tobacco exposure over a lifetime, highlighting the importance of early and sustained tobacco control measures.

The study also reveals regional disparities within Pakistan, with the highest rates of tobacco-attributable ASMR and ASDR found in Punjab and the lowest in Gilgit-Baltistan. The ASDR caused by secondhand smoke among women in Khyber Pakhtunkhwa Province has shown a significant upward trend in recent years (APC=0.17 from 2010 to 2019). However, in other regions, the disease burden of ischemic stroke attributable to active smoking and secondhand smoke has been declining in recent years (APC<0) in both genders. GBD 2019 Pakistan Collaborators also pointed out that although the life expectancy of both men and women has increased in all regions of Pakistan, it is worth noting that Khyber Pakhtunkhwa has the lowest increase in life expectancy^28^. These variations indicate the need for region-specific tobacco control policies and health interventions tailored to the unique needs and contexts of different regions of Pakistan.

Our study has significant implications for public health policy in Pakistan. The rising burden of tobacco-related stroke, especially among specific demographics and regions, necessitates focused interventions^29^. These should encompass comprehensive tobacco control policies, public education campaigns, smoking cessation programs, and measures to combat secondhand smoke exposure. However, what we need to pay attention to is that tobacco research or policies must be implemented in earnest and not just superficial^30^. Additionally, our study highlights the need for further research into the drivers behind gender and age disparities in tobacco-attributable stroke. Such research could guide more effective and personalized public health interventions.

### Limitations

Although our study contributes valuable insights, it has some limitations. Primarily, it is contingent upon data derived from the Global Burden of Disease (GBD) database, which does not offer detailed subclassification of ischemic stroke types. Existing literature indicates that in Pakistan, large vessel stroke is the most prevalent subtype, constituting 31.7% of stroke cases, followed by small vessel disease (25.7%) and cardioembolic stroke (10.4%). The cause of ischemic stroke was unknown in nearly 32% of subjects^31^. A significant gap in our understanding pertains to the specific subtypes of ischemic stroke associated with smoking, an aspect that is yet to be elucidated and represents a pivotal area for future epidemiological research. On the other hand, risk factors cluster within individuals and cannot be addressed in isolation^32^. Our study is based on the GBD database, so residual confounding factors cannot be avoided. In addition, because the GBD database does not fully summarize the data of various regions within the country, it may not be universally applicable to countries that only have data for the country but do not have specific data for each region of the country (such as China, etc.). These countries often need to be compared with the national data and with those of relevant CDCs. In addition, considering the real-world context, the challenge of modeling the impact of tobacco use on stroke rates due to varying smoking cessation policies complicates predictions.

## CONCLUSIONS

Smoking tobacco significantly elevates the risk for numerous diseases and related deaths, yet it represents behavior that can be changed^33,34^. This study offers evidence to support regional governments in implementing policies aimed at reducing tobacco-related disease burdens. Looking forward, ongoing monitoring of tobacco-related stroke trends in Pakistan is essential. Longitudinal studies are valuable for assessing the long-term impact of public health interventions. Moreover, investigating the socio-economic and cultural factors influencing tobacco use in Pakistan could yield insights vital for designing more effective tobacco control strategies.

## Supplementary Material



## Data Availability

The data supporting this research are available from the authors on reasonable request.
