# Supplementary material for: Assessing tobacco-related ischemic stroke in Pakistan (1990–2019): Insights from the Global Burden of Disease Study
Source: Tob Induc Dis. 2024 Mar 28;22:10.18332/tid/185566. doi: 10.18332/tid/185566 (PMC10973799; doi:10.18332/tid/185566)
Supplement: Supplementary file 1 [file TID-22-55-s1.pdf]

Supplementary Tables:

Supplementary Table 1. Disease burden and ranking attributable to tobacco use in Pakistan in 2019.

Supplementary Table 2. Disease burden and ranking attributable to active smoking in Pakistan in 2019.

Supplementary Table 3. Disease burden and ranking attributable to secondhand smoke in Pakistan in 2019.

Supplementary Table 4. Average annual percentage change (AAPC) in ASMR and ASDR for tobacco-related (tobacco use, active smoking and second-hand smoke) attributable ischemic stroke in Pakistan, 1990-2019.

Supplementary Table 5. Average annual percentage change (AAPC) in ASMR and ASDR for tobacco-related (tobacco use, active smoking, and second-hand smoke) attributable ischemic stroke across regions of Pakistan from 1990 to 2019.

Supplementary Table 1. Disease burden and ranking attributable to tobacco use in Pakistan in 2019.

Diseases Attributable to Tobacco Use	Deaths		DALYs	
	ASMR	Ranking	ASDR	Ranking
Acute lymphoid leukemia	0.03 (0.01 to 0.05)	16	0.81 (0.33 to 1.59)	21
Acute myeloid leukemia	0.14 (0.08 to 0.23)	10	3.32 (1.68 to 5.79)	11
Age-related macular degeneration	NA	NA	1.21 (0.42 to 2.06)	17
Cataract	NA	NA	20.88 (14.14 to 29.97)	7
Chronic lymphoid leukemia	0.1 (0.05 to 0.18)	12	2.23 (1.08 to 3.91)	13
Chronic myeloid leukemia	0.15 (0.08 to 0.24)	9	3.66 (1.72 to 6.4)	9
Cyclist road injuries	0 (0 to 0)	25	0.61 (0.4 to 0.91)	22
Diabetes mellitus type 2	7.77 (4.65 to 11.08)	2	259.96 (163.04 to 357.72)	1
Drug-susceptible tuberculosis	6.27	3	195.84	3

Diseases Attributable to Tobacco Use	Deaths		DALYs	
	ASMR	Ranking	ASDR	Ranking
	(4.04 to 9.28)		(128.66 to 287.65)	
Extensively drug-resistant tuberculosis	0.04<br>(0.01 to 0.11)	15	1.21<br>(0.3 to 3.35)	16
Intracerebral hemorrhage	8.56<br>(6.78 to 10.76)	1	236.15<br>(188.21 to 298.07)	2
Ischemic stroke	6.04<br>(4.7 to 8.11)	4	130.81<br>(103.9 to 174.17)	4
Liver cancer due to alcohol use	0.07<br>(0.03 to 0.11)	13	1.66<br>(0.68 to 2.9)	14
Liver cancer due to hepatitis B	0.05<br>(0.02 to 0.09)	14	1.4<br>(0.52 to 2.56)	15
Liver cancer due to hepatitis C	0.16<br>(0.07 to 0.26)	8	3.53<br>(1.54 to 5.89)	10
Liver cancer due to NASH	0.02<br>(0.01 to 0.03)	18	0.46<br>(0.19 to 0.78)	24
Liver cancer due to other causes	0.01<br>(0 to 0.01)	23	0.18<br>(0.07 to 0.32)	27
Motor vehicle road injuries	0.01<br>(0.01 to 0.02)	19	0.87<br>(0.55 to 1.31)	20
Motorcyclist road injuries	0.01	22	1.01	18

Diseases Attributable to Tobacco Use	Deaths		DALYs	
	ASMR	Ranking	ASDR	Ranking
	(0.01 to 0.02)		(0.66 to 1.52)	
Multidrug-resistant tuberculosis without extensive drug resistance	0.8<br>(0.2 to 2.24)	6	23.95<br>(6.13 to 67.39)	6
Non-venomous animal contact	0.01<br>(0 to 0.01)	24	0.21<br>(0.12 to 0.34)	26
Other exposure to mechanical forces	0.01<br>(0.01 to 0.02)	21	0.38<br>(0.25 to 0.57)	25
Other leukemia	0.13<br>(0.07 to 0.21)	11	2.69<br>(1.41 to 4.35)	12
Other road injuries	0<br>(0 to 0)	26	0.06<br>(0.03 to 0.09)	28
Pedestrian road injuries	0.02<br>(0.01 to 0.03)	17	0.92<br>(0.63 to 1.31)	19
Peptic ulcer disease	0.26<br>(0.18 to 0.39)	7	6.78<br>(4.77 to 9.73)	8
Physical violence by other means	0.01<br>(0.01 to 0.02)	20	0.55<br>(0.33 to 0.85)	23
Subarachnoid hemorrhage	1.28<br>(0.71 to 2.08)	5	40.8<br>(23.92 to 65.85)	5

Supplementary Table 2. Disease burden and ranking attributable to active smoking in Pakistan in 2019.

Diseases Attributable to Active smoking	Deaths		DALYs	
	ASMR	Ranking	ASDR	Ranking
Acute lymphoid leukemia	0.03<br>(0.01 to 0.05)	16	0.81<br>(0.33 to 1.59)	21
Acute myeloid leukemia	0.14<br>(0.08 to 0.23)	10	3.32<br>(1.68 to 5.79)	11
Age-related macular degeneration	NA	NA	1.21<br>(0.42 to 2.06)	17
Cataract	NA	NA	20.88<br>(14.14 to 29.97)	7
Chronic lymphoid leukemia	0.1<br>(0.05 to 0.18)	12	2.23<br>(1.08 to 3.91)	13
Chronic myeloid leukemia	0.15<br>(0.08 to 0.24)	9	3.66<br>(1.72 to 6.4)	9
Cyclist road injuries	0<br>(0 to 0)	25	0.61<br>(0.4 to 0.91)	22
Diabetes mellitus type 2	3.15<br>(2.25 to 4.27)	4	115.67<br>(87.82 to 150.48)	3
Drug-susceptible tuberculosis	6.27<br>(4.04 to 9.28)	2	195.84<br>(128.66 to 287.65)	1
Extensively drug-resistant tuberculosis	0.04	15	1.21	16

Diseases Attributable to Active smoking	Deaths		DALYs	
	ASMR	Ranking	ASDR	Ranking
	(0.01 to 0.11)		(0.3 to 3.35)	
Intracerebral hemorrhage	6.84<br>(5.32 to 8.78)	1	189.13<br>(146.45 to 243.86)	2
Ischemic stroke	4.78<br>(3.63 to 6.63)	3	106.34<br>(82.07 to 144.36)	4
Liver cancer due to alcohol use	0.07<br>(0.03 to 0.11)	13	1.66<br>(0.68 to 2.9)	14
Liver cancer due to hepatitis B	0.05<br>(0.02 to 0.09)	14	1.4<br>(0.52 to 2.56)	15
Liver cancer due to hepatitis C	0.16<br>(0.07 to 0.26)	8	3.53<br>(1.54 to 5.89)	10
Liver cancer due to NASH	0.02<br>(0.01 to 0.03)	18	0.46<br>(0.19 to 0.78)	24
Liver cancer due to other causes	0.01<br>(0 to 0.01)	23	0.18<br>(0.07 to 0.32)	27
Motor vehicle road injuries	0.01<br>(0.01 to 0.02)	19	0.87<br>(0.55 to 1.31)	20
Motorcyclist road injuries	0.01<br>(0.01 to 0.02)	22	1.01<br>(0.66 to 1.52)	18
Multidrug-resistant tuberculosis without	0.8	6	23.95	6

Diseases Attributable to Active smoking	Deaths		DALYs	
	ASMR	Ranking	ASDR	Ranking
extensive drug resistance	(0.2 to 2.24)		(6.13 to 67.39)	
Non-venomous animal contact	0.01<br>(0 to 0.01)	24	0.21<br>(0.12 to 0.34)	26
Other exposure to mechanical forces	0.01<br>(0.01 to 0.02)	21	0.38<br>(0.25 to 0.57)	25
Other leukemia	0.13<br>(0.07 to 0.21)	11	2.69<br>(1.41 to 4.35)	12
Other road injuries	0<br>(0 to 0)	26	0.06<br>(0.03 to 0.09)	28
Pedestrian road injuries	0.02<br>(0.01 to 0.03)	17	0.92<br>(0.63 to 1.31)	19
Peptic ulcer disease	0.26<br>(0.18 to 0.39)	7	6.78<br>(4.77 to 9.73)	8
Physical violence by other means	0.01<br>(0.01 to 0.02)	20	0.55<br>(0.33 to 0.85)	23
Subarachnoid hemorrhage	1<br>(0.54 to 1.69)	5	31.56<br>(17.85 to 53.3)	5

Supplementary Table 3. Disease burden and ranking attributable to secondhand smoke in Pakistan in 2019.

Diseases Attributable to Secondhand smoke	Deaths		DALYs	
	ASMR	Ranking	ASDR	Ranking
Diabetes mellitus type 2	4.89 (1.83 to 7.87)	1	154.06 (56.22 to 243.51)	1
Intracerebral hemorrhage	1.95 (1.33 to 2.76)	2	53.94 (36.12 to 75.3)	2
Ischemic stroke	1.39 (0.94 to 1.99)	3	27.67 (18.9 to 39.33)	3
Subarachnoid hemorrhage	0.32 (0.17 to 0.51)	4	10.49 (5.69 to 16.39)	4

Supplementary Table 4. Average annual percentage change (AAPC) in ASMR and ASDR for tobacco-related (tobacco use, active smoking and second-hand smoke) attributable ischemic stroke in Pakistan, 1990-2019.

Risk factor	Measure	AAPC		
		Both	Male	Female
Tobacco use				
	Death	-0.81 (-0.88 to -0.74)*	-0.78 (-0.84 to -0.72)*	-0.37 (-0.43 to -0.31)*
	DALY	-0.68 (-0.75 to -0.61)*	-0.6 (-0.66 to -0.54)*	-0.48 (-0.55 to -0.41)*
Active smoking				
	Death	-1.01 (-1.14 to -0.89)*	-0.85 (-0.91 to -0.79)*	-0.64 (-0.78 to -0.5)*
	DALY	-0.84 (-0.91 to -0.78)*	-0.67 (-0.74 to -0.61)*	-0.72 (-0.83 to -0.6)*
Secondhand smoke				
	Death	-0.10 (-0.15 to -0.05)*	-0.23 (-0.3 to -0.16)*	-0.14 (-0.23 to -0.05)*
	DALY	-0.06	0.00	-0.21

	(-0.1 to -0.02)*	(-0.06 to 0.07)	(-0.3 to -0.12)*

Supplementary Table 5. Average annual percentage change (AAPC) in ASMR and ASDR for tobacco-related (tobacco use, active smoking, and second-hand smoke) attributable ischemic stroke across regions of Pakistan from 1990 to 2019.

Risk factor	Location	Measure	AAPC		
			Both	Male	Female
Tobacco use					
	Azad Jammu & Kashmir	Death	-0.84 (-0.91 to -0.77)*	-0.76 (-0.86 to -0.66)*	-0.33 (-0.42 to -0.24)*
		DALY	-0.76 (-0.84 to -0.68)*	-0.62 (-0.72 to -0.53)*	-0.49 (-0.6 to -0.38)*
	Balochistan	Death	-0.43 (-0.57 to -0.29)*	-0.7 (-0.83 to -0.56)*	0.09 (-0.07 to 0.25)
		DALY	-0.38 (-0.52 to -0.24)*	-0.53 (-0.66 to -0.4)*	-0.04 (-0.21 to 0.13)
	Gilgit-Baltistan	Death	-0.37 (-0.66 to -0.08)*	-0.65 (-0.86 to -0.43)*	0.27 (0.02 to 0.51)*
		DALY	-0.29 (-0.54 to -0.04)*	-0.5 (-0.71 to -0.28)*	0.13 (-0.08 to 0.35)
	Islamabad Capital Territory	Death	-1.15 (-1.26 to -1.05)*	-1.14 (-1.29 to -0.99)*	-0.95 (-1.15 to -0.76)*
		DALY	-1.03 (-1.16 to -0.91)*	-0.95 (-1.09 to -0.81)*	-1.03 (-1.16 to -0.9)*

Risk factor	Location	Measure	AAPC		
			Both	Male	Female
	Khyber Pakhtunkhwa	Death	-0.3 (-0.38 to -0.22)*	-0.33 (-0.42 to -0.24)*	0.13 (0.04 to 0.22)*
		DALY	-0.22 (-0.31 to -0.13)*	-0.18 (-0.27 to -0.09)*	-0.02 (-0.1 to 0.07)
	Punjab	Death	-0.97 (-1.07 to -0.87)*	-0.83 (-0.93 to -0.74)*	-0.65 (-0.75 to -0.55)*
		DALY	-0.82 (-0.91 to -0.73)*	-0.67 (-0.75 to -0.58)*	-0.71 (-0.8 to -0.62)*
	Sindh	Death	-0.71 (-0.79 to -0.62)*	-0.81 (-0.89 to -0.72)*	-0.13 (-0.21 to -0.05)*
		DALY	-0.59 (-0.67 to -0.5)*	-0.63 (-0.73 to -0.52)*	-0.22 (-0.34 to -0.11)*
Active smoking					
	Azad Jammu & Kashmir	Death	-1.11 (-1.22 to -1.01)*	-0.86 (-0.96 to -0.77)*	-0.71 (-0.85 to -0.56)*
		DALY	-1 (-1.08 to -0.91)*	-0.71 (-0.81 to -0.62)*	-0.89 (-1.02 to -0.76)*
	Balochistan	Death	-0.61 (-0.75 to -0.46)*	-0.8 (-0.94 to -0.67)*	-0.27 (-0.47 to -0.08)*
		DALY	-0.56	-0.63	-0.46

Risk factor	Location	Measure	AAPC		
			Both	Male	Female
			(-0.7 to -0.42)*	(-0.75 to -0.5)*	(-0.67 to -0.26)*
	Gilgit-Baltistan	Death	-0.63<br>(-0.88 to -0.38)*	-0.76<br>(-0.97 to -0.54)*	-0.13<br>(-0.36 to 0.1)
		DALY	-0.51<br>(-0.75 to -0.27)*	-0.6<br>(-0.81 to -0.39)*	-0.29<br>(-0.51 to -0.08)*
	Islamabad Capital Territory	Death	-1.42<br>(-1.55 to -1.28)*	-1.24<br>(-1.38 to -1.09)*	-1.4<br>(-1.69 to -1.12)*
		DALY	-1.25<br>(-1.36 to -1.13)*	-1.05<br>(-1.19 to -0.92)*	-1.47<br>(-1.76 to -1.18)*
	Khyber Pakhtunkhwa	Death	-0.6<br>(-0.68 to -0.53)*	-0.42<br>(-0.52 to -0.33)*	-0.2<br>(-0.39 to -0.01)*
		DALY	-0.44<br>(-0.52 to -0.36)*	-0.27<br>(-0.36 to -0.18)*	-0.39<br>(-0.59 to -0.19)*
	Punjab	Death	-1.17<br>(-1.26 to -1.09)*	-0.92<br>(-1.01 to -0.82)*	-0.93<br>(-1.06 to -0.81)*
		DALY	-0.99<br>(-1.06 to -0.92)*	-0.74<br>(-0.83 to -0.65)*	-0.98<br>(-1.06 to -0.9)*
	Sindh	Death	-0.84<br>(-0.96 to -0.72)*	-0.87<br>(-0.97 to -0.77)*	-0.24<br>(-0.39 to -0.09)*
		DALY	-0.7	-0.69	-0.34

Risk factor	Location	Measure	AAPC		
			Both	Male	Female
			(-0.83 to -0.57)*	(-0.79 to -0.58)*	(-0.48 to -0.21)*
Secondhand smoke					
	Azad Jammu & Kashmir	Death	0.15<br>(0.02 to 0.27)*	-0.06<br>(-0.18 to 0.06)	0.04<br>(-0.05 to 0.13)
		DALY	0.12<br>(-0.01 to 0.25)	0.12<br>(0.02 to 0.21)*	-0.11<br>(-0.2 to -0.02)*
	Balochistan	Death	0.23<br>(0.15 to 0.31)*	0.06<br>(-0.08 to 0.21)	0.39<br>(0.29 to 0.5)*
		DALY	0.33<br>(0.25 to 0.41)*	0.29<br>(0.12 to 0.46)*	0.37<br>(0.24 to 0.49)*
	Gilgit-Baltistan	Death	0.35<br>(0.02 to 0.67)*	0.14<br>(-0.08 to 0.36)	0.5<br>(0.19 to 0.81)*
		DALY	0.35<br>(0.16 to 0.55)*	0.34<br>(0.17 to 0.51)*	0.38<br>(0.12 to 0.64)*
	Islamabad Capital Territory	Death	-0.35<br>(-0.43 to -0.26)*	-0.37<br>(-0.48 to -0.26)*	-0.55<br>(-0.75 to -0.35)*
		DALY	-0.28<br>(-0.37 to -0.18)*	-0.13<br>(-0.25 to -0.01)*	-0.59<br>(-0.82 to -0.36)*
	Khyber Pakhtunkhwa	Death	0.6<br>(0.5 to 0.7)*	0.39<br>(0.29 to 0.49)*	0.44<br>(0.35 to 0.53)*

Risk factor	Location	Measure	AAPC		
			Both	Male	Female
		DALY	0.53 (0.46 to 0.61)*	0.55 (0.49 to 0.61)*	0.33 (0.25 to 0.41)*
	Punjab	Death	-0.27 (-0.32 to -0.22)*	-0.31 (-0.38 to -0.25)*	-0.4 (-0.46 to -0.34)*
		DALY	-0.21 (-0.25 to -0.17)*	-0.08 (-0.15 to 0)*	-0.42 (-0.47 to -0.37)*
	Sindh	Death	-0.14 (-0.19 to -0.09)*	-0.36 (-0.44 to -0.27)*	0 (-0.11 to 0.12)
		DALY	-0.09 (-0.13 to -0.04)*	-0.15 (-0.23 to -0.07)*	-0.07 (-0.19 to 0.04)

Supplementary Figures:

Supplementary Figure 1. Trends in disease burden due to active and secondhand smoking in Pakistan and different SDI regions globally.

Supplementary Figure 2. Trends in ischemic stroke deaths attributable to active smoking and secondhand smoke.

Supplementary Figure 3. Trends in ischemic stroke DALYs attributable to active smoking and secondhand smoke.

Supplementary Figure 4. APC of ASMR in ischemic stroke due to tobacco use, active smoking and second-hand smoke by gender in Pakistan from 1990 to 2019.

Supplementary Figure 5. APC of ASDR in ischemic stroke due to tobacco use, active smoking and second-hand smoke by gender in Pakistan from 1990 to 2019.

Supplementary Figure 6. APC of ASMR in ischemic stroke due to tobacco use, active smoking and second-hand smoke by gender in various regions of Pakistan from 1990 to 2019.

Supplementary Figure 7. APC of ASDR in ischemic stroke due to tobacco use, active smoking and second-hand smoke by gender in various regions of Pakistan from 1990 to 2019.

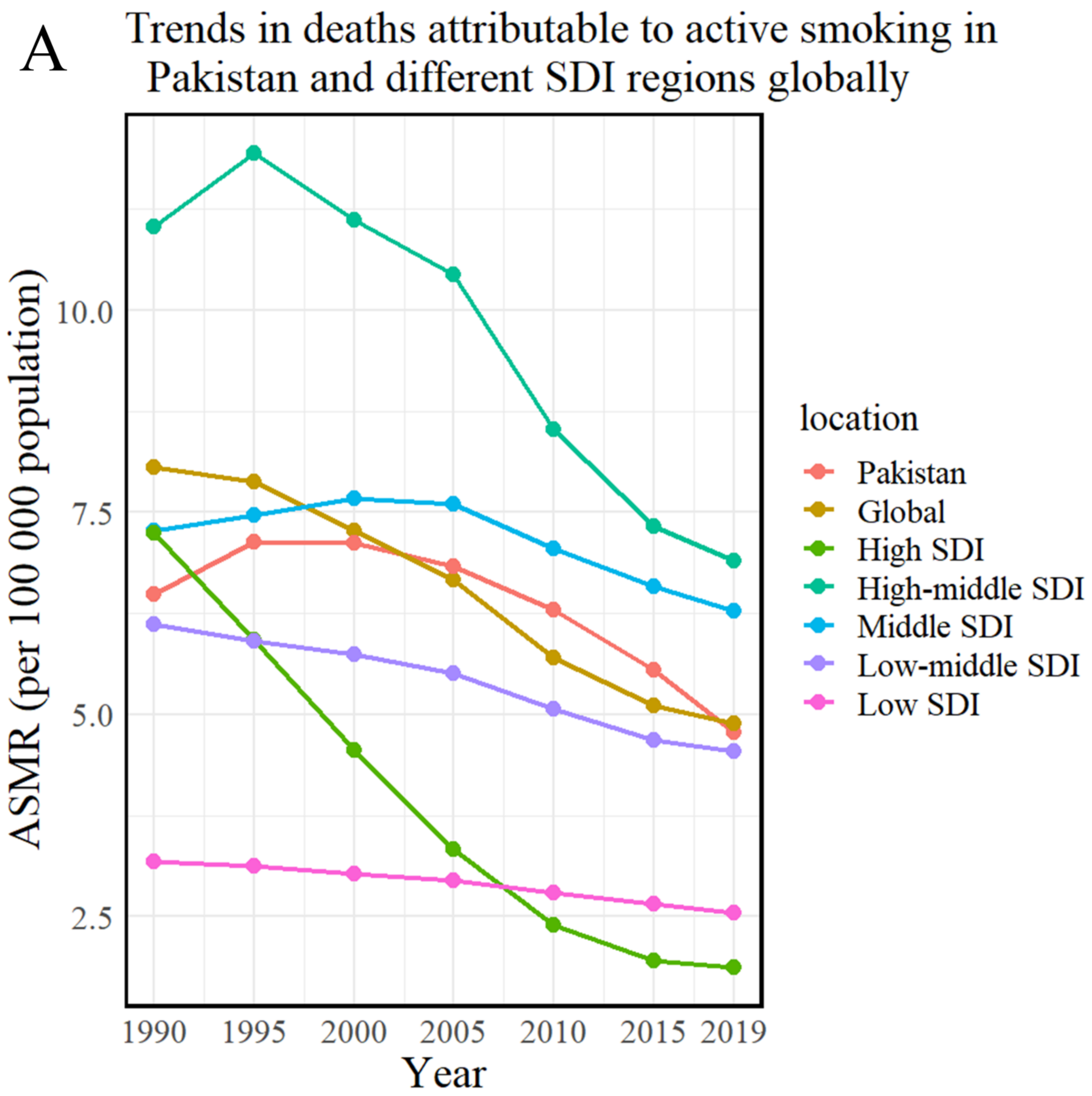
A
Trends in deaths attributable to active smoking in Pakistan and different SDI regions globally
ASMR (per 100 000 population)
10.0
7.5
5.0
2.5
1990
1995
2000
2005
2010
2015
2019
Year
location
Pakistan
Global
High SDI
High-middle SDI
Middle SDI
Low-middle SDI
Low SDI

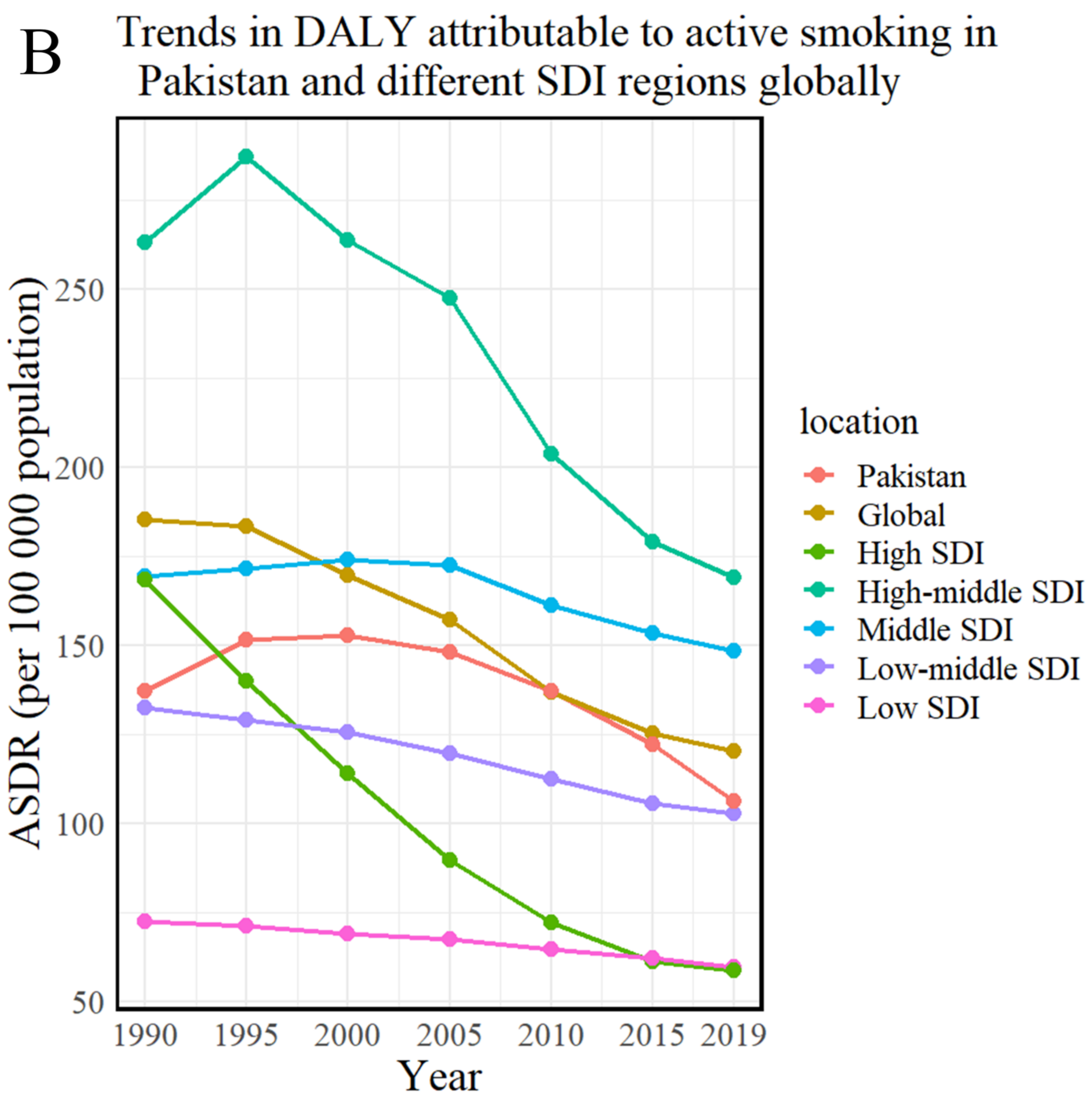
B
Trends in DALY attributable to active smoking in Pakistan and different SDI regions globally
ASDR (per 100 000 population)
250
200
150
100
50
1990
1995
2000
2005
2010
2015
2019
Year
location
Pakistan
Global
High SDI
High-middle SDI
Middle SDI
Low-middle SDI
Low SDI

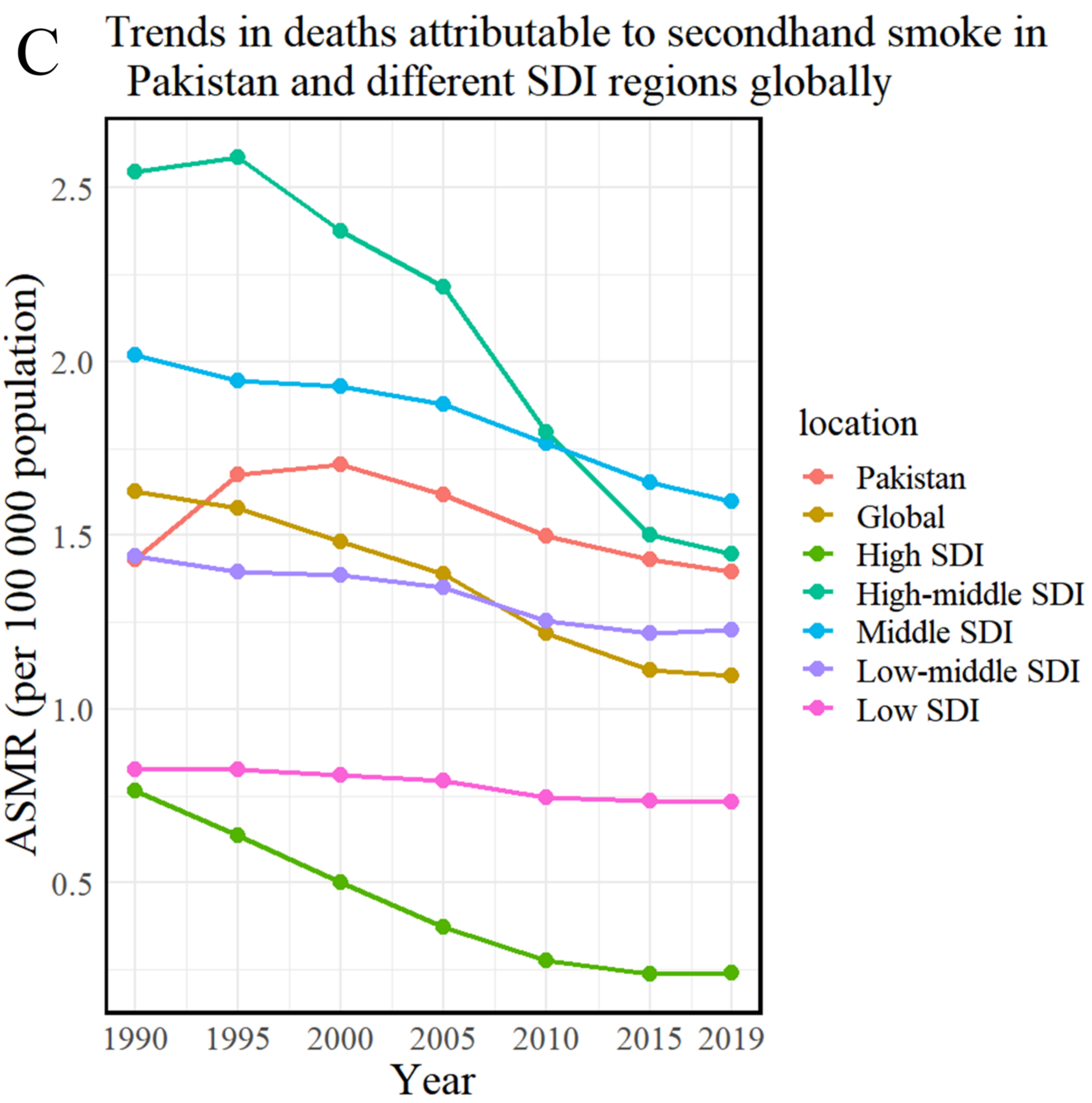

C
Trends in deaths attributable to secondhand smoke in Pakistan and different SDI regions globally
ASMR (per 100 000 population)
2.5
2.0
1.5
1.0
0.5
1990
1995
2000
2005
2010
2015
2019
Year
location
Pakistan
Global
High SDI
High-middle SDI
Middle SDI
Low-middle SDI
Low SDI

D

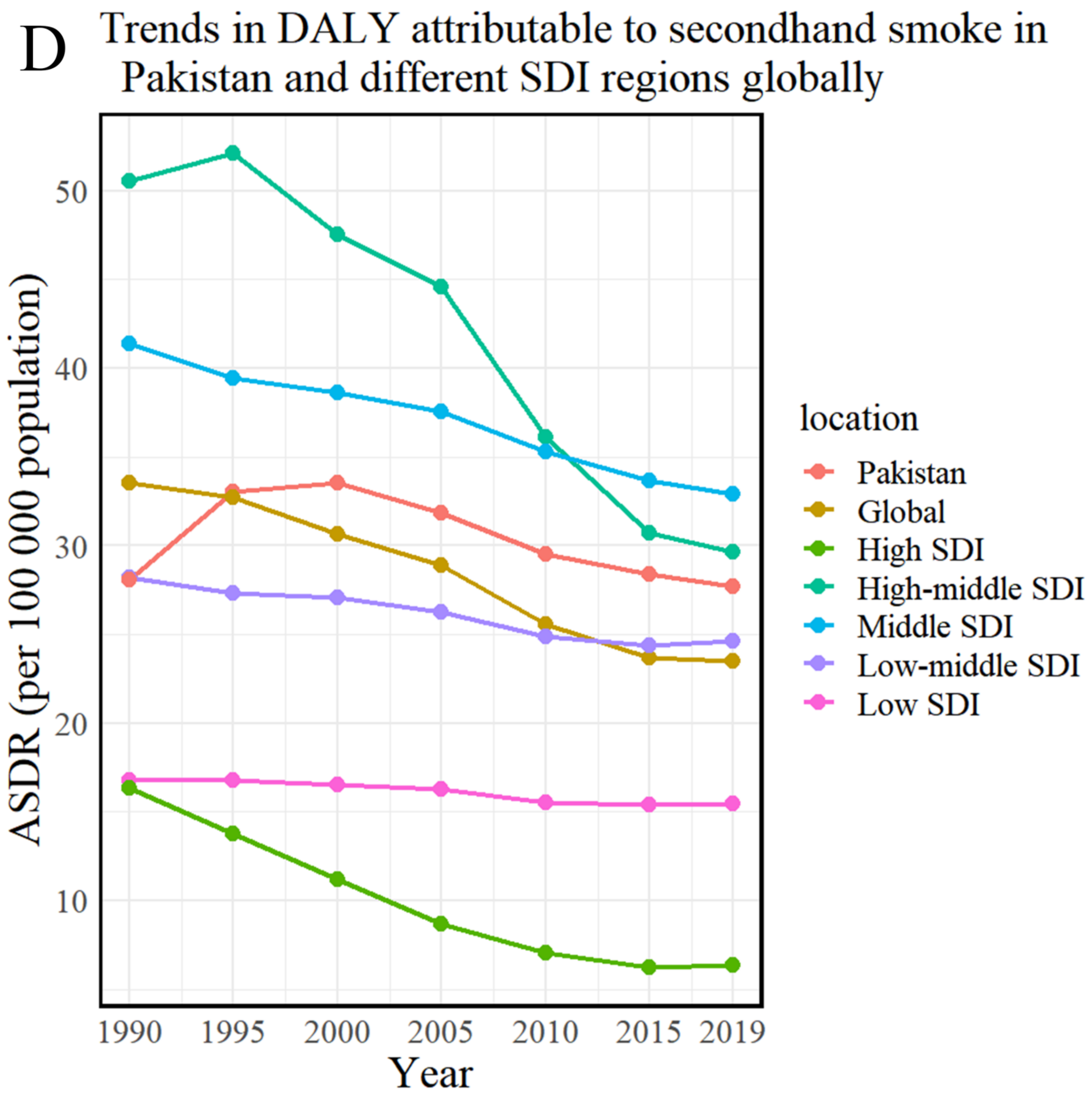

Trends in DALY attributable to secondhand smoke in Pakistan and different SDI regions globally
ASDR (per 100 000 population)
50
40
30
20
10
1990
1995
2000
2005
2010
2015
2019
Year
location
Pakistan
Global
High SDI
High-middle SDI
Middle SDI
Low-middle SDI
Low SDI

Supplementary Figure 1. Trends in disease burden due to active and secondhand smoking in Pakistan and different SDI regions globally.

A. Trends in deaths attributable to active smoking in Pakistan and different SDI regions globally.

B. Trends in DALY attributable to active smoking in Pakistan and different SDI regions globally.

C. Trends in deaths attributable to secondhand smoke in Pakistan and different SDI regions globally.

D. Trends in DALY attributable to secondhand smoke in Pakistan and different SDI regions globally.

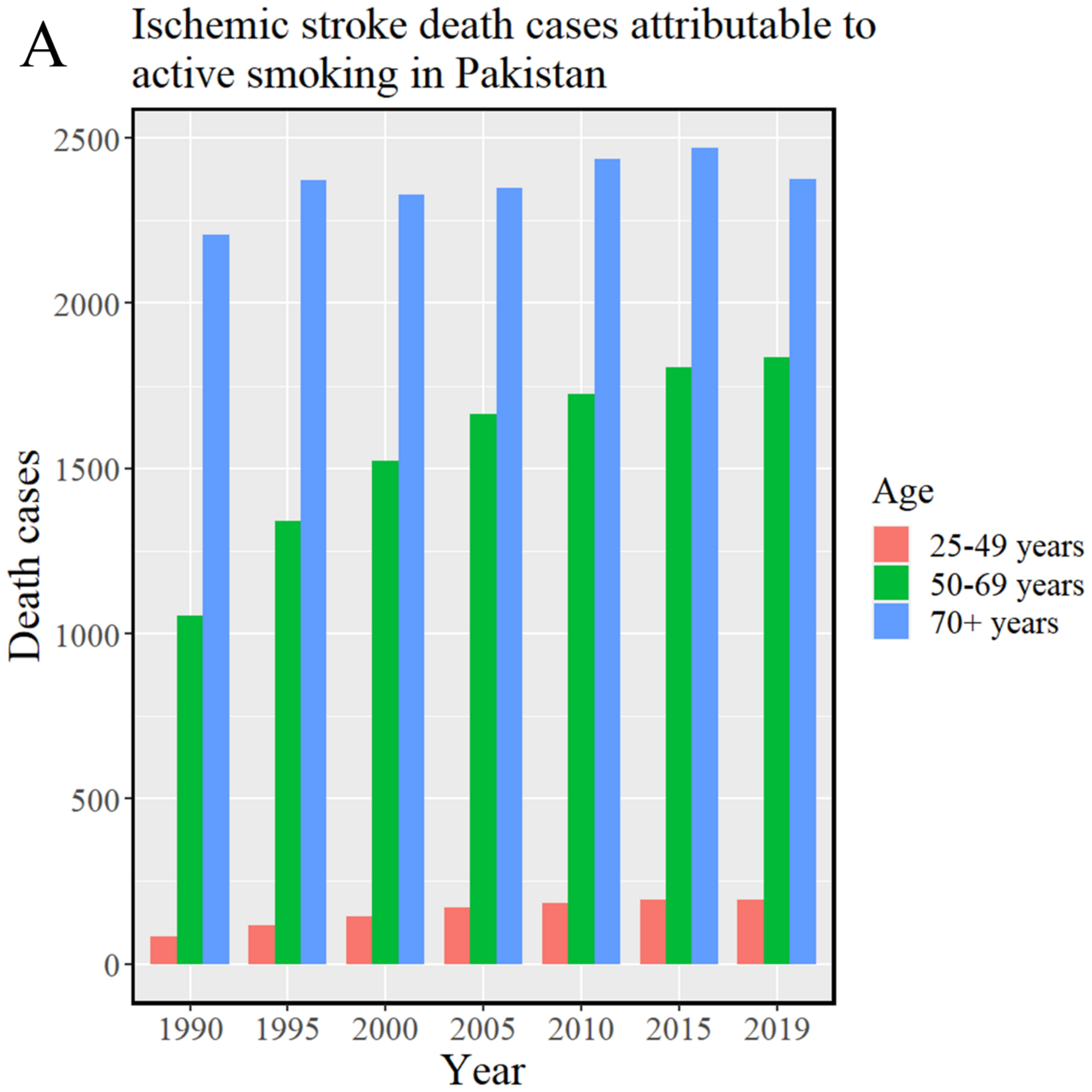
A
Ischemic stroke death cases attributable to active smoking in Pakistan
Death cases
2500
2000
1500
1000
500
0
1990
1995
2000
2005
2010
2015
2019
Year
Age
25-49 years
50-69 years
70+ years

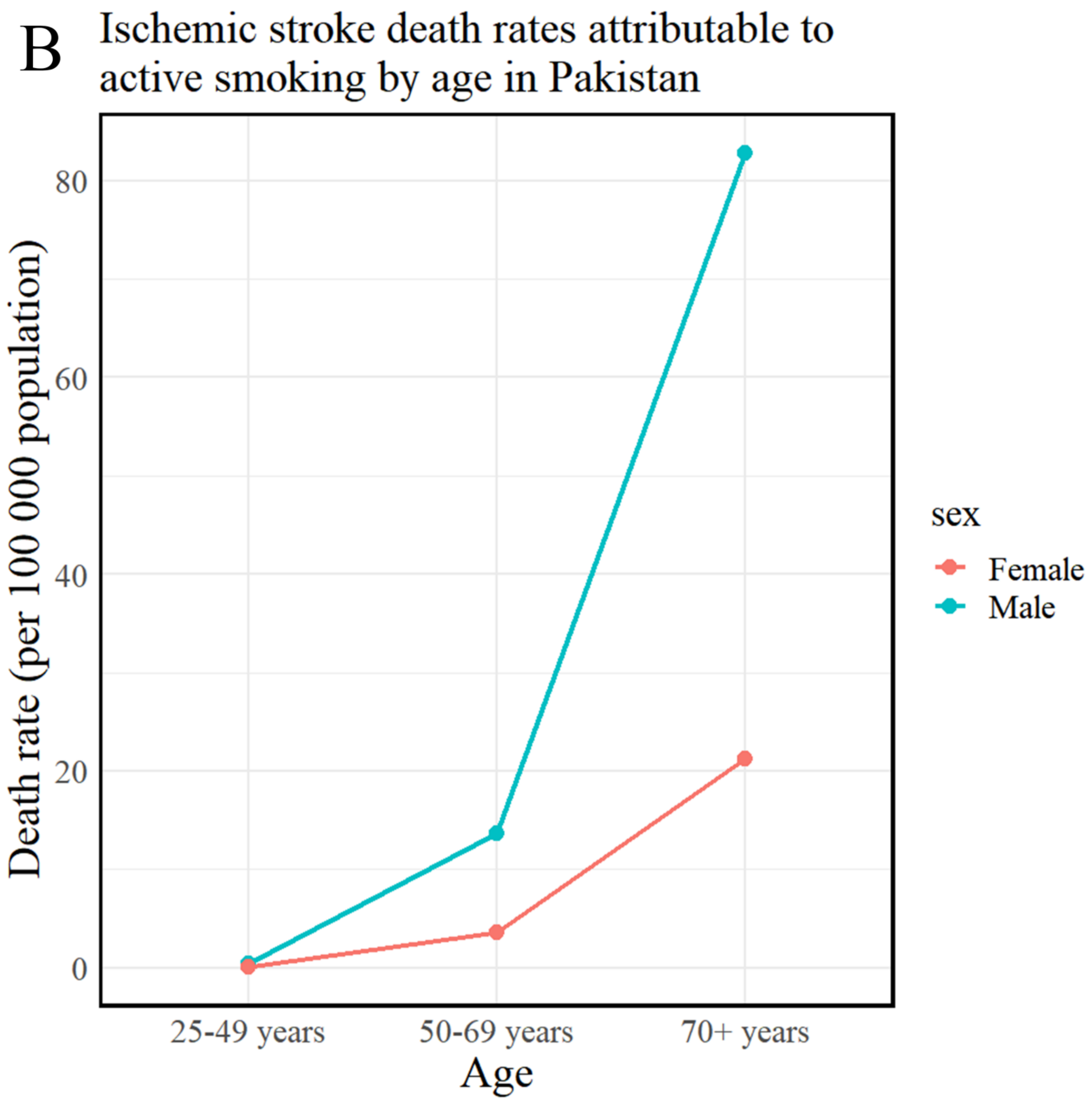

B
Ischemic stroke death rates attributable to active smoking by age in Pakistan
Death rate (per 100 000 population)
80
60
40
20
0
25-49 years
50-69 years
70+ years
Age
sex
Female
Male

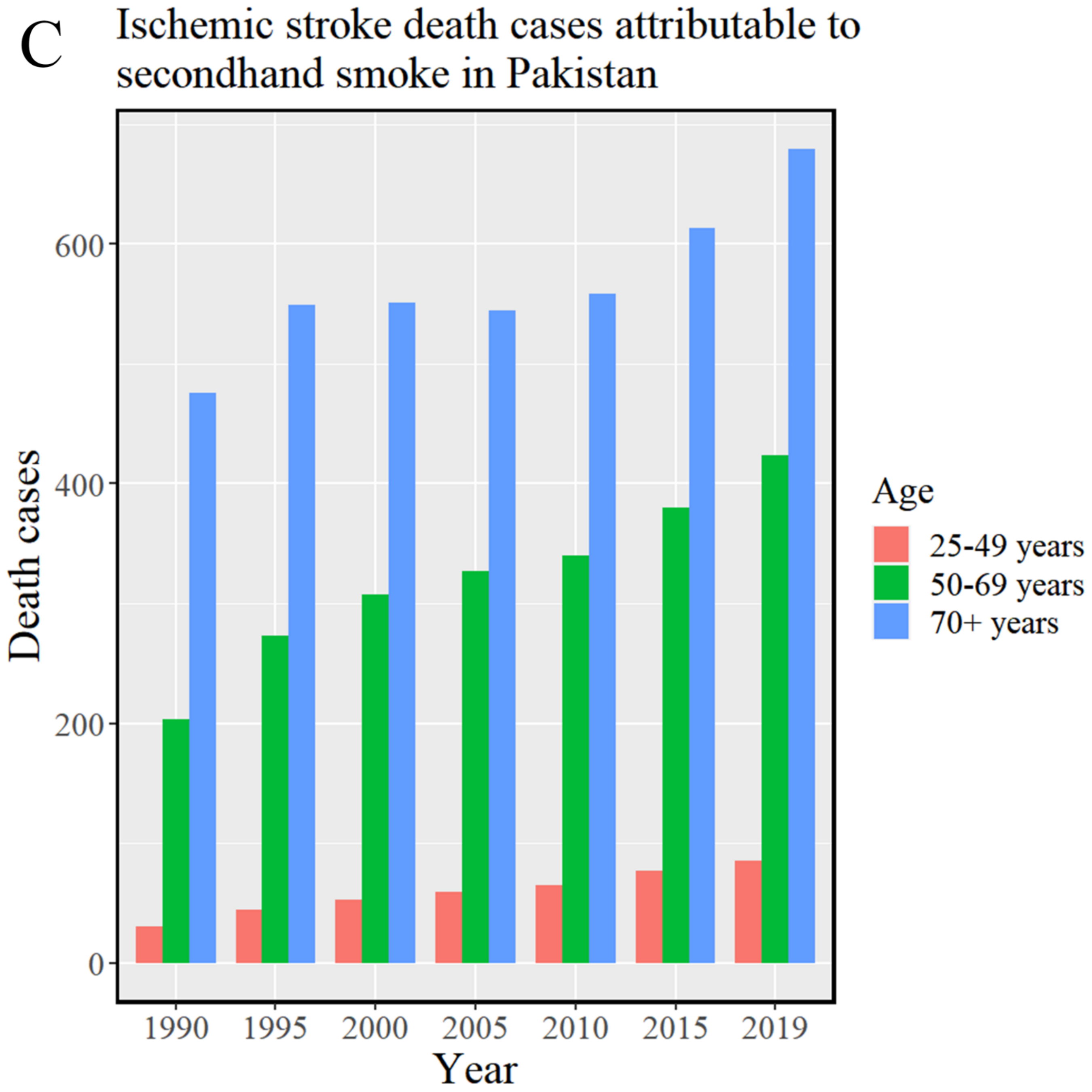
C
Ischemic stroke death cases attributable to secondhand smoke in Pakistan
Death cases
600
400
200
0
1990
1995
2000
2005
2010
2015
2019
Year
Age
25-49 years
50-69 years
70+ years

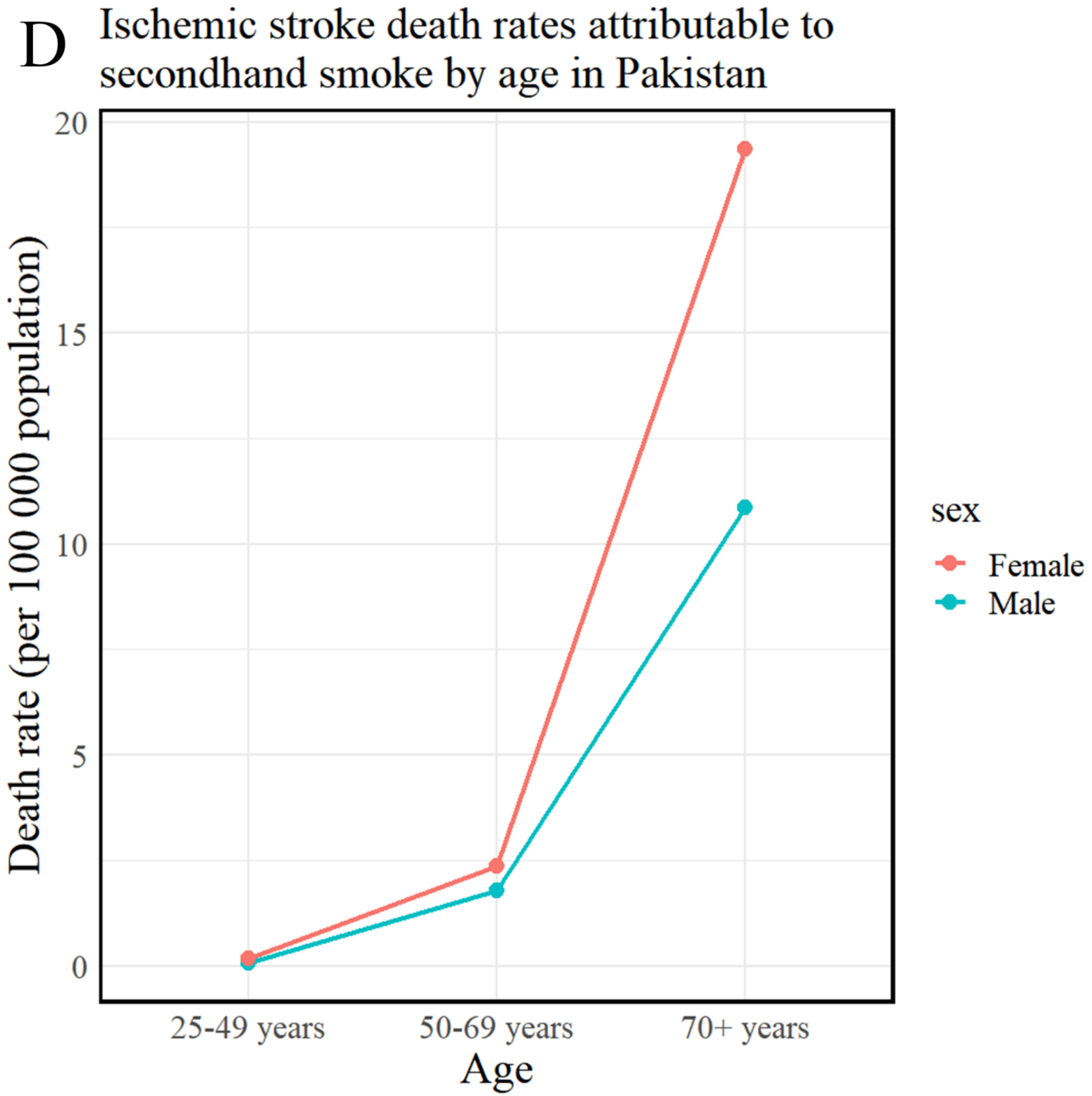
D
Ischemic stroke death rates attributable to secondhand smoke by age in Pakistan
Death rate (per 100 000 population)
20
15
10
5
0
25-49 years
50-69 years
70+ years
Age
sex
Female
Male

Supplementary Figure 2. Trends in ischemic stroke deaths attributable to active smoking and secondhand smoke.

A. Ischemic stroke death cases attributable to active smoking in Pakistan.

B. Ischemic stroke death rates attributable to active smoking by age in Pakistan.

C. Ischemic stroke death cases attributable to secondhand smoke in Pakistan.

D. Ischemic stroke death rates attributable to secondhand smoke by age in Pakistan.

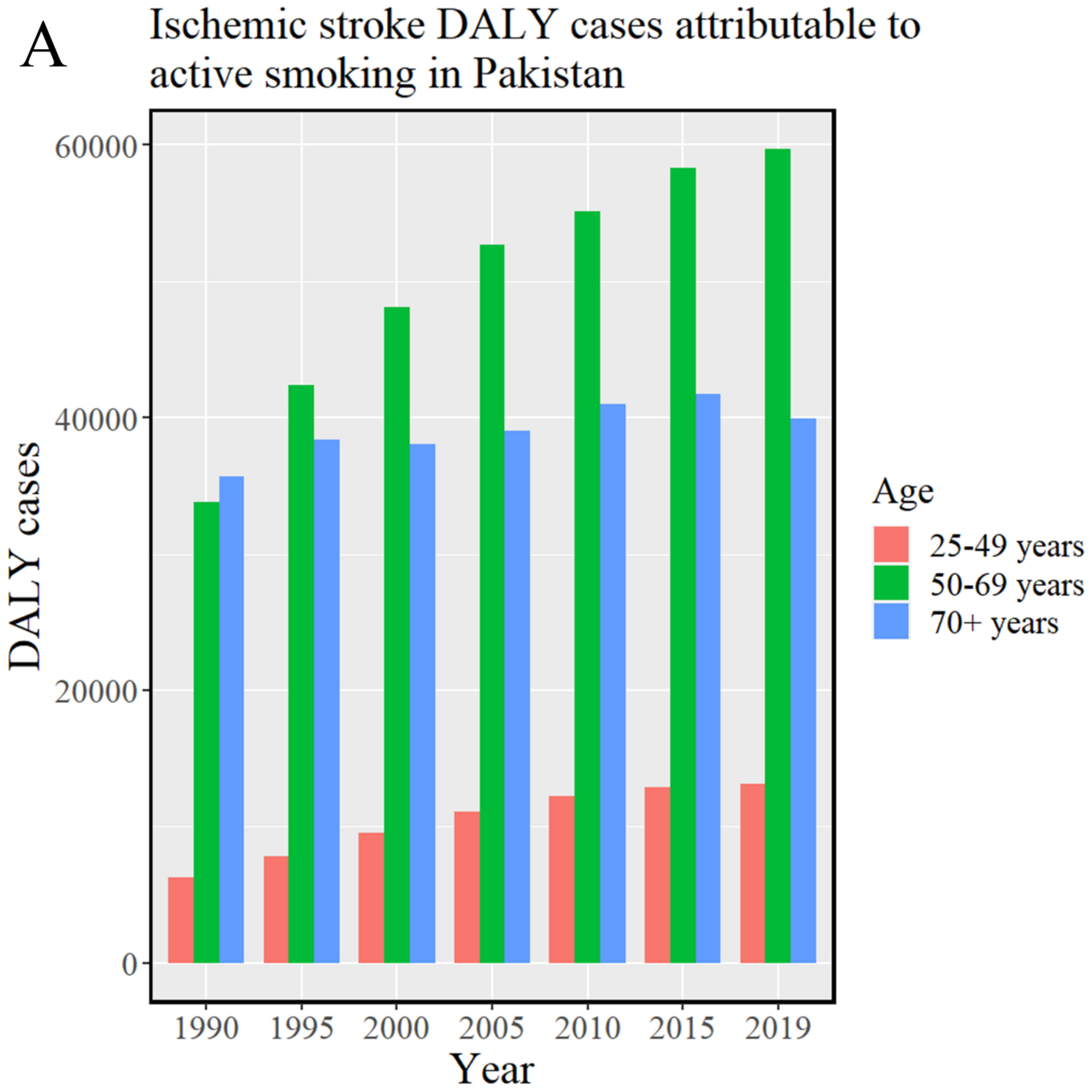
A
Ischemic stroke DALY cases attributable to active smoking in Pakistan
DALY cases
60000
40000
20000
0
1990
1995
2000
2005
2010
2015
2019
Year
Age
25-49 years
50-69 years
70+ years

B

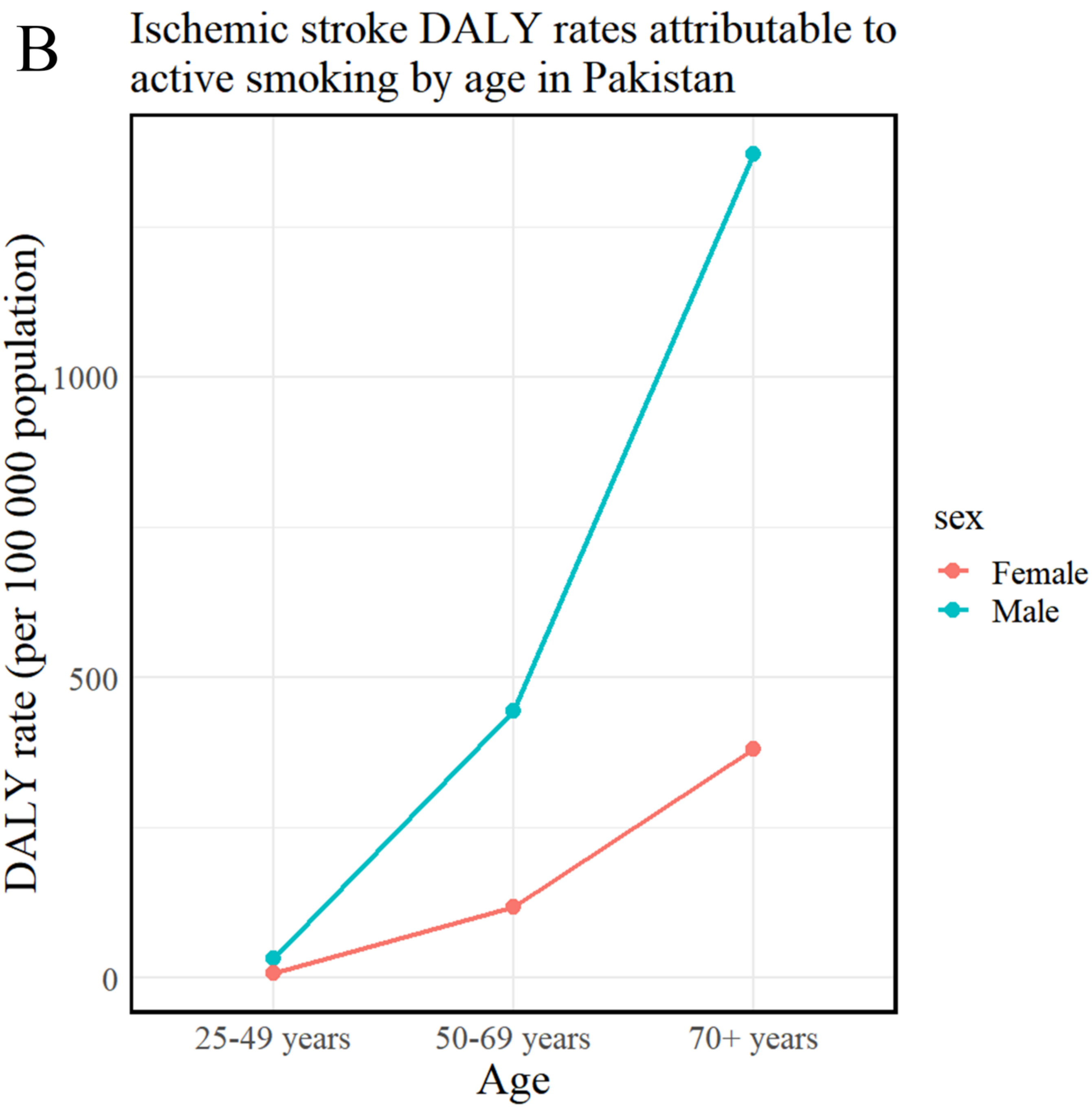
Ischemic stroke DALY rates attributable to active smoking by age in Pakistan
DALY rate (per 100 000 population)
1000
500
0
25-49 years
50-69 years
70+ years
Age
sex
Female
Male

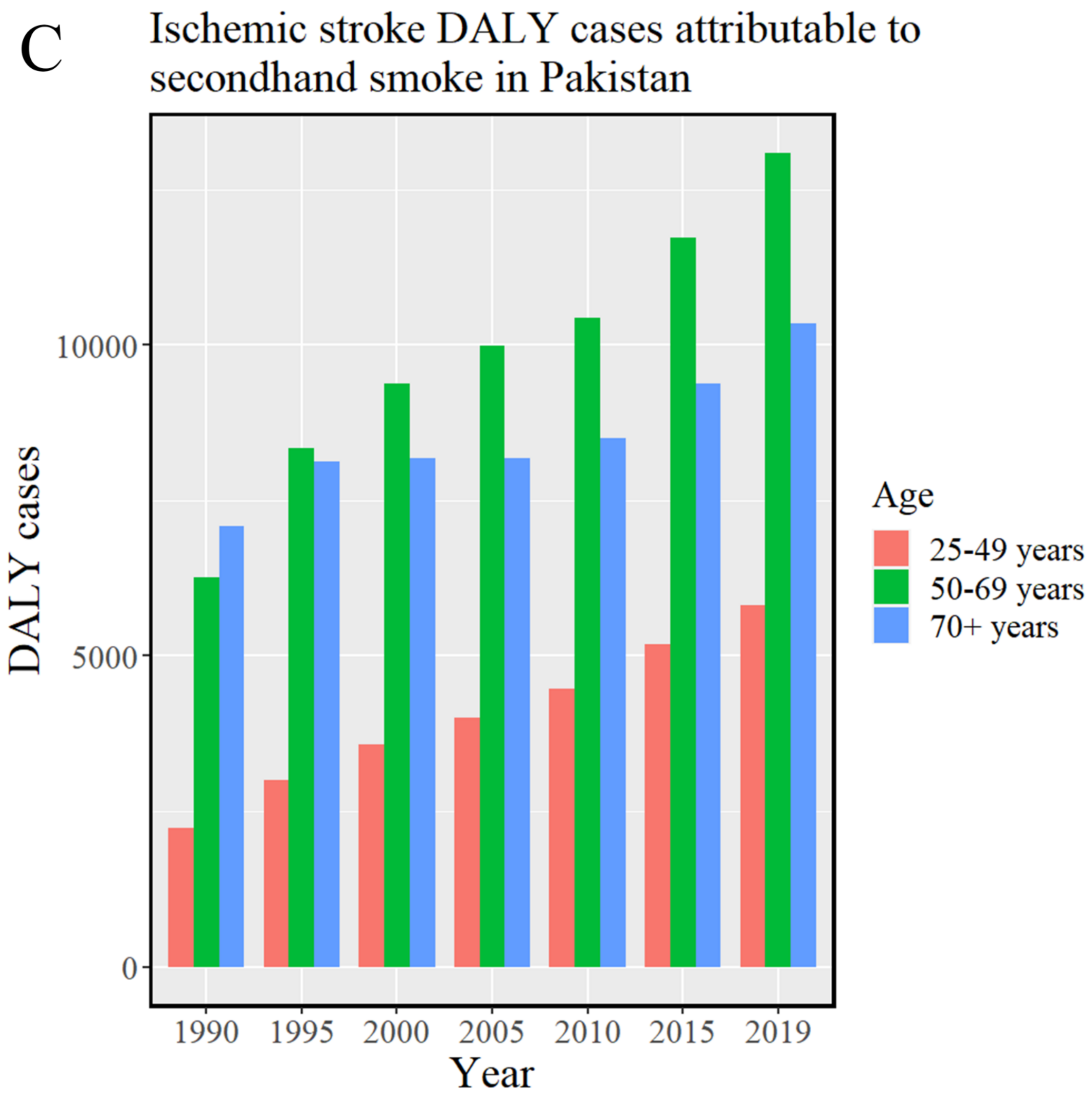

C
Ischemic stroke DALY cases attributable to secondhand smoke in Pakistan
DALY cases
10000
5000
0
1990
1995
2000
2005
2010
2015
2019
Year
Age
25-49 years
50-69 years
70+ years

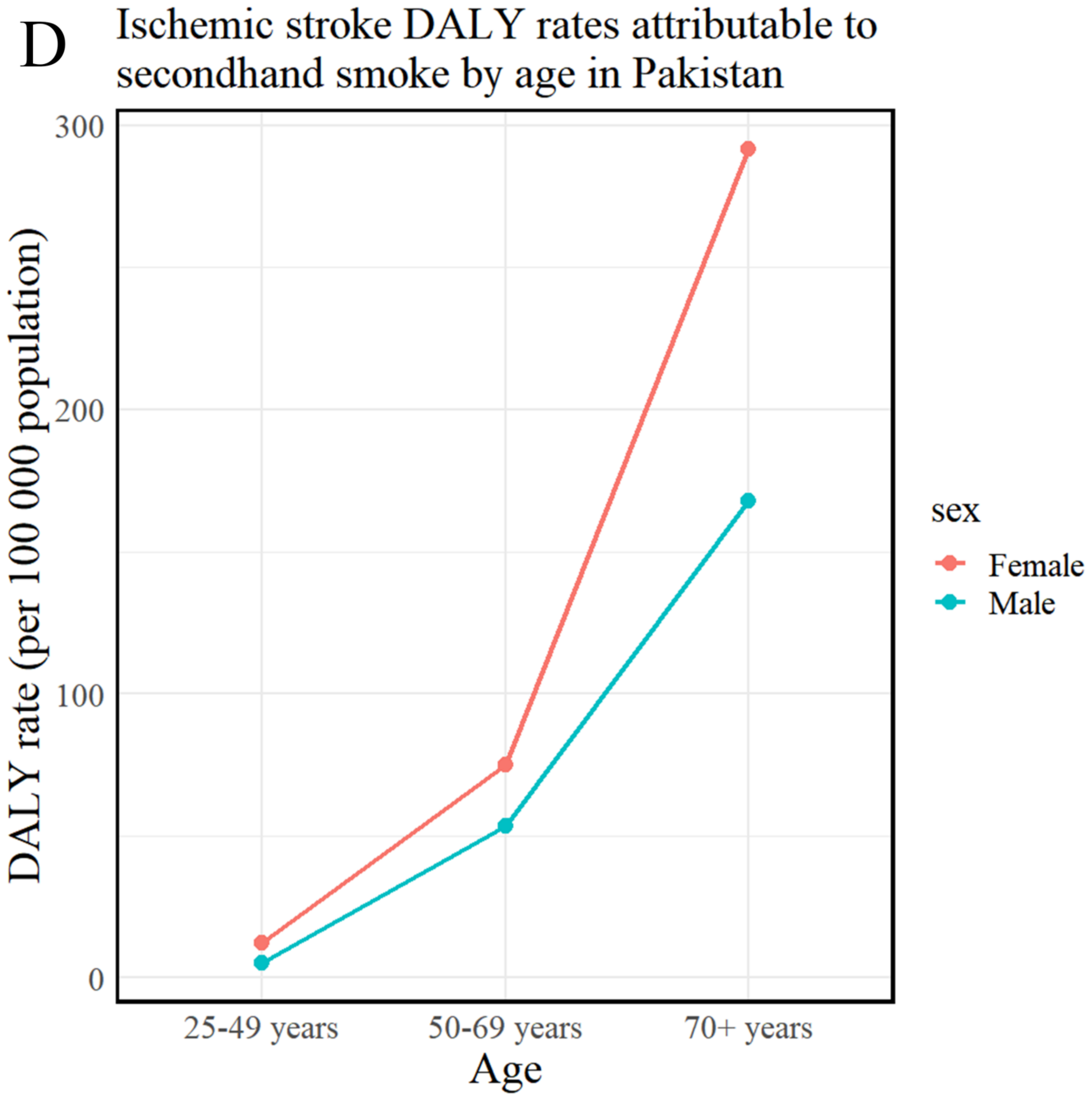
D
Ischemic stroke DALY rates attributable to secondhand smoke by age in Pakistan
300
200
100
0
DALY rate (per 100 000 population)
25-49 years
50-69 years
70+ years
Age
sex
Female
Male

Supplementary Figure 3. Trends in ischemic stroke DALYs attributable to active smoking and secondhand smoke.

A. Ischemic stroke DALY cases attributable to active smoking in Pakistan.

B. Ischemic stroke DALY rates attributable to active smoking by age in Pakistan.

C. Ischemic stroke DALY cases attributable to secondhand smoke in Pakistan.

D. Ischemic stroke DALY rates attributable to secondhand smoke by age in Pakistan.

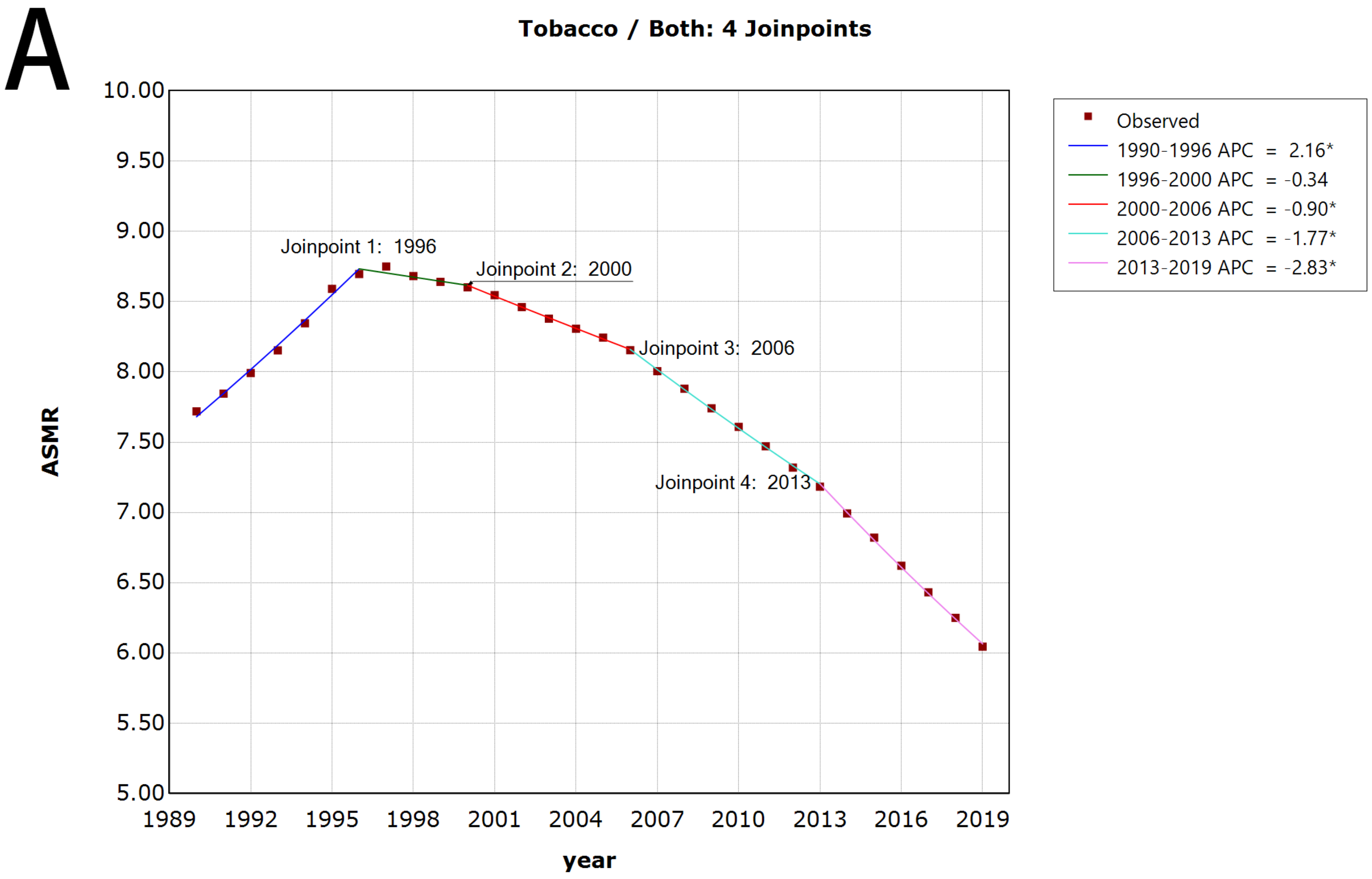

* Indicates that the Annual Percent Change (APC) is significantly different from zero at the alpha = 0.05 level
Final Selected Model: 4 Joinpoints.

B

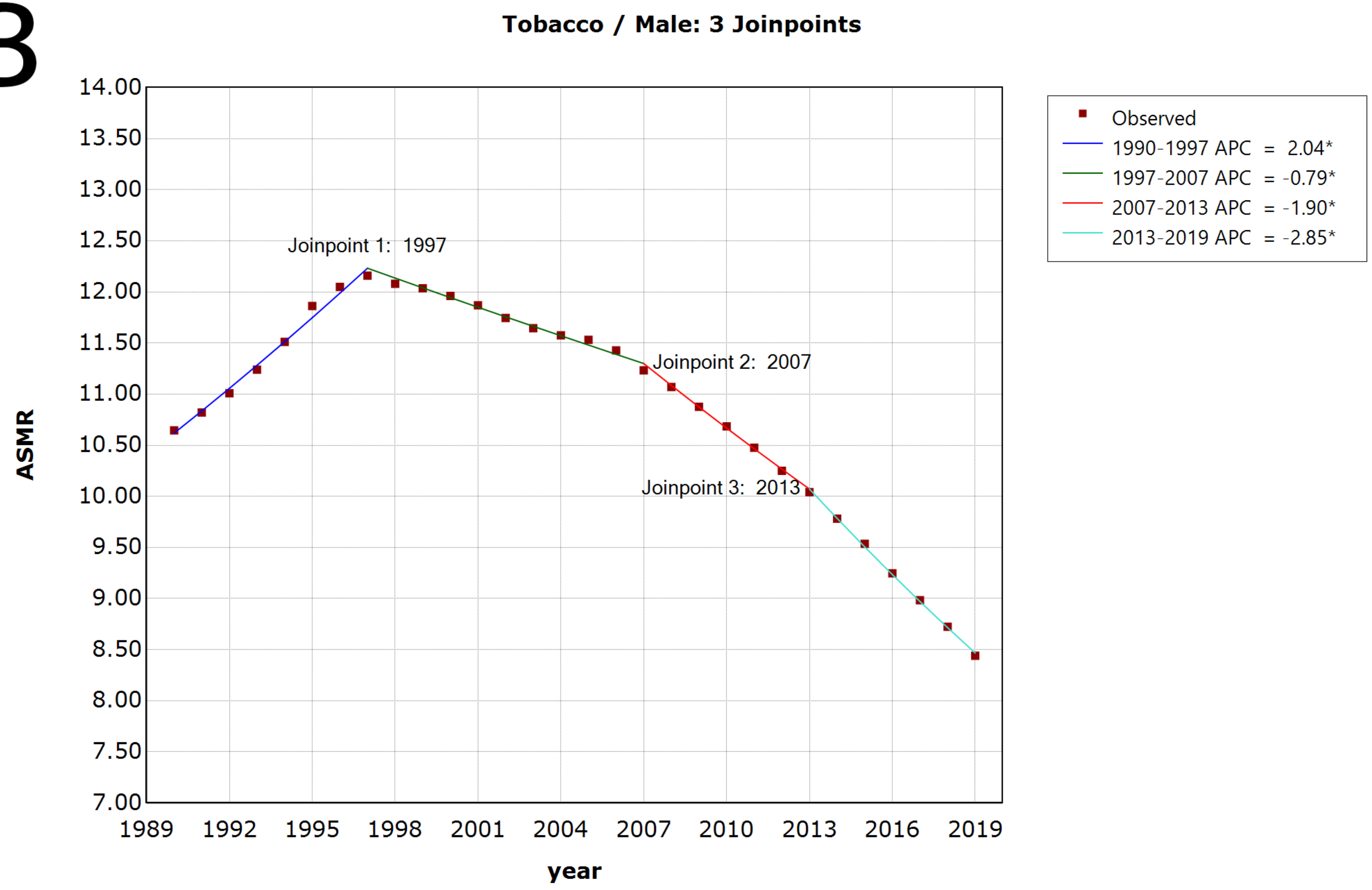

* Indicates that the Annual Percent Change (APC) is significantly different from zero at the alpha = 0.05 level
Final Selected Model: 3 Joinpoints.

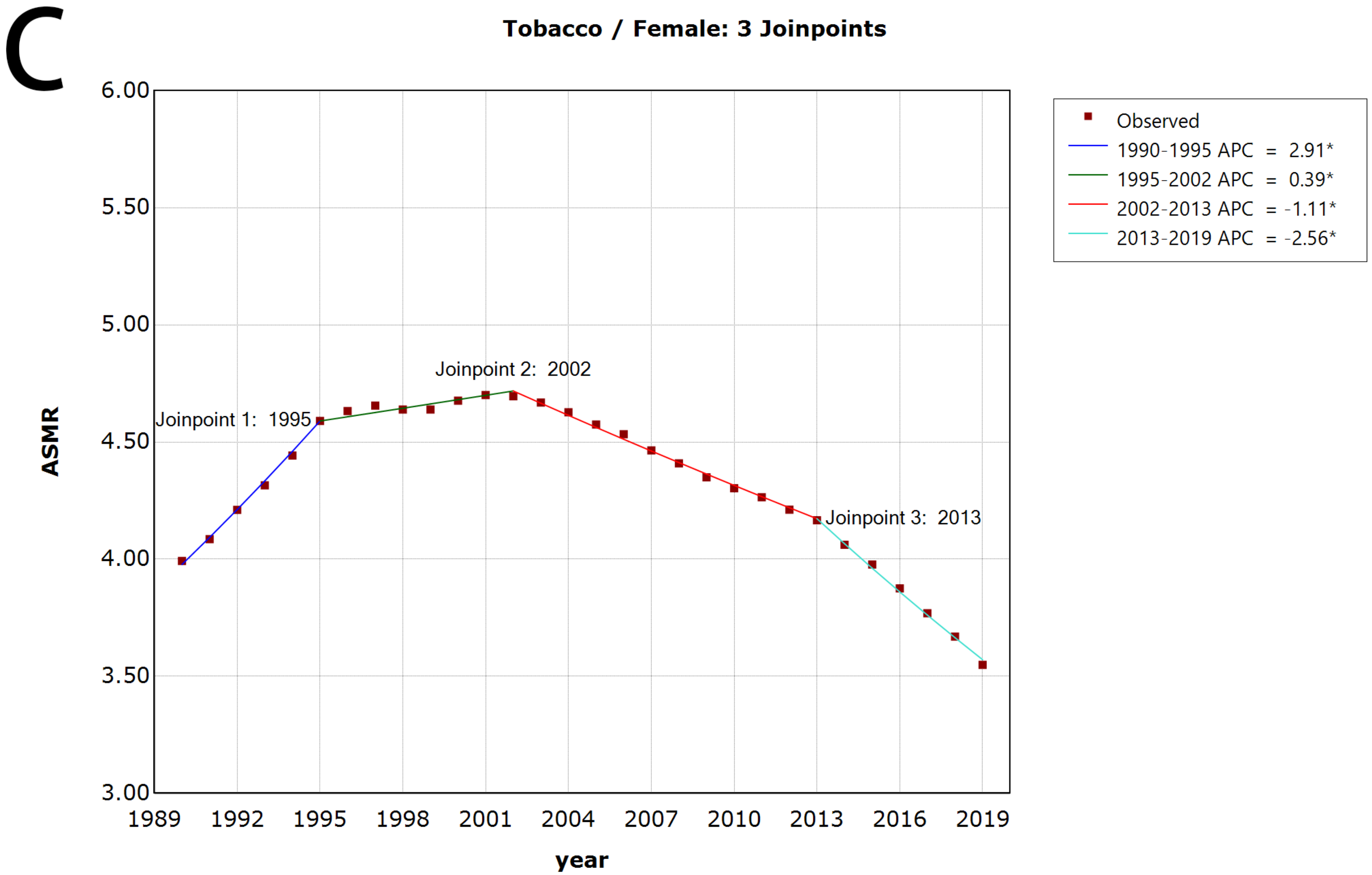

* Indicates that the Annual Percent Change (APC) is significantly different from zero at the alpha = 0.05 level
Final Selected Model: 3 Joinpoints.

D

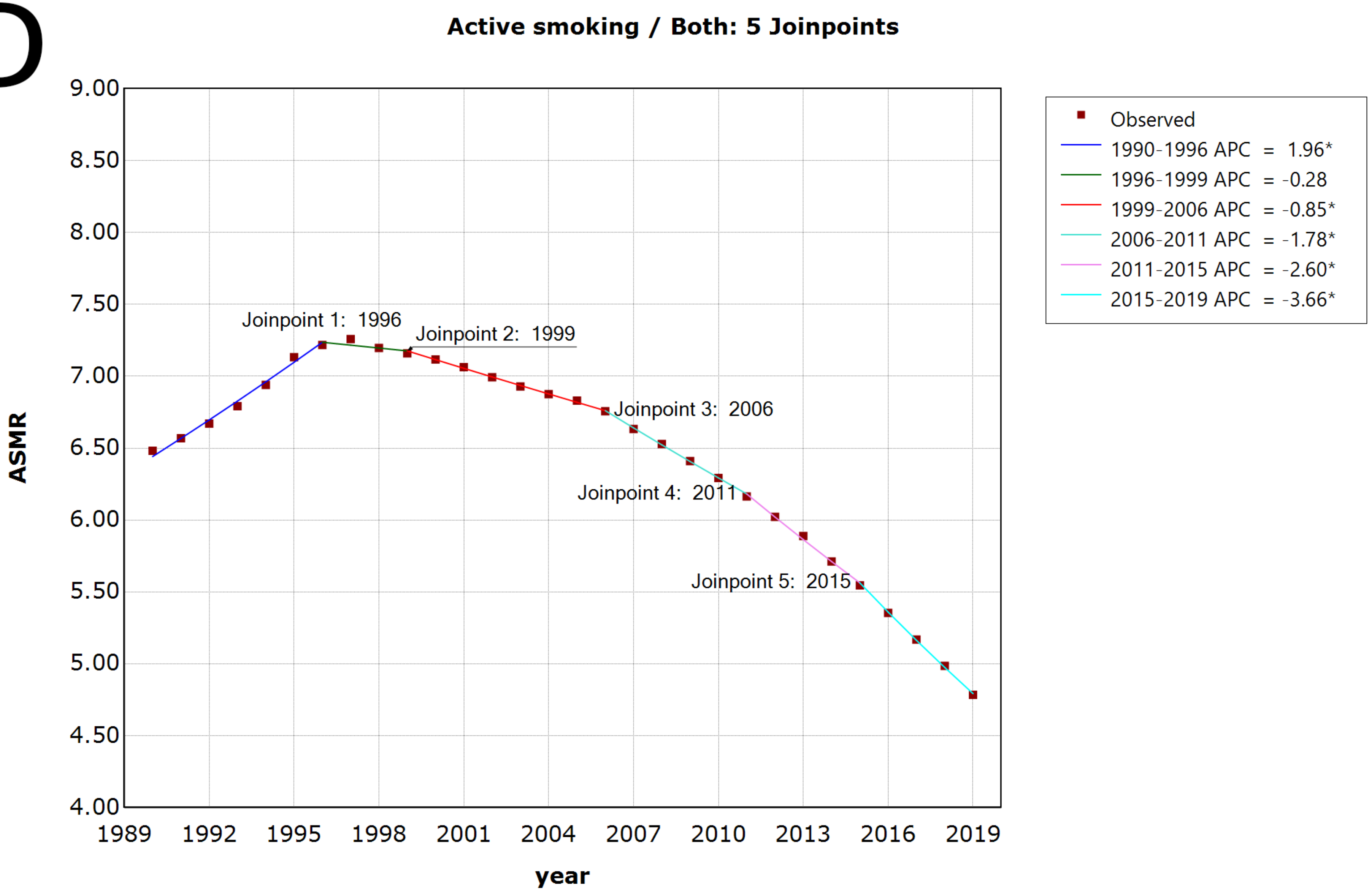

* Indicates that the Annual Percent Change (APC) is significantly different from zero at the alpha = 0.05 level
Final Selected Model: 5 Joinpoints.

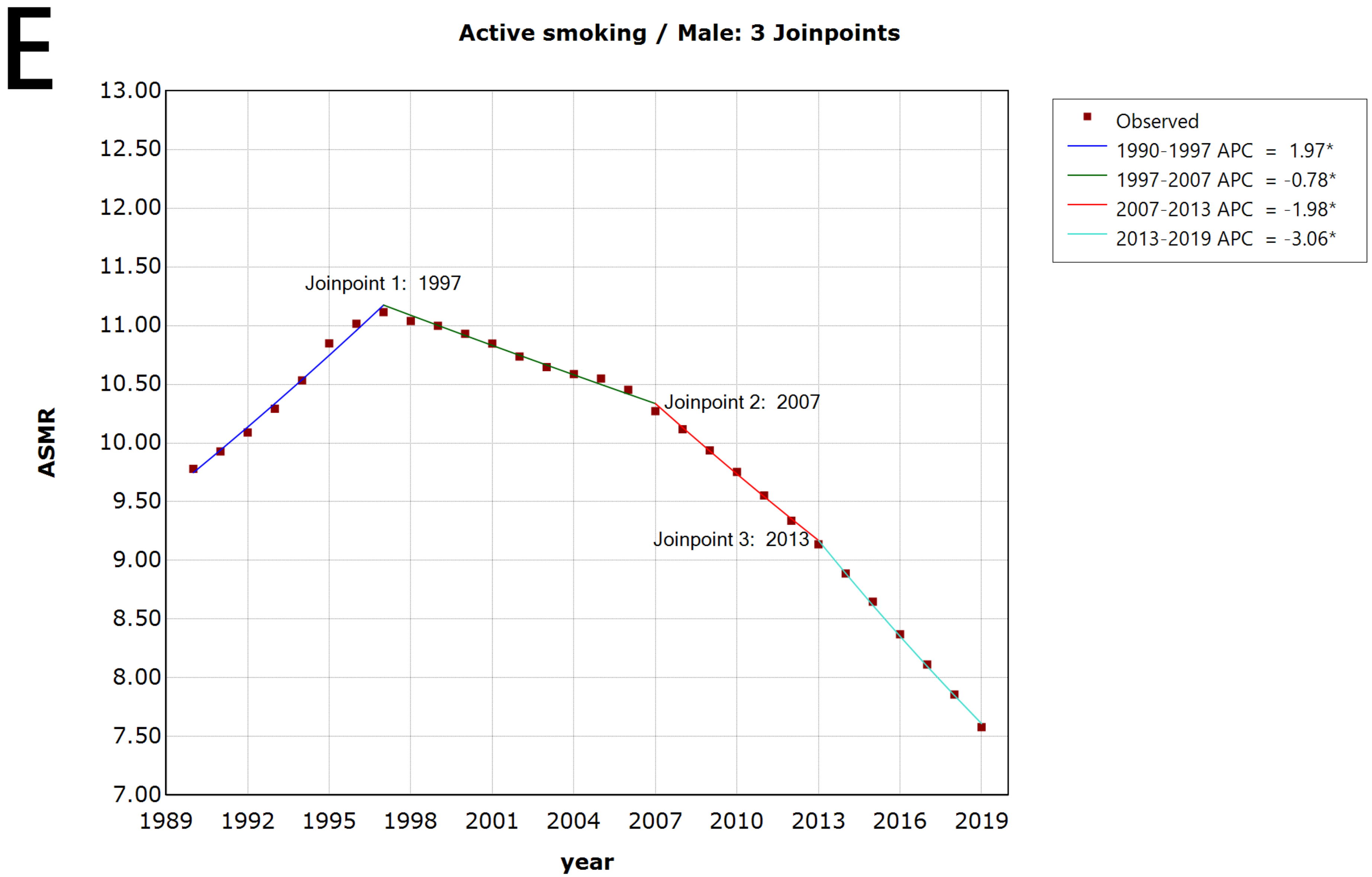

* Indicates that the Annual Percent Change (APC) is significantly different from zero at the alpha = 0.05 level
Final Selected Model: 3 Joinpoints.

F

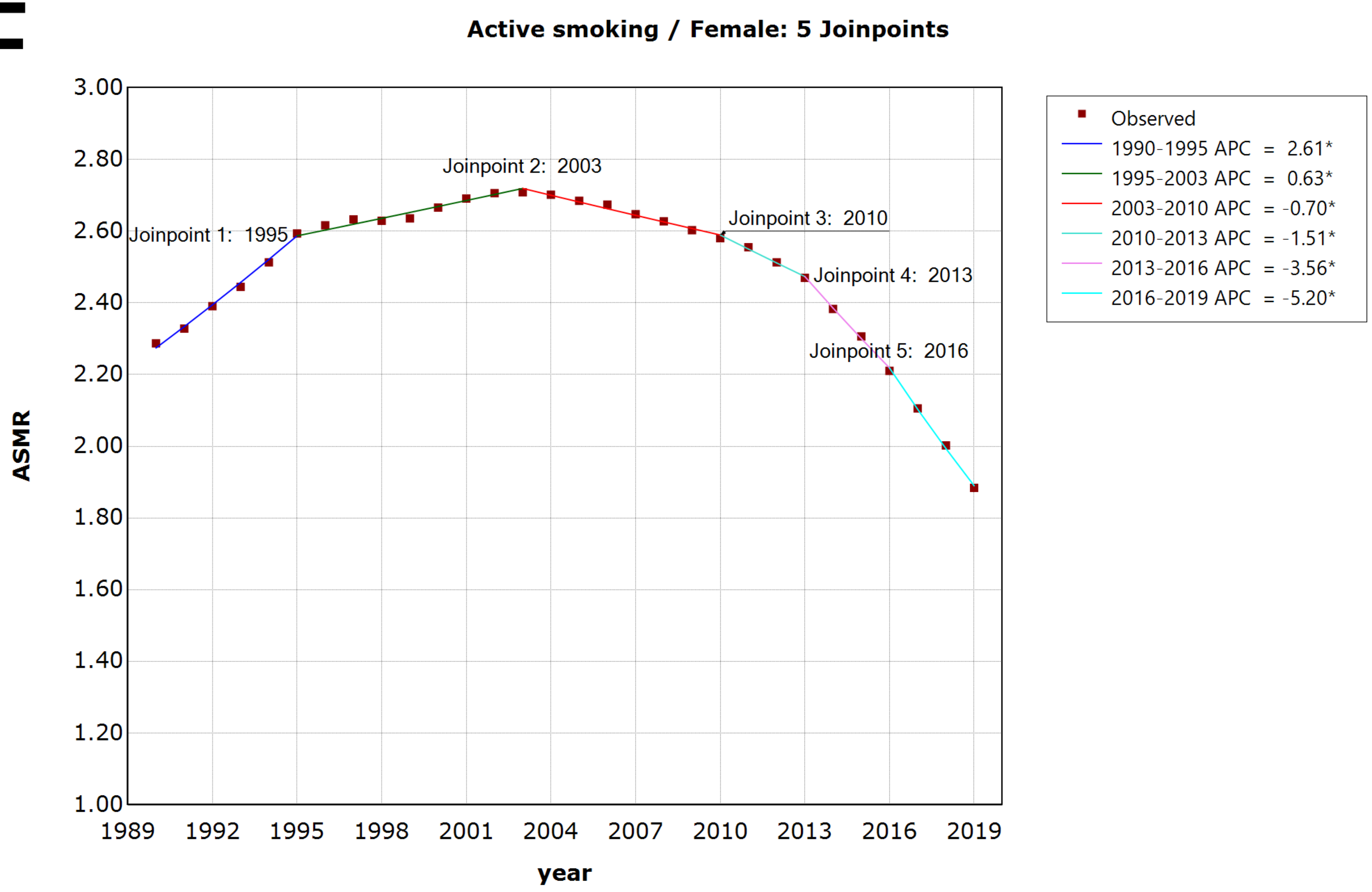

* Indicates that the Annual Percent Change (APC) is significantly different from zero at the alpha = 0.05 level
Final Selected Model: 5 Joinpoints.

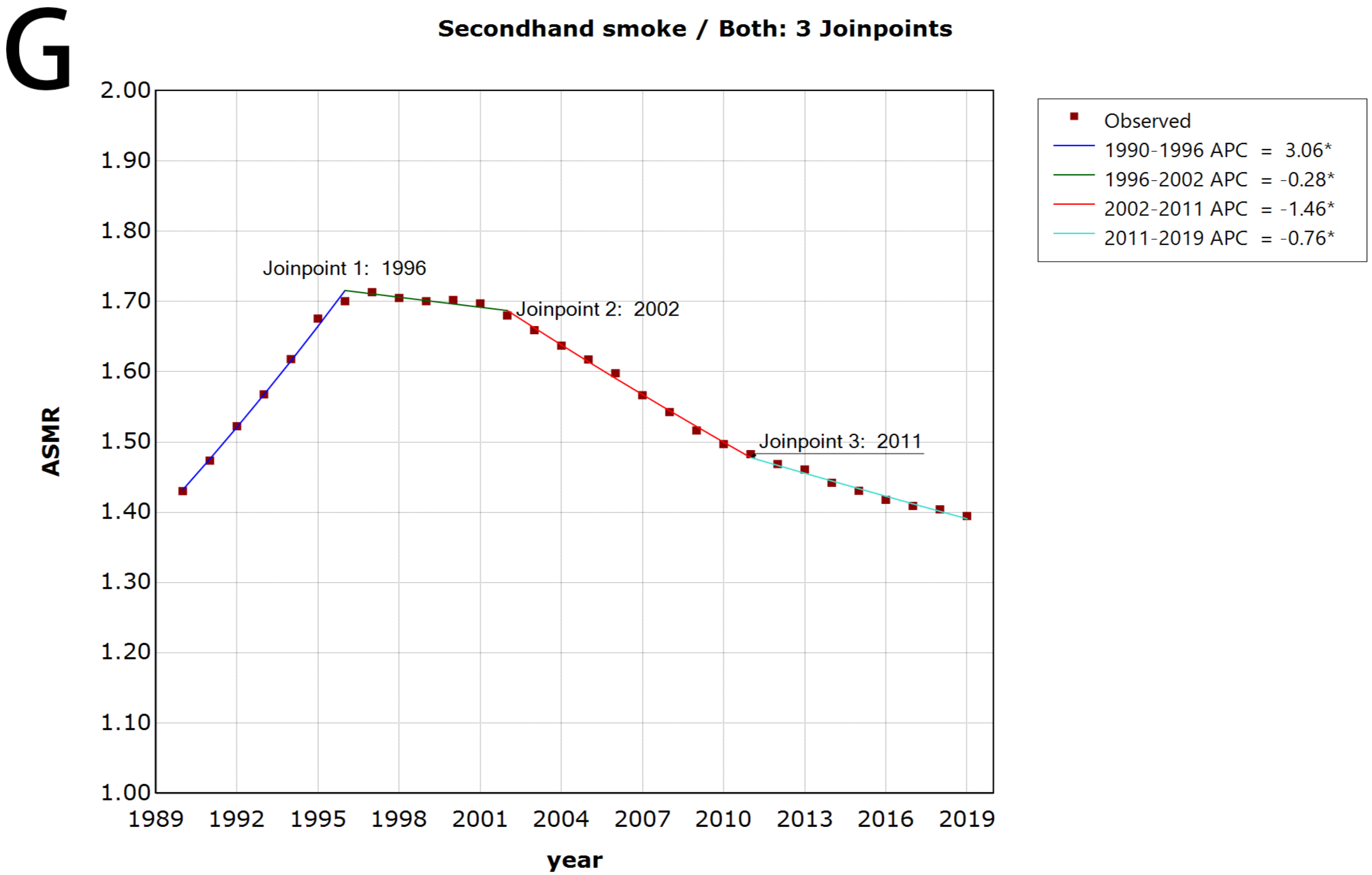

* Indicates that the Annual Percent Change (APC) is significantly different from zero at the alpha = 0.05 level
Final Selected Model: 3 Joinpoints.

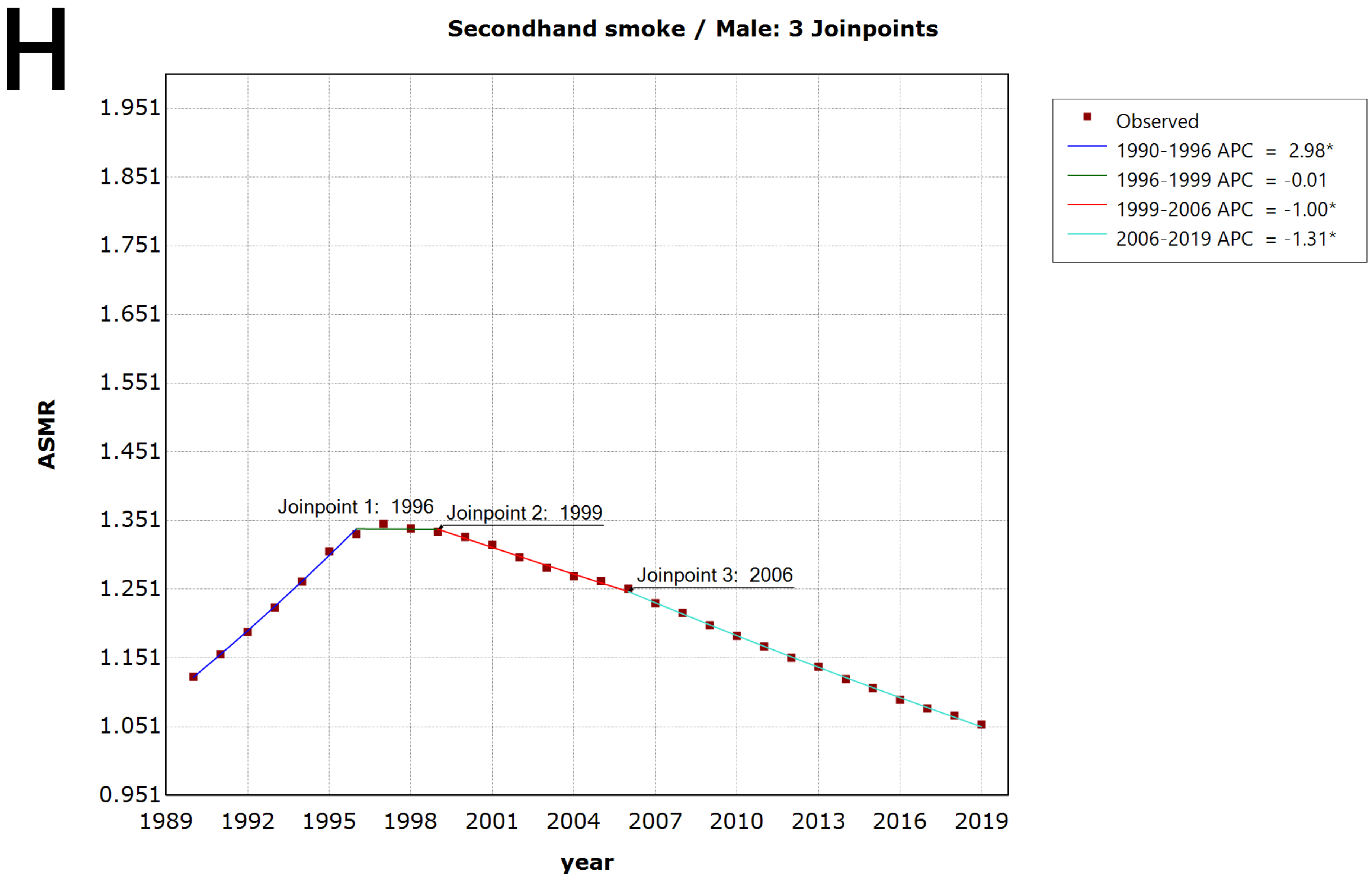

* Indicates that the Annual Percent Change (APC) is significantly different from zero at the alpha = 0.05 level
Final Selected Model: 3 Joinpoints.

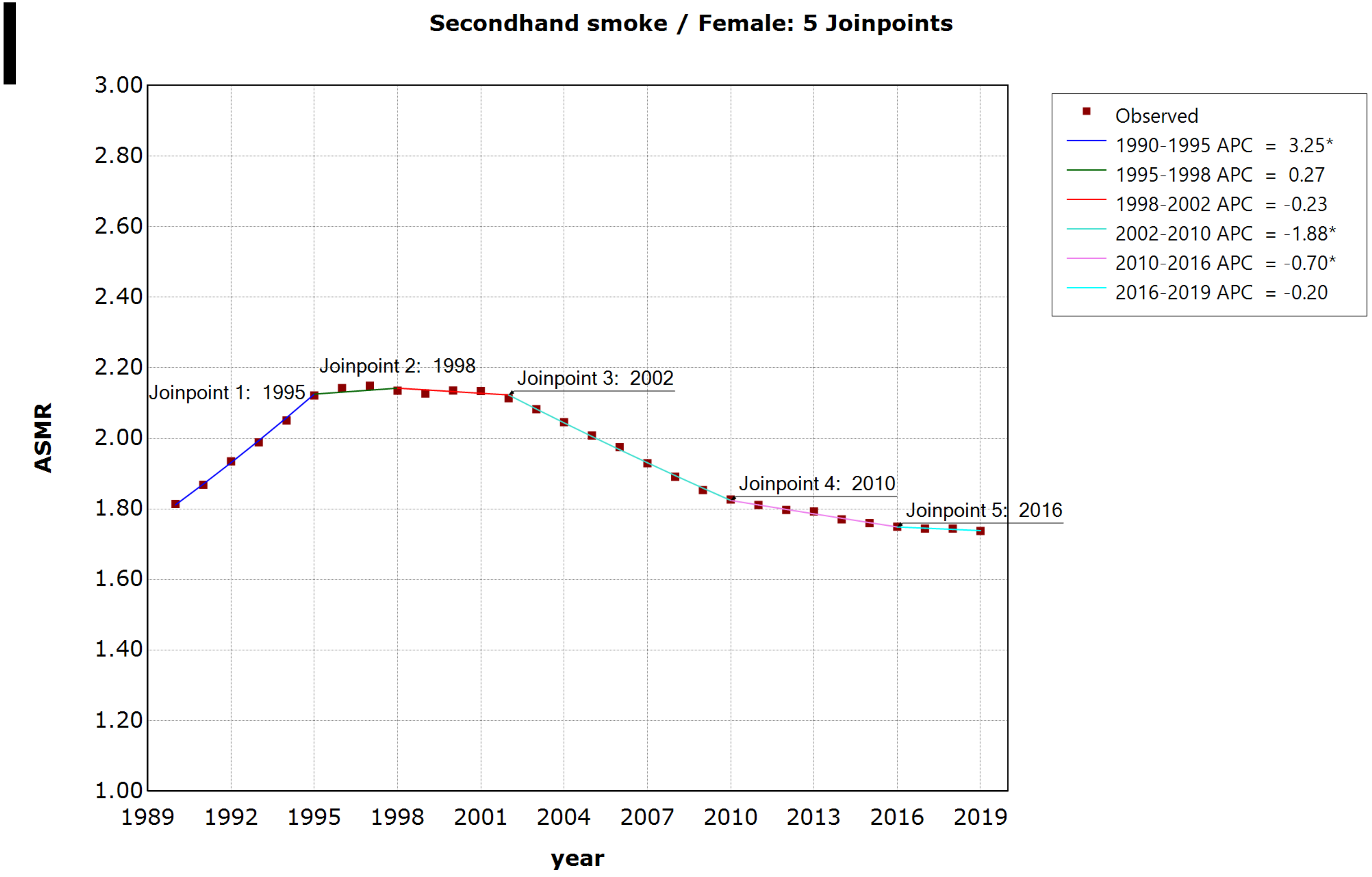

* Indicates that the Annual Percent Change (APC) is significantly different from zero at the alpha = 0.05 level
Final Selected Model: 5 Joinpoints.

Supplementary Figure 4. APC of ASMR in ischemic stroke due to tobacco use, active smoking and second-hand smoke by gender in Pakistan from 1990 to 2019.

A. APC of ASMR in ischemic stroke due to tobacco use in Pakistan from 1990 to 2019.

B. APC of ASMR in ischemic stroke due to tobacco use among men in Pakistan from 1990 to 2019.

C. APC of ASMR in ischemic stroke due to tobacco use among women in Pakistan from 1990 to 2019.

D. APC of ASMR in ischemic stroke due to active smoking in Pakistan from 1990 to 2019.

E. APC of ASMR in ischemic stroke due to active smoking among men in Pakistan from 1990 to 2019.

F. APC of ASMR in ischemic stroke due to active smoking among women in Pakistan from 1990 to 2019.

G. APC of ASMR in ischemic stroke due to secondhand smoke in Pakistan from 1990 to 2019.

H. APC of ASMR in ischemic stroke due to secondhand smoke among men in Pakistan from 1990 to 2019.

I. APC of ASMR in ischemic stroke due to secondhand smoke among women in Pakistan from 1990 to 2019.

A

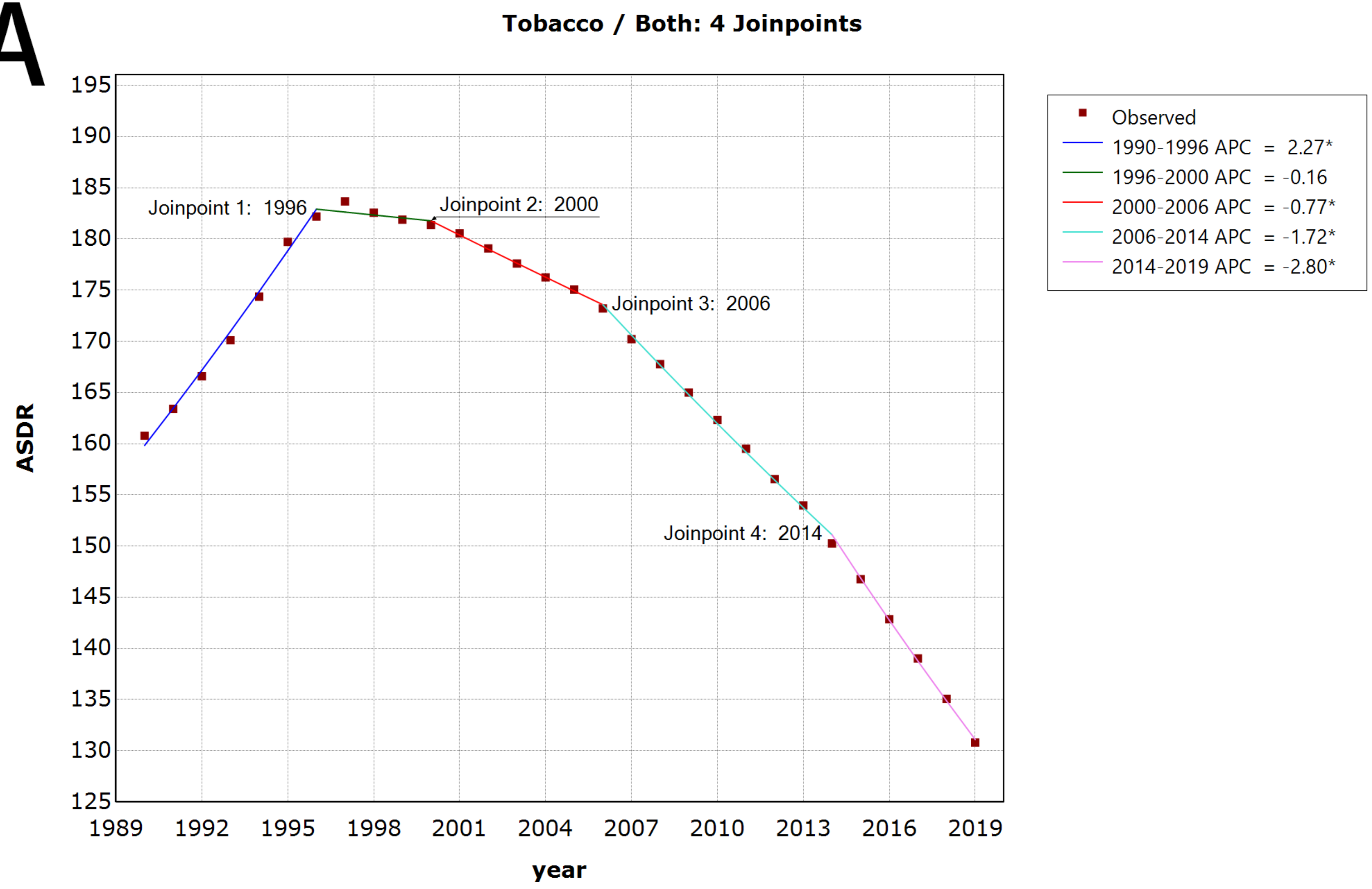

* Indicates that the Annual Percent Change (APC) is significantly different from zero at the alpha = 0.05 level
Final Selected Model: 4 Joinpoints.

B

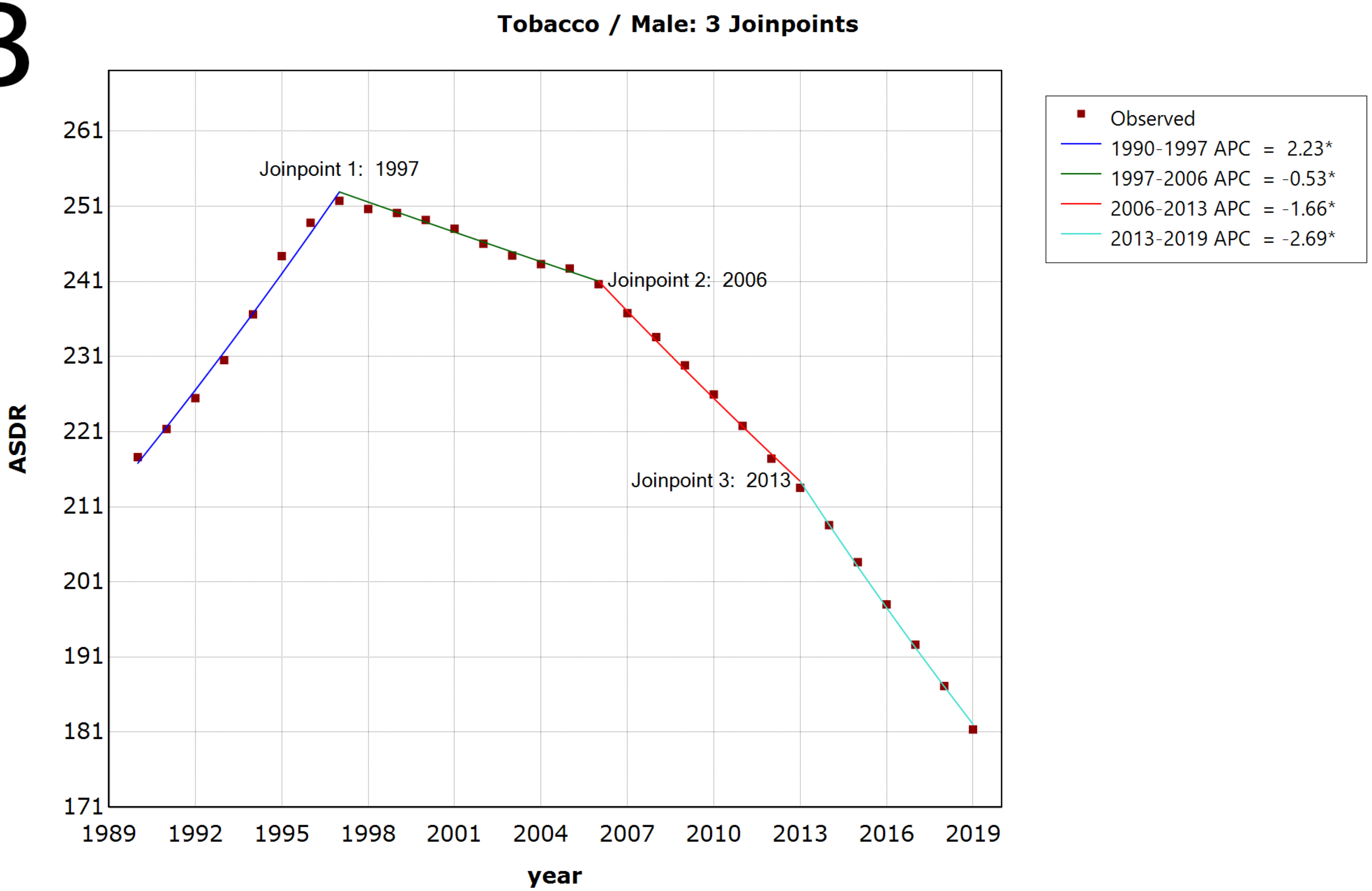

* Indicates that the Annual Percent Change (APC) is significantly different from zero at the alpha = 0.05 level
Final Selected Model: 3 Joinpoints.

C

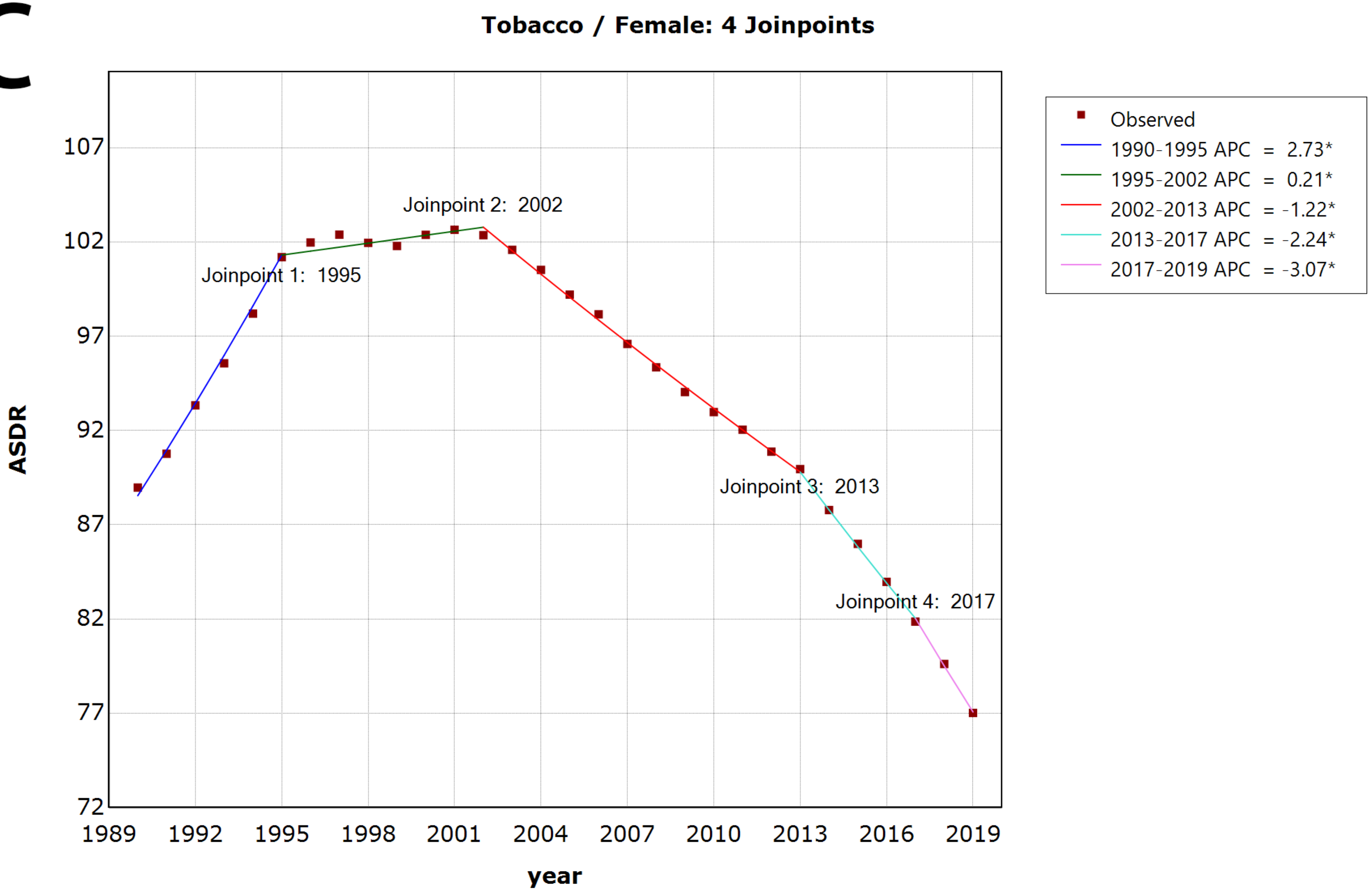

* Indicates that the Annual Percent Change (APC) is significantly different from zero at the alpha = 0.05 level
Final Selected Model: 4 Joinpoints.

D

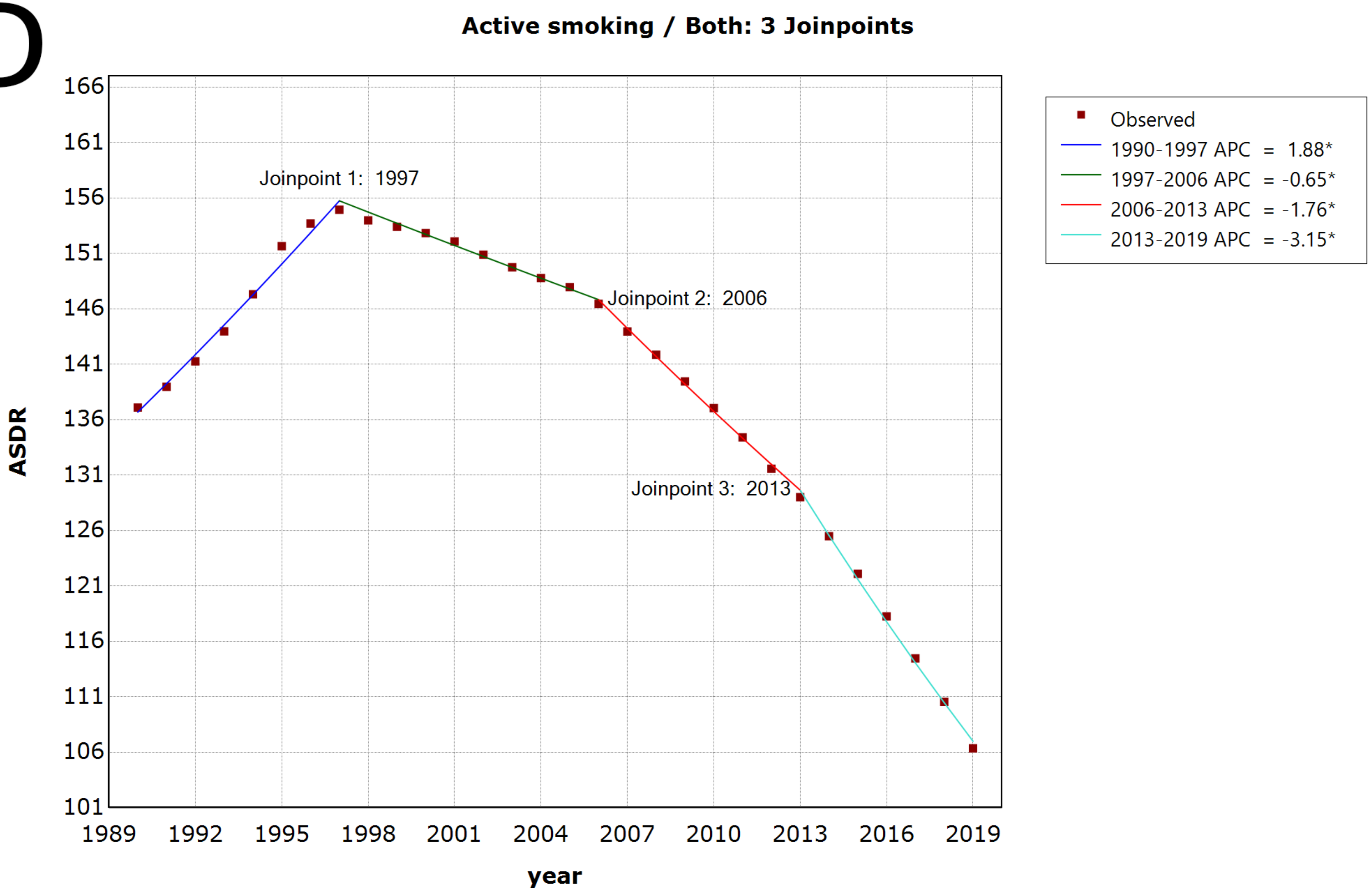

* Indicates that the Annual Percent Change (APC) is significantly different from zero at the alpha = 0.05 level
Final Selected Model: 3 Joinpoints.

E

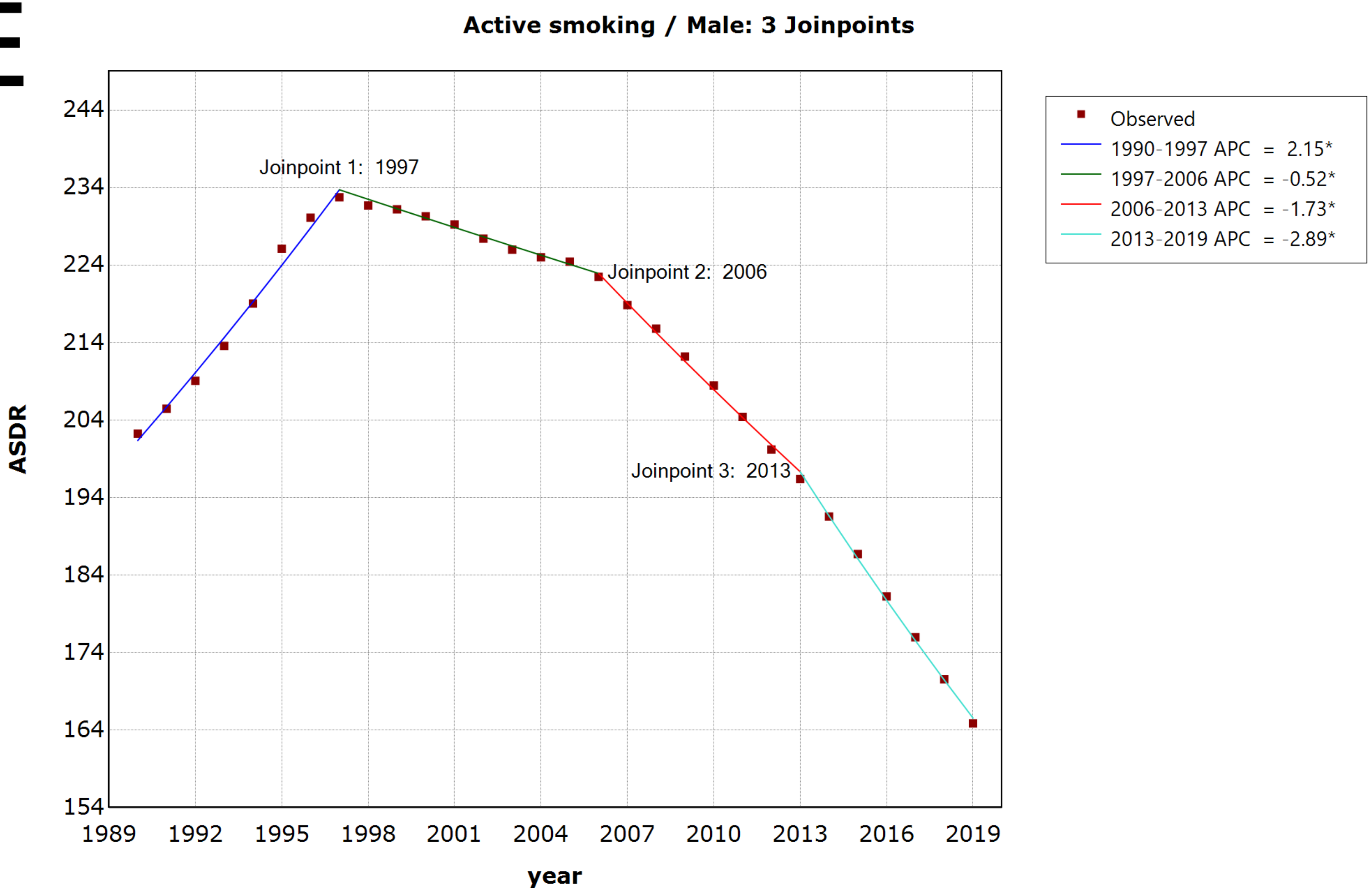

* Indicates that the Annual Percent Change (APC) is significantly different from zero at the alpha = 0.05 level
Final Selected Model: 3 Joinpoints.

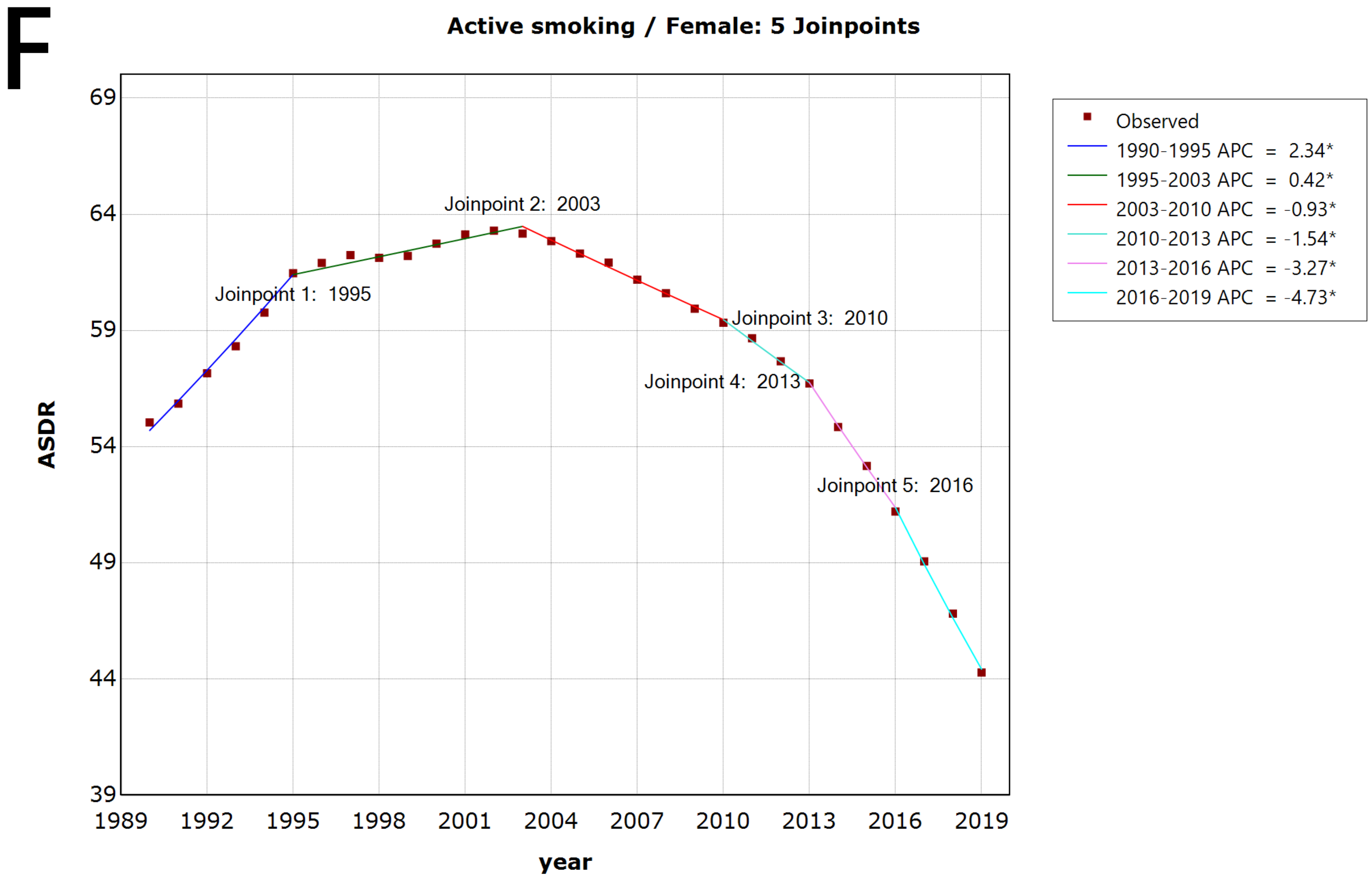

* Indicates that the Annual Percent Change (APC) is significantly different from zero at the alpha = 0.05 level
Final Selected Model: 5 Joinpoints.

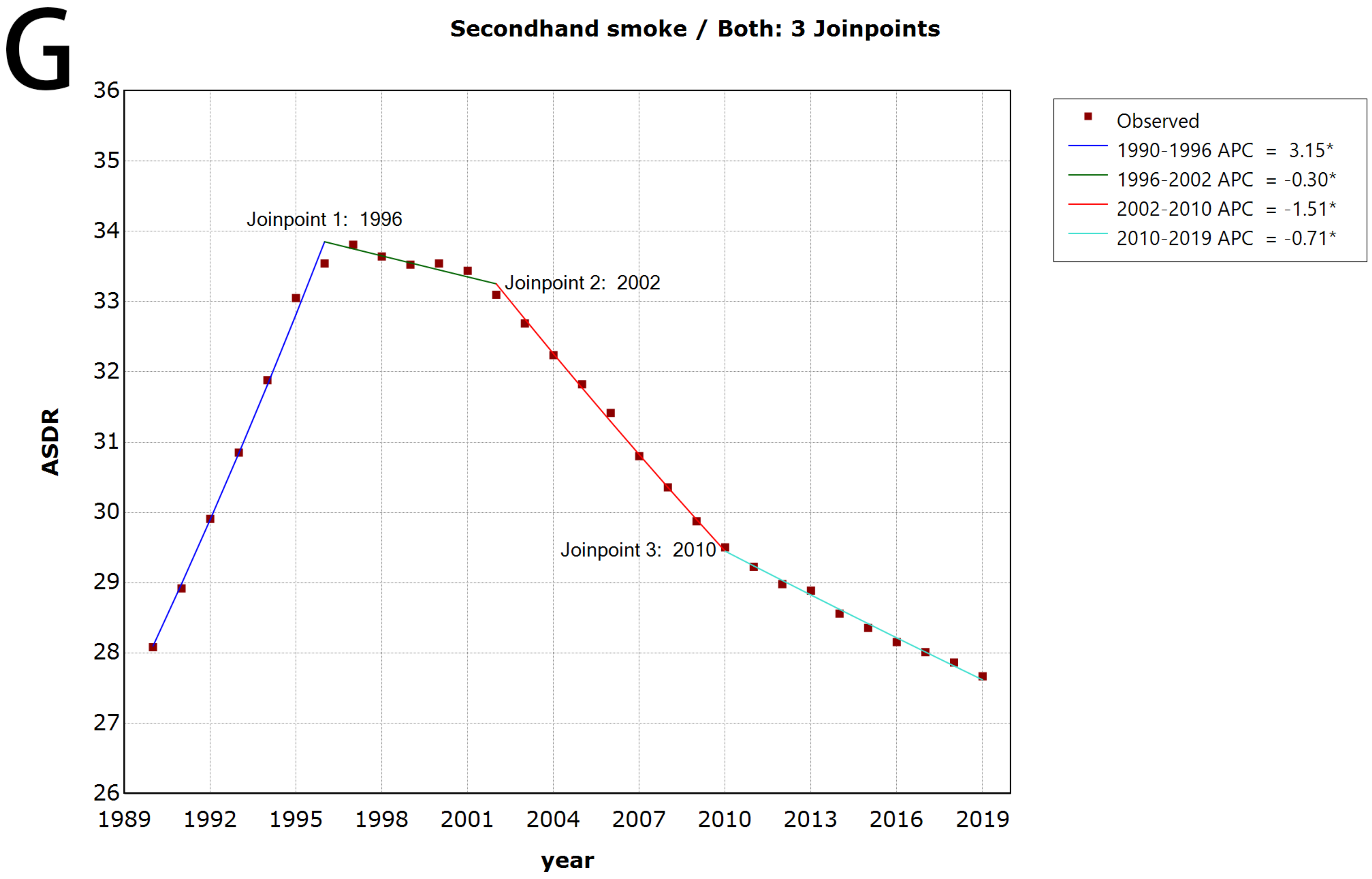

* Indicates that the Annual Percent Change (APC) is significantly different from zero at the alpha = 0.05 level
Final Selected Model: 3 Joinpoints.

H

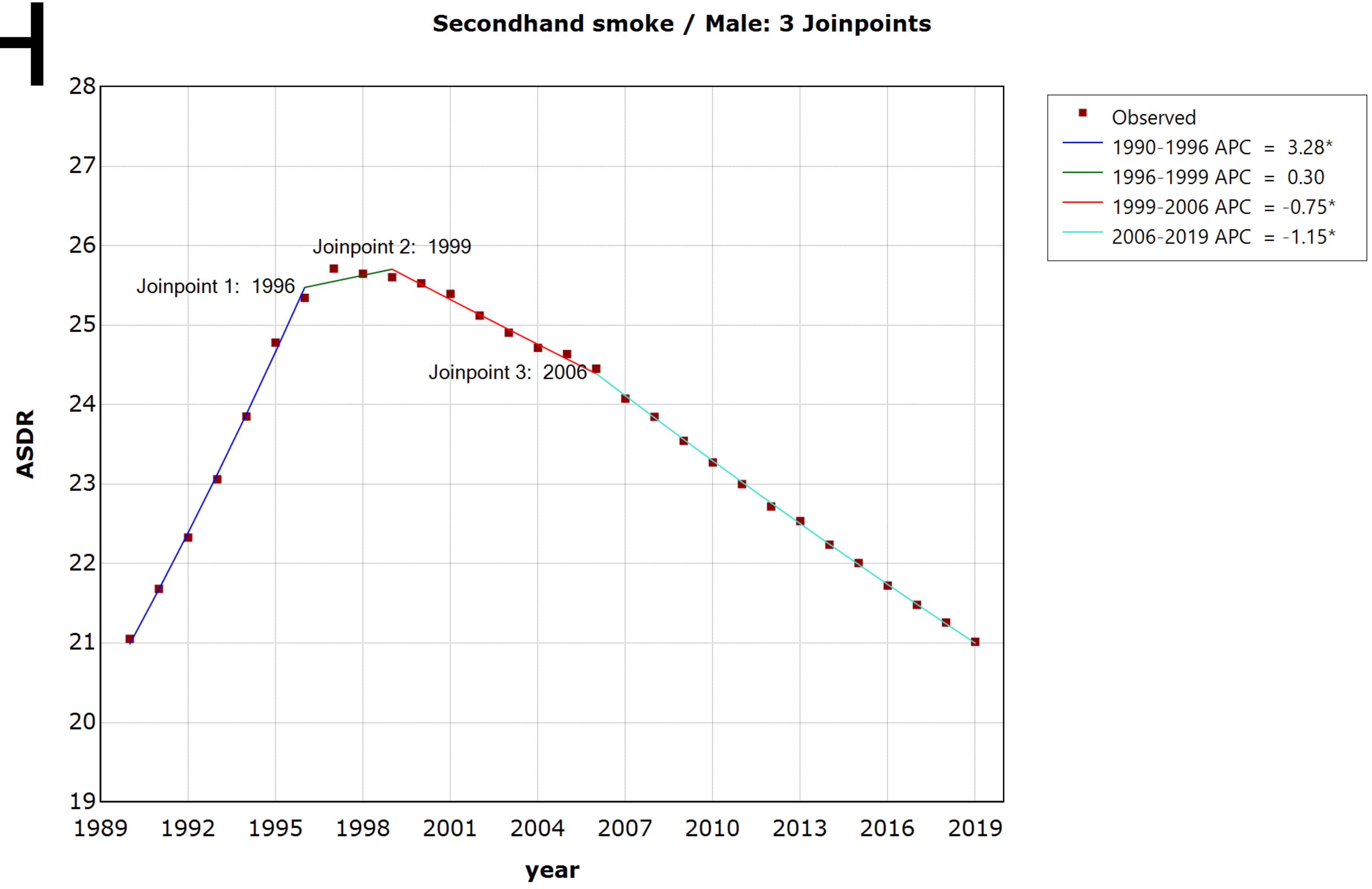

* Indicates that the Annual Percent Change (APC) is significantly different from zero at the alpha = 0.05 level
Final Selected Model: 3 Joinpoints.

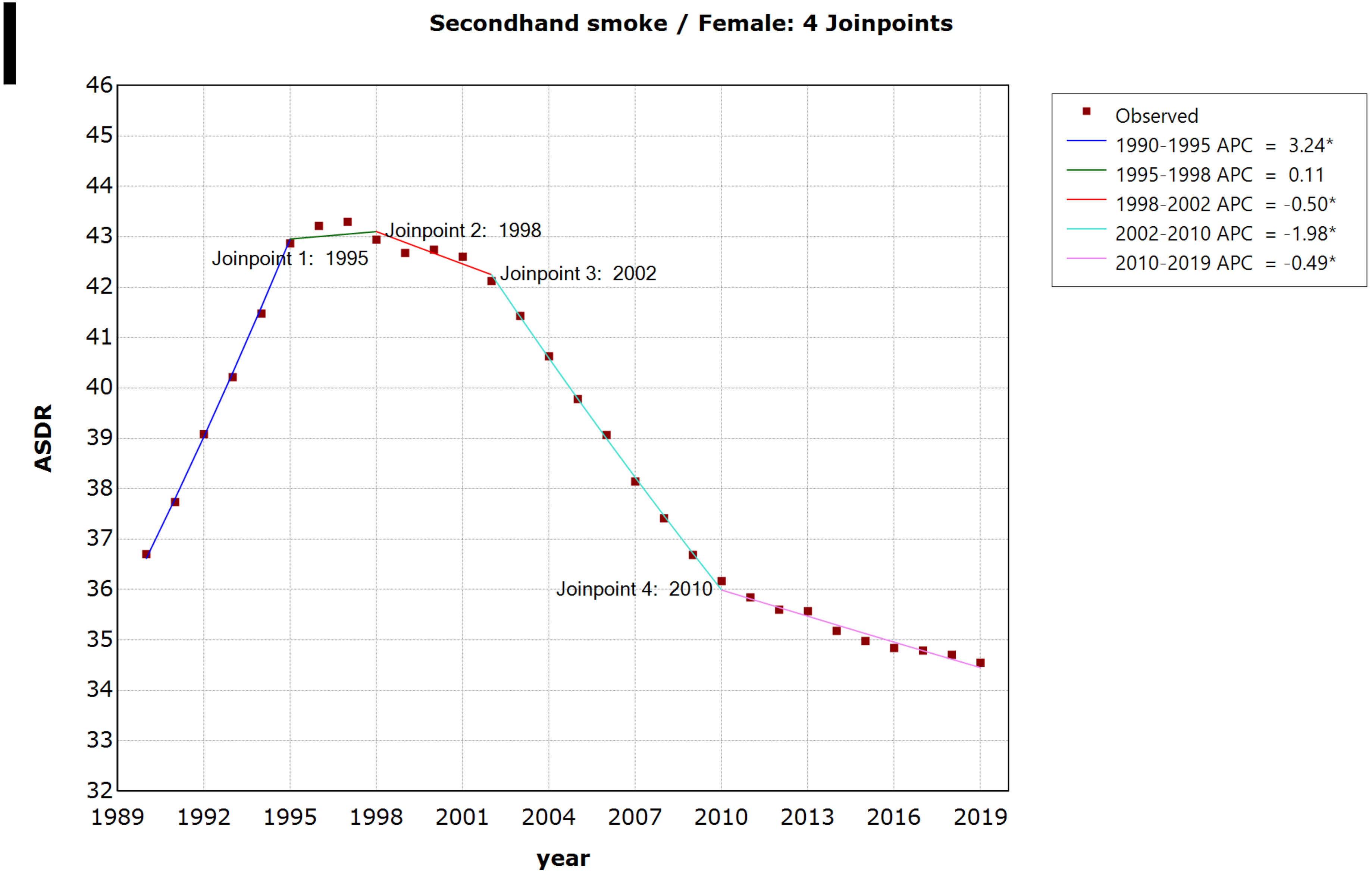

* Indicates that the Annual Percent Change (APC) is significantly different from zero at the alpha = 0.05 level
Final Selected Model: 4 Joinpoints.

Supplementary Figure 5. APC of ASDR in ischemic stroke due to tobacco use, active smoking and second-hand smoke by gender in Pakistan from 1990 to 2019.

A. APC of ASDR in ischemic stroke due to tobacco use in Pakistan from 1990 to 2019.

B. APC of ASDR in ischemic stroke due to tobacco use among men in Pakistan from 1990 to 2019.

C. APC of ASDR in ischemic stroke due to tobacco use among women in Pakistan from 1990 to 2019.

D. APC of ASDR in ischemic stroke due to active smoking in Pakistan from 1990 to 2019.

E. APC of ASDR in ischemic stroke due to active smoking among men in Pakistan from 1990 to 2019.

F. APC of ASDR in ischemic stroke due to active smoking among women in Pakistan from 1990 to 2019.

G. APC of ASDR in ischemic stroke due to secondhand smoke in Pakistan from 1990 to 2019.

H. APC of ASDR in ischemic stroke due to secondhand smoke among men in Pakistan from 1990 to 2019.

I. APC of ASDR in ischemic stroke due to secondhand smoke among women in Pakistan from 1990 to 2019.

A

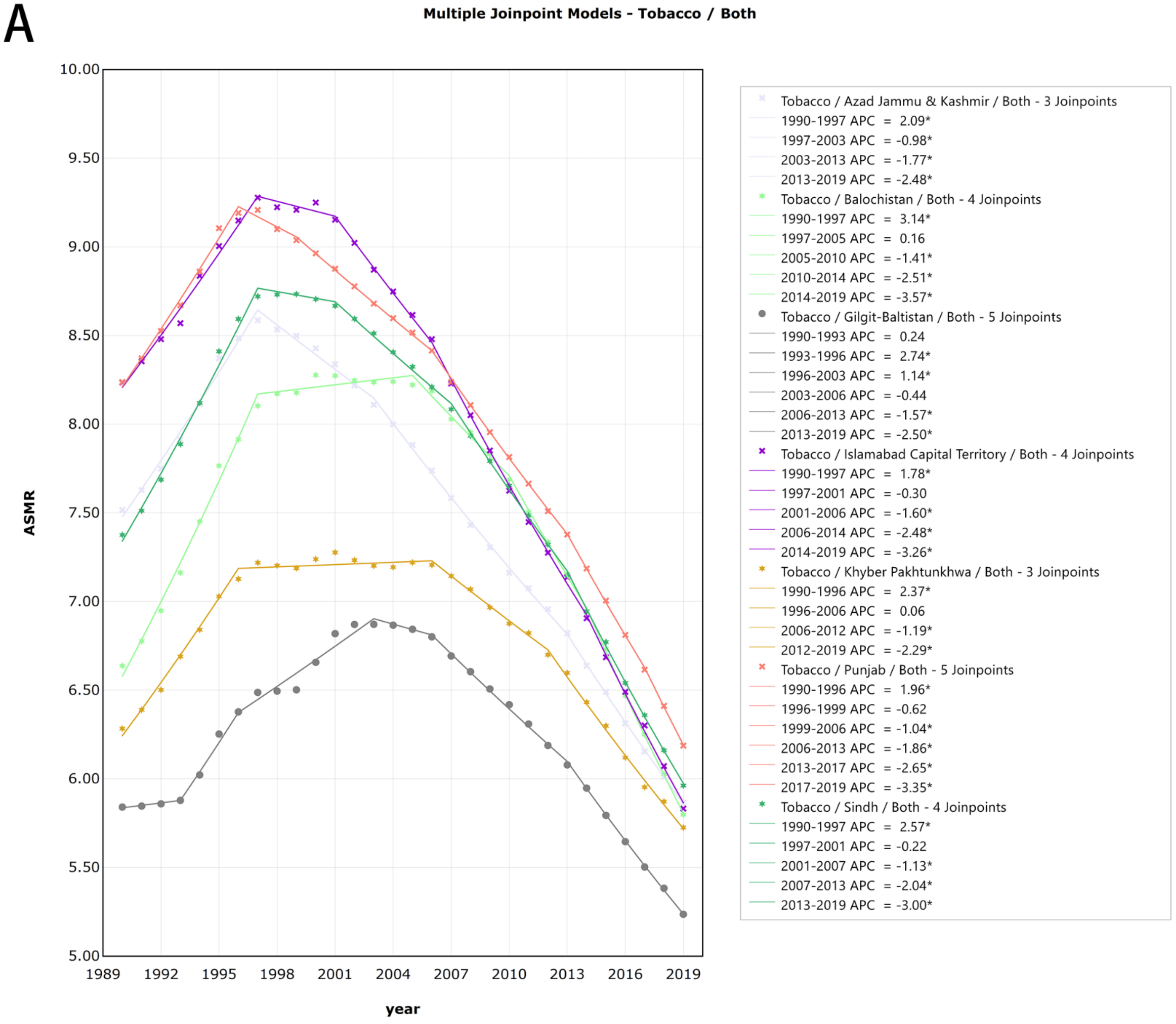
Multiple Joinpoint Models - Tobacco / Both
ASMR
year
10.00
9.50
9.00
8.50
8.00
7.50
7.00
6.50
6.00
5.50
5.00
1989
1992
1995
1998
2001
2004
2007
2010
2013
2016
2019
Tobacco / Azad Jammu & Kashmir / Both - 3 Joinpoints
1990-1997 APC = 2.09*
1997-2003 APC = -0.98*
2003-2013 APC = -1.77*
2013-2019 APC = -2.48*
Tobacco / Balochistan / Both - 4 Joinpoints
1990-1997 APC = 3.14*
1997-2005 APC = 0.16
2005-2010 APC = -1.41*
2010-2014 APC = -2.51*
2014-2019 APC = -3.57*
Tobacco / Gilgit-Baltistan / Both - 5 Joinpoints
1990-1993 APC = 0.24
1993-1996 APC = 2.74*
1996-2003 APC = 1.14*
2003-2006 APC = -0.44
2006-2013 APC = -1.57*
2013-2019 APC = -2.50*
Tobacco / Islamabad Capital Territory / Both - 4 Joinpoints
1990-1997 APC = 1.78*
1997-2001 APC = -0.30
2001-2006 APC = -1.60*
2006-2014 APC = -2.48*
2014-2019 APC = -3.26*
Tobacco / Khyber Pakhtunkhwa / Both - 3 Joinpoints
1990-1996 APC = 2.37*
1996-2006 APC = 0.06
2006-2012 APC = -1.19*
2012-2019 APC = -2.29*
Tobacco / Punjab / Both - 5 Joinpoints
1990-1996 APC = 1.96*
1996-1999 APC = -0.62
1999-2006 APC = -1.04*
2006-2013 APC = -1.86*
2013-2017 APC = -2.65*
2017-2019 APC = -3.35*
Tobacco / Sindh / Both - 4 Joinpoints
1990-1997 APC = 2.57*
1997-2001 APC = -0.22
2001-2007 APC = -1.13*
2007-2013 APC = -2.04*
2013-2019 APC = -3.00*

B

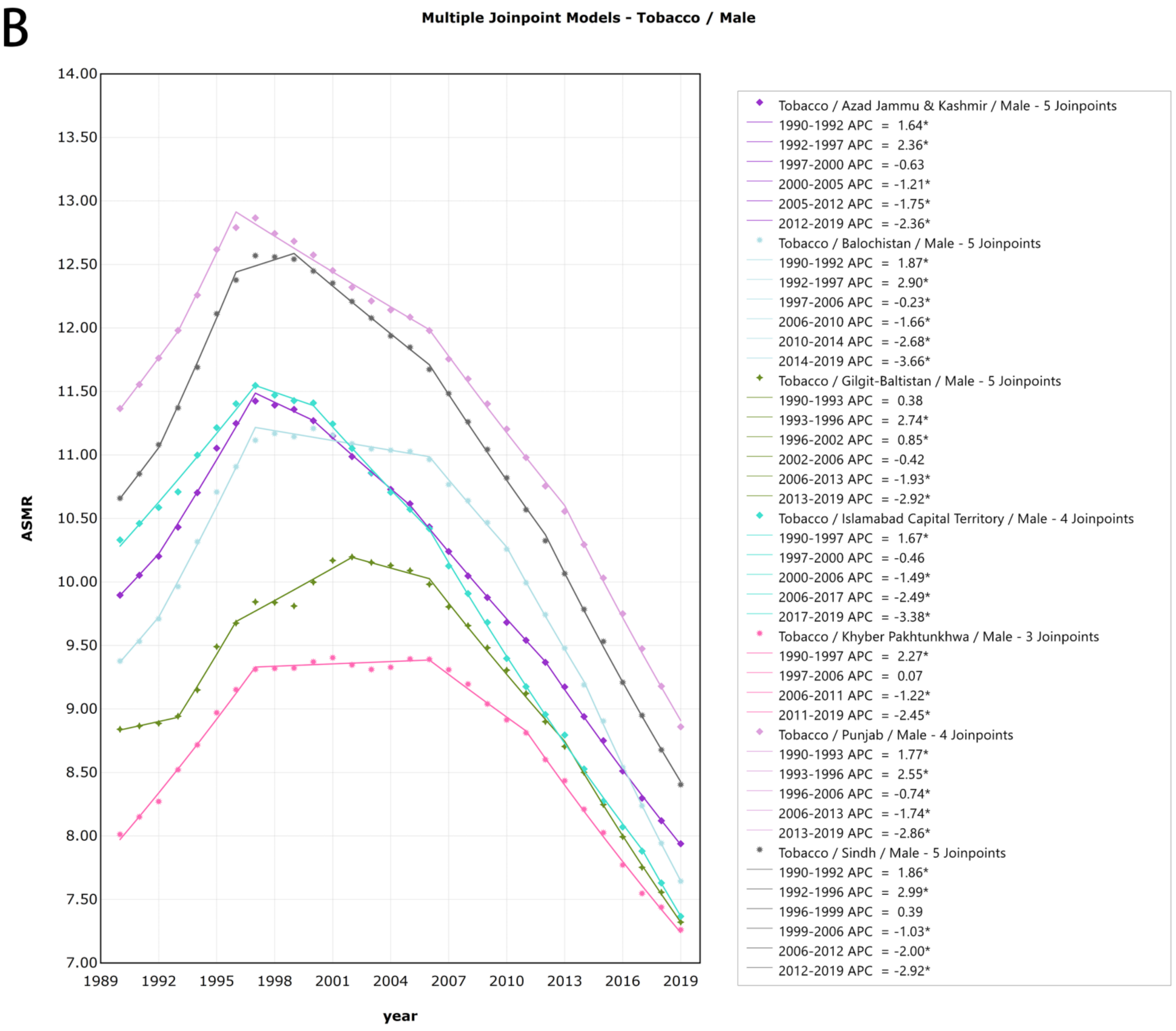

Multiple Joinpoint Models - Tobacco / Male
ASMR
year
14.00
13.50
13.00
12.50
12.00
11.50
11.00
10.50
10.00
9.50
9.00
8.50
8.00
7.50
7.00
1989
1992
1995
1998
2001
2004
2007
2010
2013
2016
2019
Tobacco / Azad Jammu & Kashmir / Male - 5 Joinpoints
1990-1992 APC = 1.64*
1992-1997 APC = 2.36*
1997-2000 APC = -0.63
2000-2005 APC = -1.21*
2005-2012 APC = -1.75*
2012-2019 APC = -2.36*
Tobacco / Balochistan / Male - 5 Joinpoints
1990-1992 APC = 1.87*
1992-1997 APC = 2.90*
1997-2006 APC = -0.23*
2006-2010 APC = -1.66*
2010-2014 APC = -2.68*
2014-2019 APC = -3.66*
Tobacco / Gilgit-Baltistan / Male - 5 Joinpoints
1990-1993 APC = 0.38
1993-1996 APC = 2.74*
1996-2002 APC = 0.85*
2002-2006 APC = -0.42
2006-2013 APC = -1.93*
2013-2019 APC = -2.92*
Tobacco / Islamabad Capital Territory / Male - 4 Joinpoints
1990-1997 APC = 1.67*
1997-2000 APC = -0.46
2000-2006 APC = -1.49*
2006-2017 APC = -2.49*
2017-2019 APC = -3.38*
Tobacco / Khyber Pakhtunkhwa / Male - 3 Joinpoints
1990-1997 APC = 2.27*
1997-2006 APC = 0.07
2006-2011 APC = -1.22*
2011-2019 APC = -2.45*
Tobacco / Punjab / Male - 4 Joinpoints
1990-1993 APC = 1.77*
1993-1996 APC = 2.55*
1996-2006 APC = -0.74*
2006-2013 APC = -1.74*
2013-2019 APC = -2.86*
Tobacco / Sindh / Male - 5 Joinpoints
1990-1992 APC = 1.86*
1992-1996 APC = 2.99*
1996-1999 APC = 0.39
1999-2006 APC = -1.03*
2006-2012 APC = -2.00*
2012-2019 APC = -2.92*

C

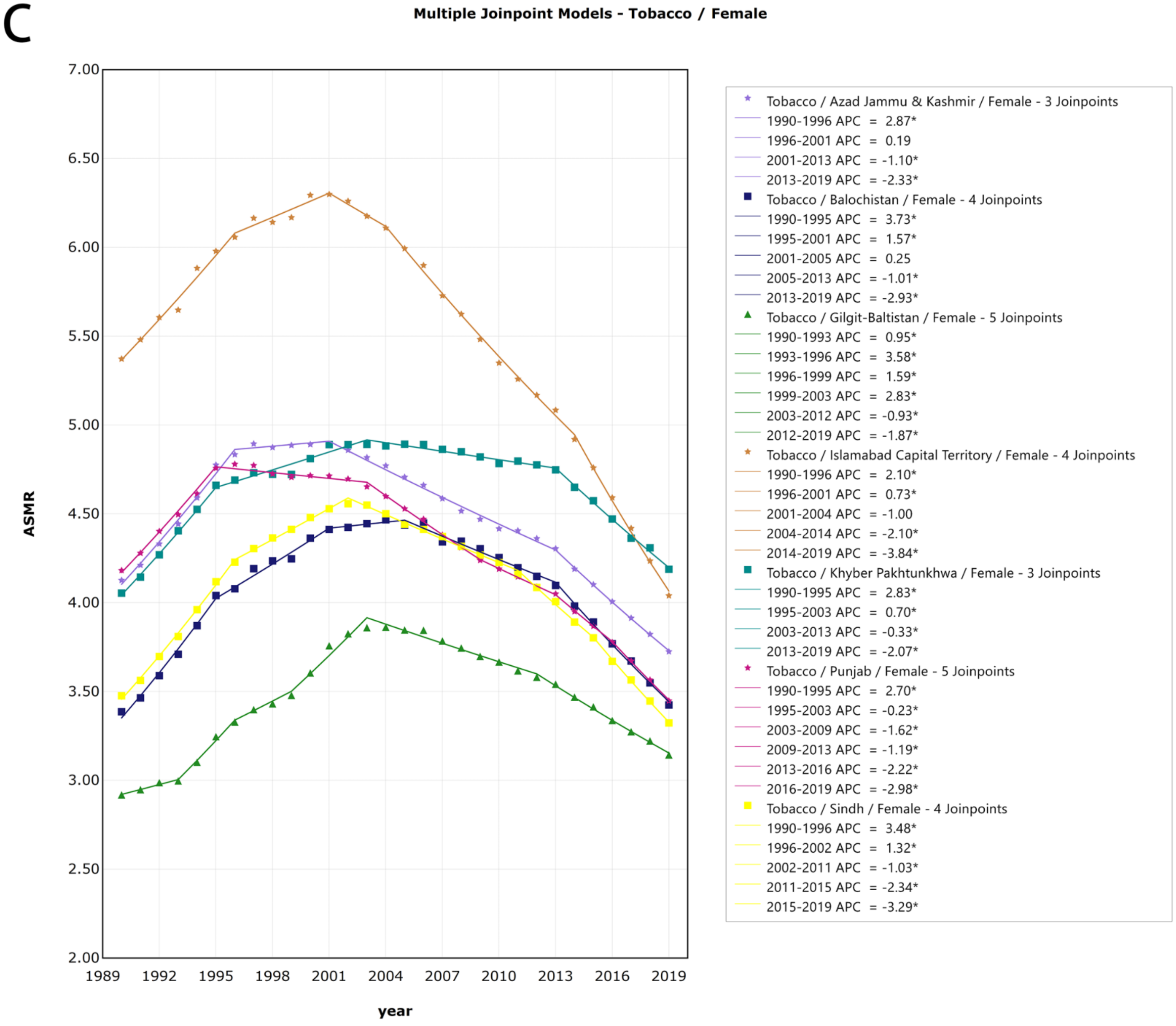

Multiple Joinpoint Models - Tobacco / Female
ASMR
year
7.00
6.50
6.00
5.50
5.00
4.50
4.00
3.50
3.00
2.50
2.00
1989
1992
1995
1998
2001
2004
2007
2010
2013
2016
2019
Tobacco / Azad Jammu & Kashmir / Female - 3 Joinpoints
1990-1996 APC = 2.87*
1996-2001 APC = 0.19
2001-2013 APC = -1.10*
2013-2019 APC = -2.33*
Tobacco / Balochistan / Female - 4 Joinpoints
1990-1995 APC = 3.73*
1995-2001 APC = 1.57*
2001-2005 APC = 0.25
2005-2013 APC = -1.01*
2013-2019 APC = -2.93*
Tobacco / Gilgit-Baltistan / Female - 5 Joinpoints
1990-1993 APC = 0.95*
1993-1996 APC = 3.58*
1996-1999 APC = 1.59*
1999-2003 APC = 2.83*
2003-2012 APC = -0.93*
2012-2019 APC = -1.87*
Tobacco / Islamabad Capital Territory / Female - 4 Joinpoints
1990-1996 APC = 2.10*
1996-2001 APC = 0.73*
2001-2004 APC = -1.00
2004-2014 APC = -2.10*
2014-2019 APC = -3.84*
Tobacco / Khyber Pakhtunkhwa / Female - 3 Joinpoints
1990-1995 APC = 2.83*
1995-2003 APC = 0.70*
2003-2013 APC = -0.33*
2013-2019 APC = -2.07*
Tobacco / Punjab / Female - 5 Joinpoints
1990-1995 APC = 2.70*
1995-2003 APC = -0.23*
2003-2009 APC = -1.62*
2009-2013 APC = -1.19*
2013-2016 APC = -2.22*
2016-2019 APC = -2.98*
Tobacco / Sindh / Female - 4 Joinpoints
1990-1996 APC = 3.48*
1996-2002 APC = 1.32*
2002-2011 APC = -1.03*
2011-2015 APC = -2.34*
2015-2019 APC = -3.29*

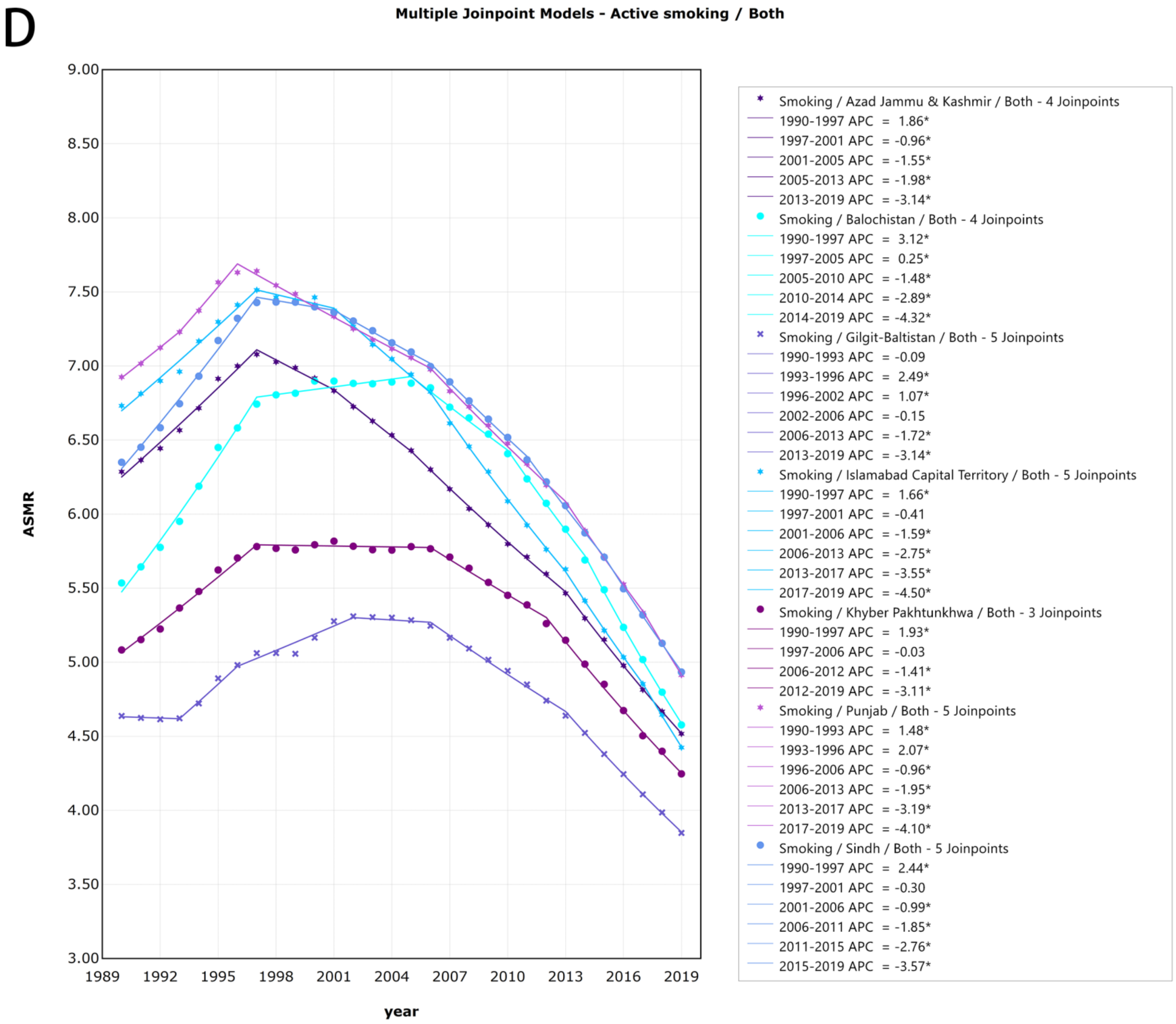
D
Multiple Joinpoint Models - Active smoking / Both
ASMR
year
9.00
8.50
8.00
7.50
7.00
6.50
6.00
5.50
5.00
4.50
4.00
3.50
3.00
1989
1992
1995
1998
2001
2004
2007
2010
2013
2016
2019
Smoking / Azad Jammu & Kashmir / Both - 4 Joinpoints
1990-1997 APC = 1.86*
1997-2001 APC = -0.96*
2001-2005 APC = -1.55*
2005-2013 APC = -1.98*
2013-2019 APC = -3.14*
Smoking / Balochistan / Both - 4 Joinpoints
1990-1997 APC = 3.12*
1997-2005 APC = 0.25*
2005-2010 APC = -1.48*
2010-2014 APC = -2.89*
2014-2019 APC = -4.32*
Smoking / Gilgit-Baltistan / Both - 5 Joinpoints
1990-1993 APC = -0.09
1993-1996 APC = 2.49*
1996-2002 APC = 1.07*
2002-2006 APC = -0.15
2006-2013 APC = -1.72*
2013-2019 APC = -3.14*
Smoking / Islamabad Capital Territory / Both - 5 Joinpoints
1990-1997 APC = 1.66*
1997-2001 APC = -0.41
2001-2006 APC = -1.59*
2006-2013 APC = -2.75*
2013-2017 APC = -3.55*
2017-2019 APC = -4.50*
Smoking / Khyber Pakhtunkhwa / Both - 3 Joinpoints
1990-1997 APC = 1.93*
1997-2006 APC = -0.03
2006-2012 APC = -1.41*
2012-2019 APC = -3.11*
Smoking / Punjab / Both - 5 Joinpoints
1990-1993 APC = 1.48*
1993-1996 APC = 2.07*
1996-2006 APC = -0.96*
2006-2013 APC = -1.95*
2013-2017 APC = -3.19*
2017-2019 APC = -4.10*
Smoking / Sindh / Both - 5 Joinpoints
1990-1997 APC = 2.44*
1997-2001 APC = -0.30
2001-2006 APC = -0.99*
2006-2011 APC = -1.85*
2011-2015 APC = -2.76*
2015-2019 APC = -3.57*

E

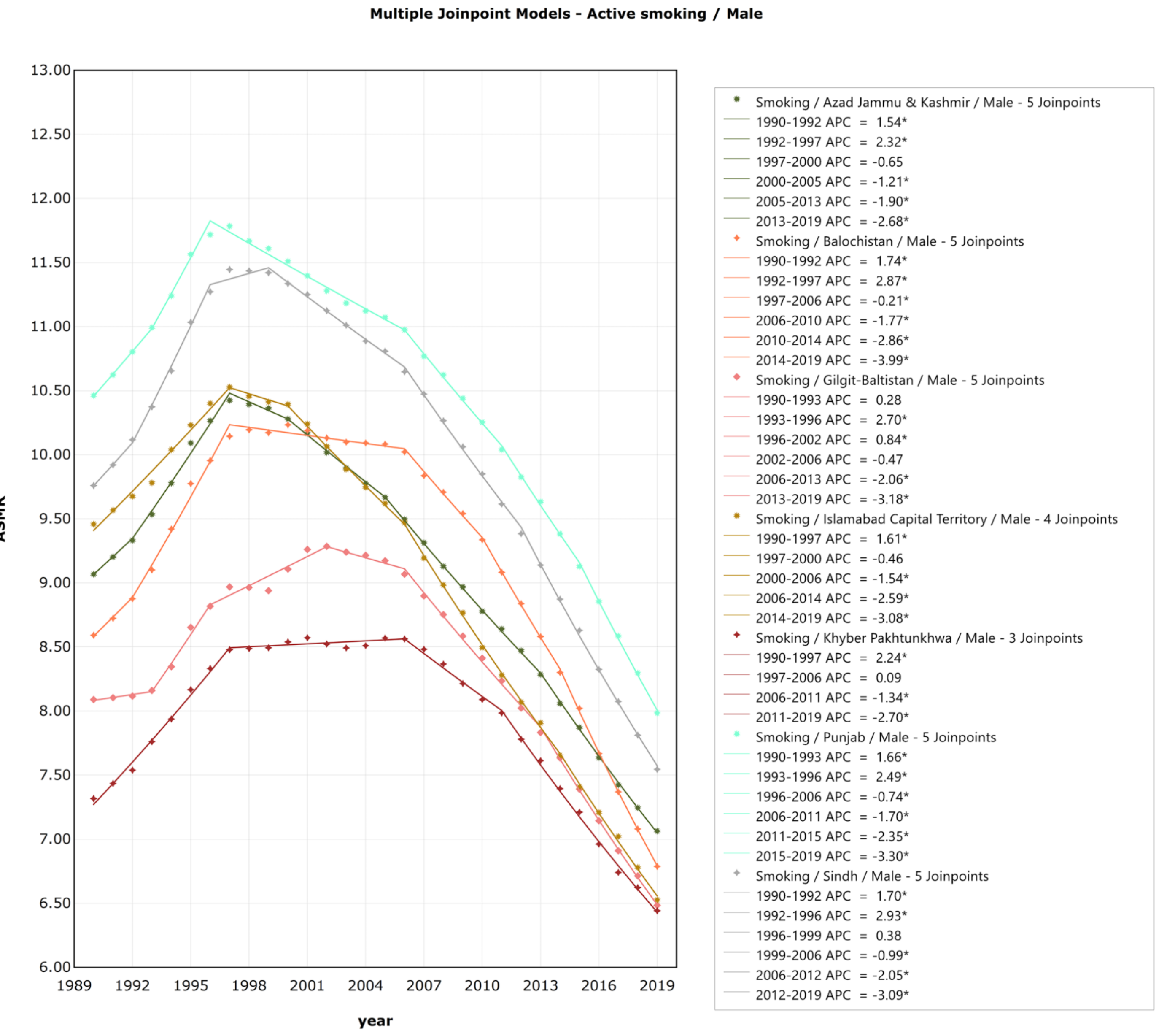

Multiple Joinpoint Models - Active smoking / Male
ASMR
year
13.00
12.50
12.00
11.50
11.00
10.50
10.00
9.50
9.00
8.50
8.00
7.50
7.00
6.50
6.00
1989
1992
1995
1998
2001
2004
2007
2010
2013
2016
2019
Smoking / Azad Jammu & Kashmir / Male - 5 Joinpoints
1990-1992 APC = 1.54*
1992-1997 APC = 2.32*
1997-2000 APC = -0.65
2000-2005 APC = -1.21*
2005-2013 APC = -1.90*
2013-2019 APC = -2.68*
Smoking / Balochistan / Male - 5 Joinpoints
1990-1992 APC = 1.74*
1992-1997 APC = 2.87*
1997-2006 APC = -0.21*
2006-2010 APC = -1.77*
2010-2014 APC = -2.86*
2014-2019 APC = -3.99*
Smoking / Gilgit-Baltistan / Male - 5 Joinpoints
1990-1993 APC = 0.28
1993-1996 APC = 2.70*
1996-2002 APC = 0.84*
2002-2006 APC = -0.47
2006-2013 APC = -2.06*
2013-2019 APC = -3.18*
Smoking / Islamabad Capital Territory / Male - 4 Joinpoints
1990-1997 APC = 1.61*
1997-2000 APC = -0.46
2000-2006 APC = -1.54*
2006-2014 APC = -2.59*
2014-2019 APC = -3.08*
Smoking / Khyber Pakhtunkhwa / Male - 3 Joinpoints
1990-1997 APC = 2.24*
1997-2006 APC = 0.09
2006-2011 APC = -1.34*
2011-2019 APC = -2.70*
Smoking / Punjab / Male - 5 Joinpoints
1990-1993 APC = 1.66*
1993-1996 APC = 2.49*
1996-2006 APC = -0.74*
2006-2011 APC = -1.70*
2011-2015 APC = -2.35*
2015-2019 APC = -3.30*
Smoking / Sindh / Male - 5 Joinpoints
1990-1992 APC = 1.70*
1992-1996 APC = 2.93*
1996-1999 APC = 0.38
1999-2006 APC = -0.99*
2006-2012 APC = -2.05*
2012-2019 APC = -3.09*

F

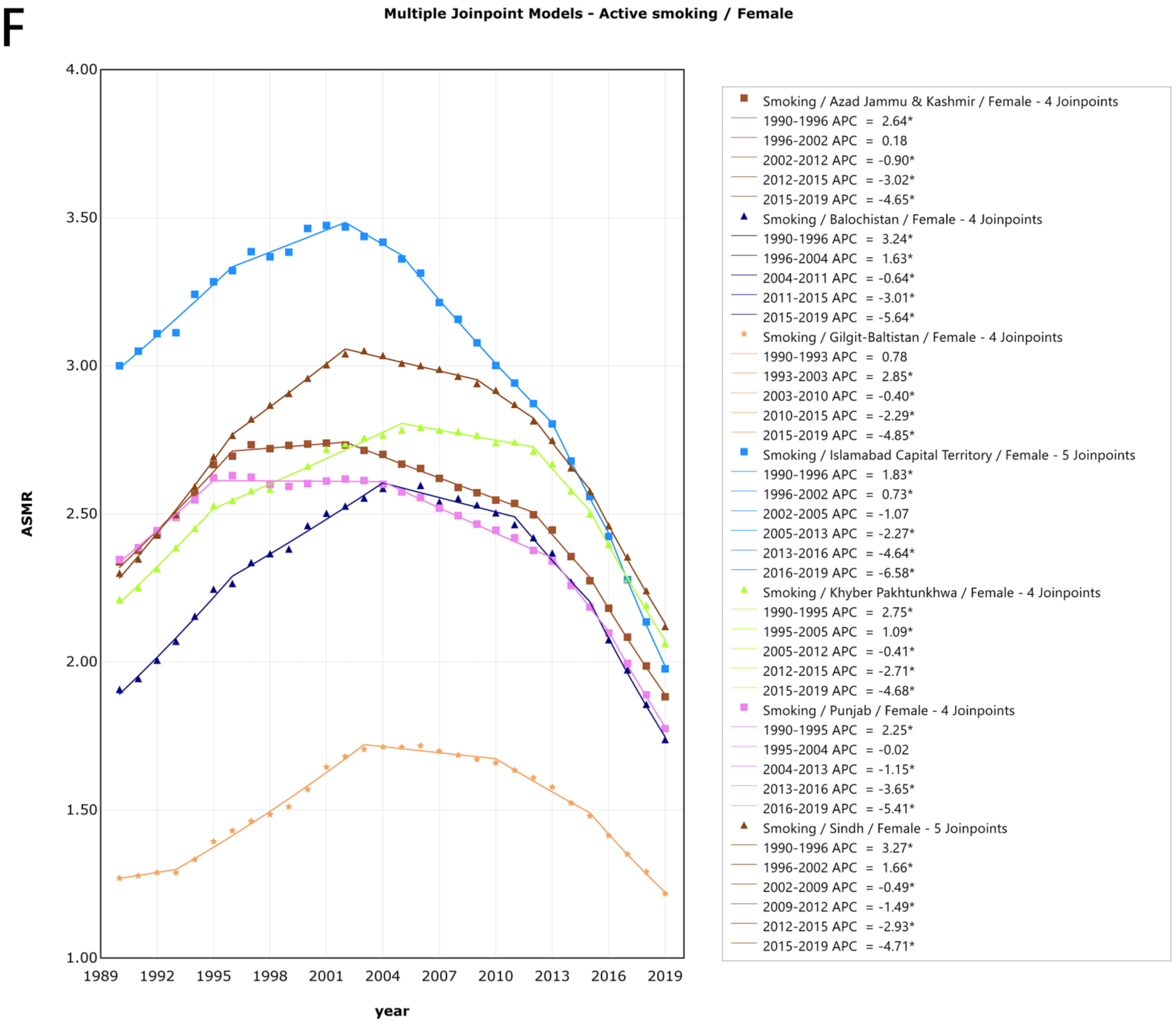

Multiple Joinpoint Models - Active smoking / Female
ASMR
year
4.00
3.50
3.00
2.50
2.00
1.50
1.00
1989
1992
1995
1998
2001
2004
2007
2010
2013
2016
2019
Smoking / Azad Jammu & Kashmir / Female - 4 Joinpoints
1990-1996 APC = 2.64*
1996-2002 APC = 0.18
2002-2012 APC = -0.90*
2012-2015 APC = -3.02*
2015-2019 APC = -4.65*
Smoking / Balochistan / Female - 4 Joinpoints
1990-1996 APC = 3.24*
1996-2004 APC = 1.63*
2004-2011 APC = -0.64*
2011-2015 APC = -3.01*
2015-2019 APC = -5.64*
Smoking / Gilgit-Baltistan / Female - 4 Joinpoints
1990-1993 APC = 0.78
1993-2003 APC = 2.85*
2003-2010 APC = -0.40*
2010-2015 APC = -2.29*
2015-2019 APC = -4.85*
Smoking / Islamabad Capital Territory / Female - 5 Joinpoints
1990-1996 APC = 1.83*
1996-2002 APC = 0.73*
2002-2005 APC = -1.07
2005-2013 APC = -2.27*
2013-2016 APC = -4.64*
2016-2019 APC = -6.58*
Smoking / Khyber Pakhtunkhwa / Female - 4 Joinpoints
1990-1995 APC = 2.75*
1995-2005 APC = 1.09*
2005-2012 APC = -0.41*
2012-2015 APC = -2.71*
2015-2019 APC = -4.68*
Smoking / Punjab / Female - 4 Joinpoints
1990-1995 APC = 2.25*
1995-2004 APC = -0.02
2004-2013 APC = -1.15*
2013-2016 APC = -3.65*
2016-2019 APC = -5.41*
Smoking / Sindh / Female - 5 Joinpoints
1990-1996 APC = 3.27*
1996-2002 APC = 1.66*
2002-2009 APC = -0.49*
2009-2012 APC = -1.49*
2012-2015 APC = -2.93*
2015-2019 APC = -4.71*

G

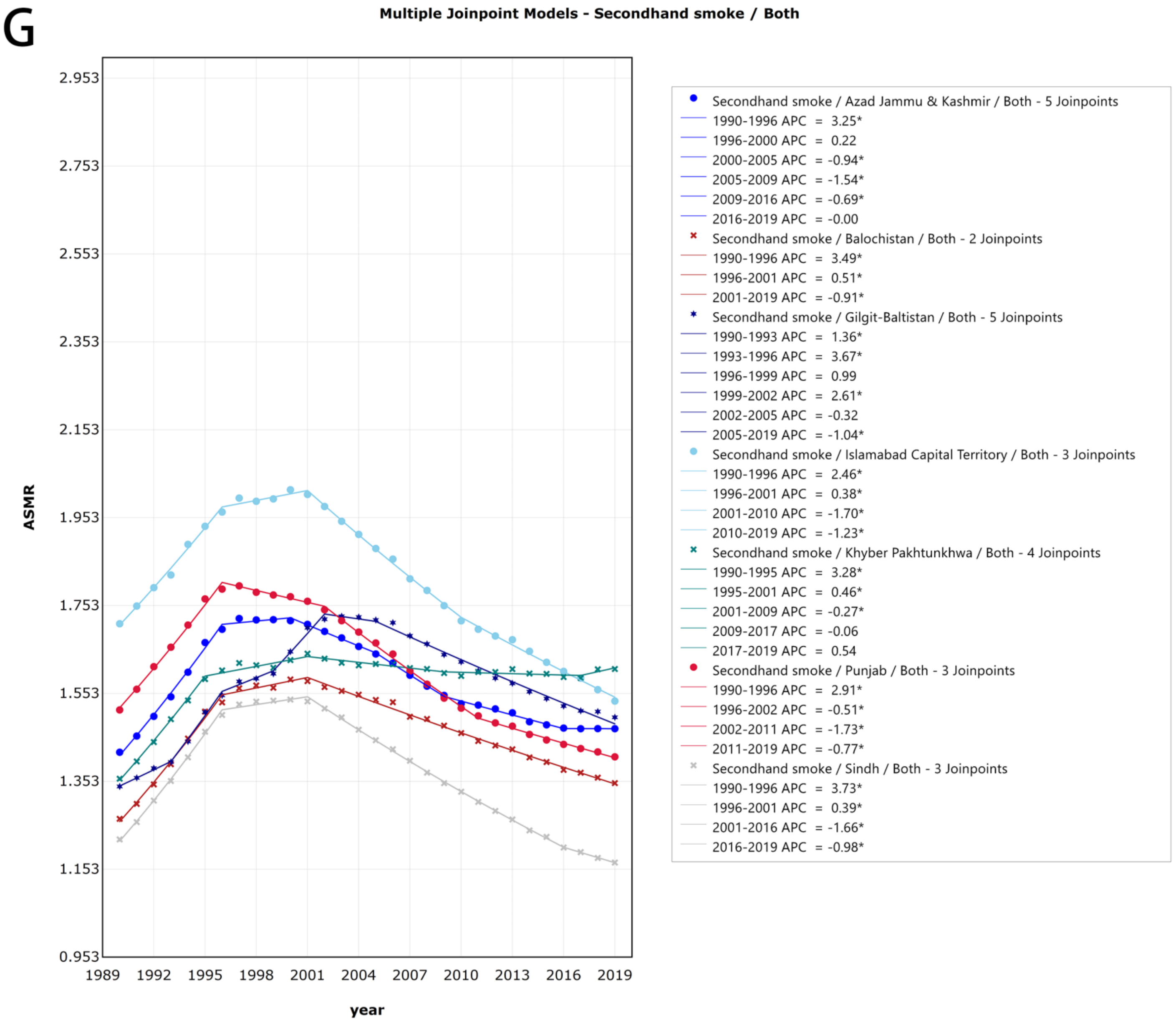

Multiple Joinpoint Models - Secondhand smoke / Both
ASMR
year
2.953
2.753
2.553
2.353
2.153
1.953
1.753
1.553
1.353
1.153
0.953
1989
1992
1995
1998
2001
2004
2007
2010
2013
2016
2019
Secondhand smoke / Azad Jammu & Kashmir / Both - 5 Joinpoints
1990-1996 APC = 3.25*
1996-2000 APC = 0.22
2000-2005 APC = -0.94*
2005-2009 APC = -1.54*
2009-2016 APC = -0.69*
2016-2019 APC = -0.00
Secondhand smoke / Balochistan / Both - 2 Joinpoints
1990-1996 APC = 3.49*
1996-2001 APC = 0.51*
2001-2019 APC = -0.91*
Secondhand smoke / Gilgit-Baltistan / Both - 5 Joinpoints
1990-1993 APC = 1.36*
1993-1996 APC = 3.67*
1996-1999 APC = 0.99
1999-2002 APC = 2.61*
2002-2005 APC = -0.32
2005-2019 APC = -1.04*
Secondhand smoke / Islamabad Capital Territory / Both - 3 Joinpoints
1990-1996 APC = 2.46*
1996-2001 APC = 0.38*
2001-2010 APC = -1.70*
2010-2019 APC = -1.23*
Secondhand smoke / Khyber Pakhtunkhwa / Both - 4 Joinpoints
1990-1995 APC = 3.28*
1995-2001 APC = 0.46*
2001-2009 APC = -0.27*
2009-2017 APC = -0.06
2017-2019 APC = 0.54
Secondhand smoke / Punjab / Both - 3 Joinpoints
1990-1996 APC = 2.91*
1996-2002 APC = -0.51*
2002-2011 APC = -1.73*
2011-2019 APC = -0.77*
Secondhand smoke / Sindh / Both - 3 Joinpoints
1990-1996 APC = 3.73*
1996-2001 APC = 0.39*
2001-2016 APC = -1.66*
2016-2019 APC = -0.98*

H

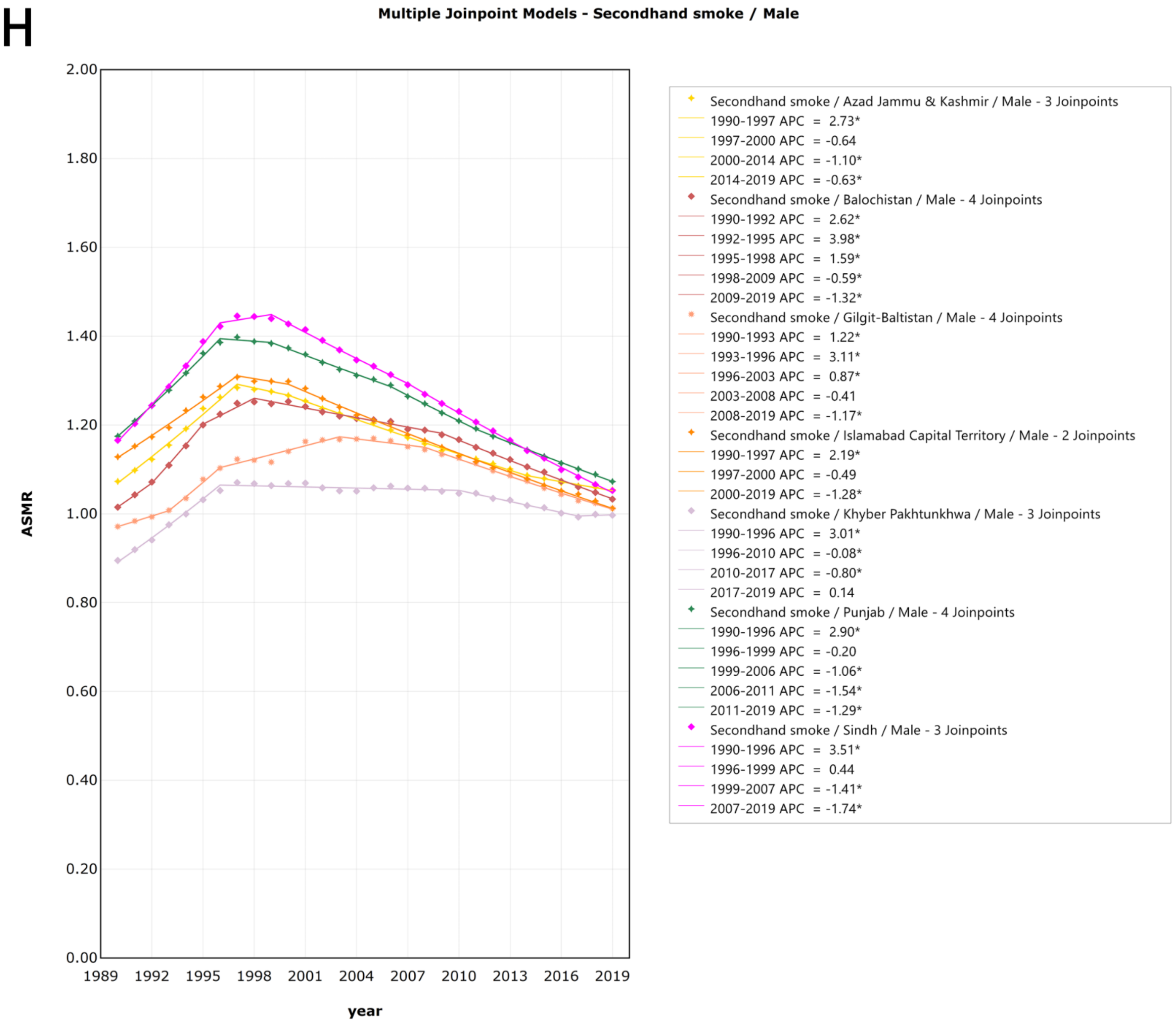

Multiple Joinpoint Models - Secondhand smoke / Male
ASMR
year
2.00
1.80
1.60
1.40
1.20
1.00
0.80
0.60
0.40
0.20
0.00
1989 1992 1995 1998 2001 2004 2007 2010 2013 2016 2019
Secondhand smoke / Azad Jammu & Kashmir / Male - 3 Joinpoints
1990-1997 APC = 2.73*
1997-2000 APC = -0.64
2000-2014 APC = -1.10*
2014-2019 APC = -0.63*
Secondhand smoke / Balochistan / Male - 4 Joinpoints
1990-1992 APC = 2.62*
1992-1995 APC = 3.98*
1995-1998 APC = 1.59*
1998-2009 APC = -0.59*
2009-2019 APC = -1.32*
Secondhand smoke / Gilgit-Baltistan / Male - 4 Joinpoints
1990-1993 APC = 1.22*
1993-1996 APC = 3.11*
1996-2003 APC = 0.87*
2003-2008 APC = -0.41
2008-2019 APC = -1.17*
Secondhand smoke / Islamabad Capital Territory / Male - 2 Joinpoints
1990-1997 APC = 2.19*
1997-2000 APC = -0.49
2000-2019 APC = -1.28*
Secondhand smoke / Khyber Pakhtunkhwa / Male - 3 Joinpoints
1990-1996 APC = 3.01*
1996-2010 APC = -0.08*
2010-2017 APC = -0.80*
2017-2019 APC = 0.14
Secondhand smoke / Punjab / Male - 4 Joinpoints
1990-1996 APC = 2.90*
1996-1999 APC = -0.20
1999-2006 APC = -1.06*
2006-2011 APC = -1.54*
2011-2019 APC = -1.29*
Secondhand smoke / Sindh / Male - 3 Joinpoints
1990-1996 APC = 3.51*
1996-1999 APC = 0.44
1999-2007 APC = -1.41*
2007-2019 APC = -1.74*

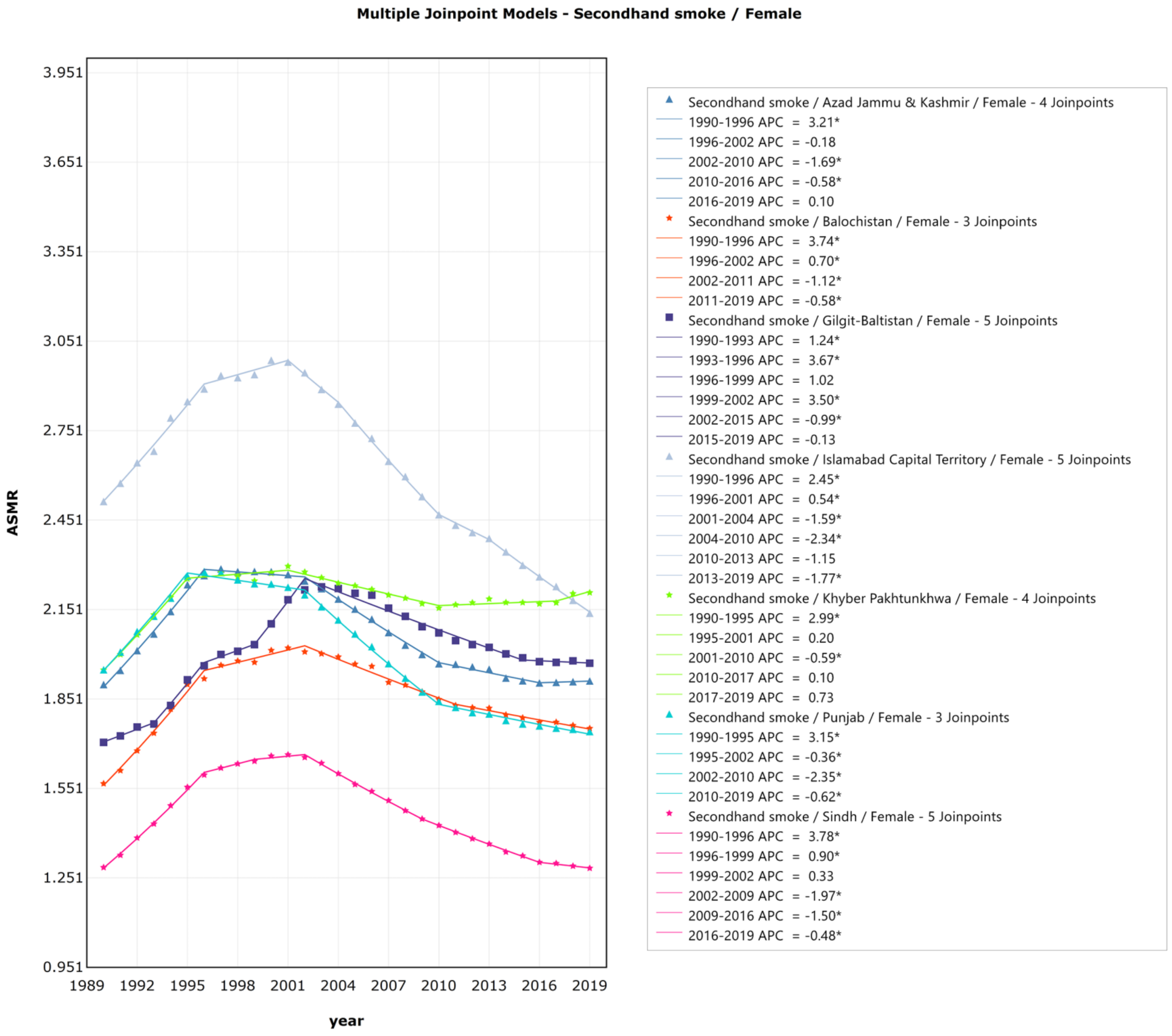
Multiple Joinpoint Models - Secondhand smoke / Female
ASMR
year
Secondhand smoke / Azad Jammu & Kashmir / Female - 4 Joinpoints
1990-1996 APC = 3.21*
1996-2002 APC = -0.18
2002-2010 APC = -1.69*
2010-2016 APC = -0.58*
2016-2019 APC = 0.10
Secondhand smoke / Balochistan / Female - 3 Joinpoints
1990-1996 APC = 3.74*
1996-2002 APC = 0.70*
2002-2011 APC = -1.12*
2011-2019 APC = -0.58*
Secondhand smoke / Gilgit-Baltistan / Female - 5 Joinpoints
1990-1993 APC = 1.24*
1993-1996 APC = 3.67*
1996-1999 APC = 1.02
1999-2002 APC = 3.50*
2002-2015 APC = -0.99*
2015-2019 APC = -0.13
Secondhand smoke / Islamabad Capital Territory / Female - 5 Joinpoints
1990-1996 APC = 2.45*
1996-2001 APC = 0.54*
2001-2004 APC = -1.59*
2004-2010 APC = -2.34*
2010-2013 APC = -1.15
2013-2019 APC = -1.77*
Secondhand smoke / Khyber Pakhtunkhwa / Female - 4 Joinpoints
1990-1995 APC = 2.99*
1995-2001 APC = 0.20
2001-2010 APC = -0.59*
2010-2017 APC = 0.10
2017-2019 APC = 0.73
Secondhand smoke / Punjab / Female - 3 Joinpoints
1990-1995 APC = 3.15*
1995-2002 APC = -0.36*
2002-2010 APC = -2.35*
2010-2019 APC = -0.62*
Secondhand smoke / Sindh / Female - 5 Joinpoints
1990-1996 APC = 3.78*
1996-1999 APC = 0.90*
1999-2002 APC = 0.33
2002-2009 APC = -1.97*
2009-2016 APC = -1.50*
2016-2019 APC = -0.48*

Supplementary Figure 6. APC of ASMR in ischemic stroke due to tobacco use, active smoking and second-hand smoke by gender in various regions of Pakistan from 1990 to 2019.

A. APC of ASMR in ischemic stroke due to tobacco use in various regions of Pakistan from 1990 to 2019.

B. APC of ASMR in ischemic stroke caused by tobacco use among men in various regions of Pakistan from 1990 to 2019.

C. APC of ASMR in ischemic stroke caused by tobacco use among women in various regions of Pakistan from 1990 to 2019.

D. APC of ASMR in ischemic stroke due to active smoking in various regions of Pakistan from 1990 to 2019.

E. APC of ASMR in ischemic stroke caused by active smoking among men in various regions of Pakistan from 1990 to 2019.

F. APC of ASMR in ischemic stroke caused by active smoking among women in various regions of Pakistan from 1990 to 2019.

G. APC of ASMR in ischemic stroke due to secondhand smoke in various regions of Pakistan from 1990 to 2019.

H. APC of ASMR in ischemic stroke caused by secondhand smoke among men in various regions of Pakistan from 1990 to 2019.

I. APC of ASMR in ischemic stroke caused by secondhand smoke among women in various regions of Pakistan from 1990 to 2019.

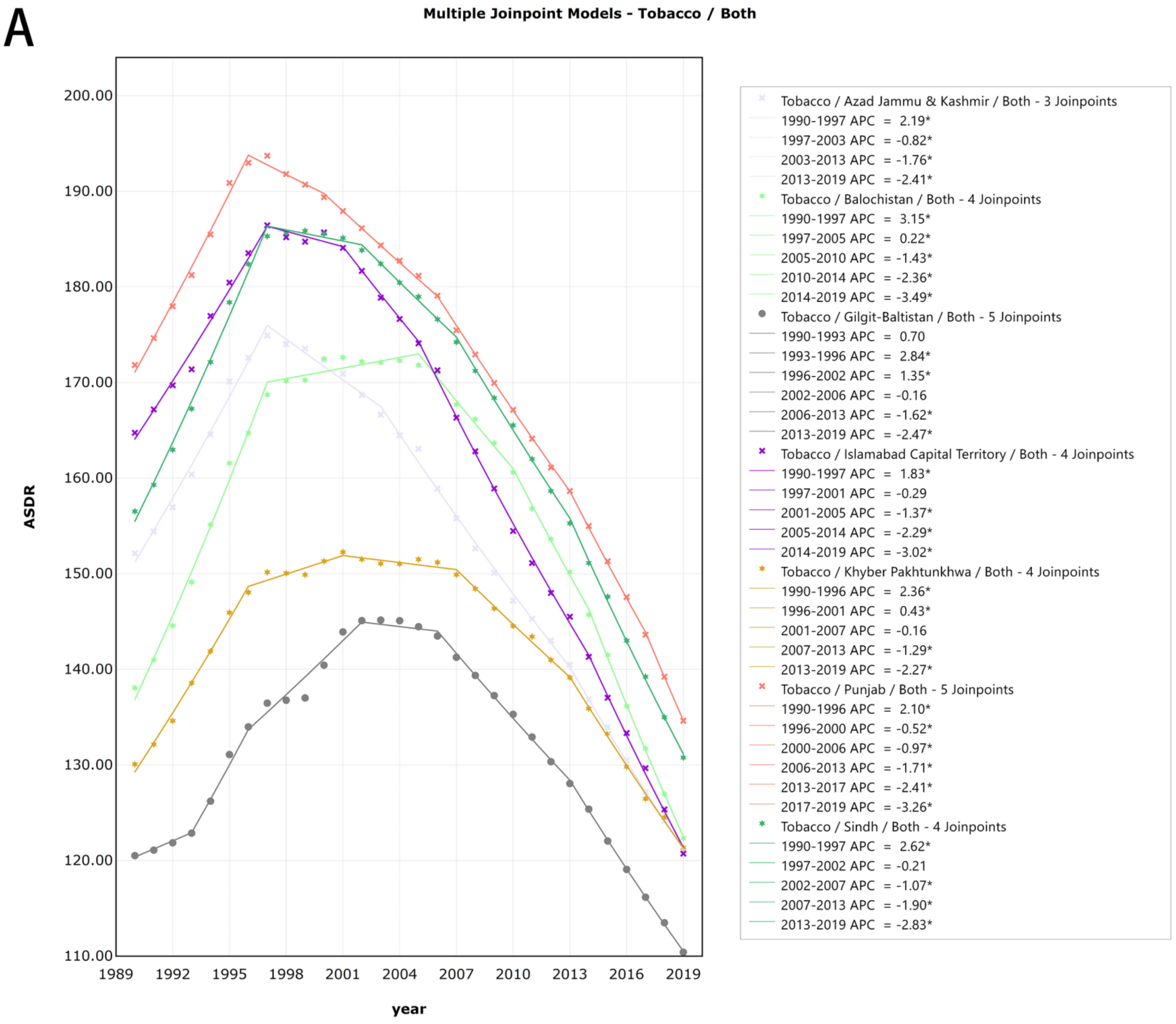

A
Multiple Joinpoint Models - Tobacco / Both
ASDR
200.00
190.00
180.00
170.00
160.00
150.00
140.00
130.00
120.00
110.00
1989
1992
1995
1998
2001
2004
2007
2010
2013
2016
2019
year
Tobacco / Azad Jammu & Kashmir / Both - 3 Joinpoints
1990-1997 APC = 2.19*
1997-2003 APC = -0.82*
2003-2013 APC = -1.76*
2013-2019 APC = -2.41*
Tobacco / Balochistan / Both - 4 Joinpoints
1990-1997 APC = 3.15*
1997-2005 APC = 0.22*
2005-2010 APC = -1.43*
2010-2014 APC = -2.36*
2014-2019 APC = -3.49*
Tobacco / Gilgit-Baltistan / Both - 5 Joinpoints
1990-1993 APC = 0.70
1993-1996 APC = 2.84*
1996-2002 APC = 1.35*
2002-2006 APC = -0.16
2006-2013 APC = -1.62*
2013-2019 APC = -2.47*
Tobacco / Islamabad Capital Territory / Both - 4 Joinpoints
1990-1997 APC = 1.83*
1997-2001 APC = -0.29
2001-2005 APC = -1.37*
2005-2014 APC = -2.29*
2014-2019 APC = -3.02*
Tobacco / Khyber Pakhtunkhwa / Both - 4 Joinpoints
1990-1996 APC = 2.36*
1996-2001 APC = 0.43*
2001-2007 APC = -0.16
2007-2013 APC = -1.29*
2013-2019 APC = -2.27*
Tobacco / Punjab / Both - 5 Joinpoints
1990-1996 APC = 2.10*
1996-2000 APC = -0.52*
2000-2006 APC = -0.97*
2006-2013 APC = -1.71*
2013-2017 APC = -2.41*
2017-2019 APC = -3.26*
Tobacco / Sindh / Both - 4 Joinpoints
1990-1997 APC = 2.62*
1997-2002 APC = -0.21
2002-2007 APC = -1.07*
2007-2013 APC = -1.90*
2013-2019 APC = -2.83*

B

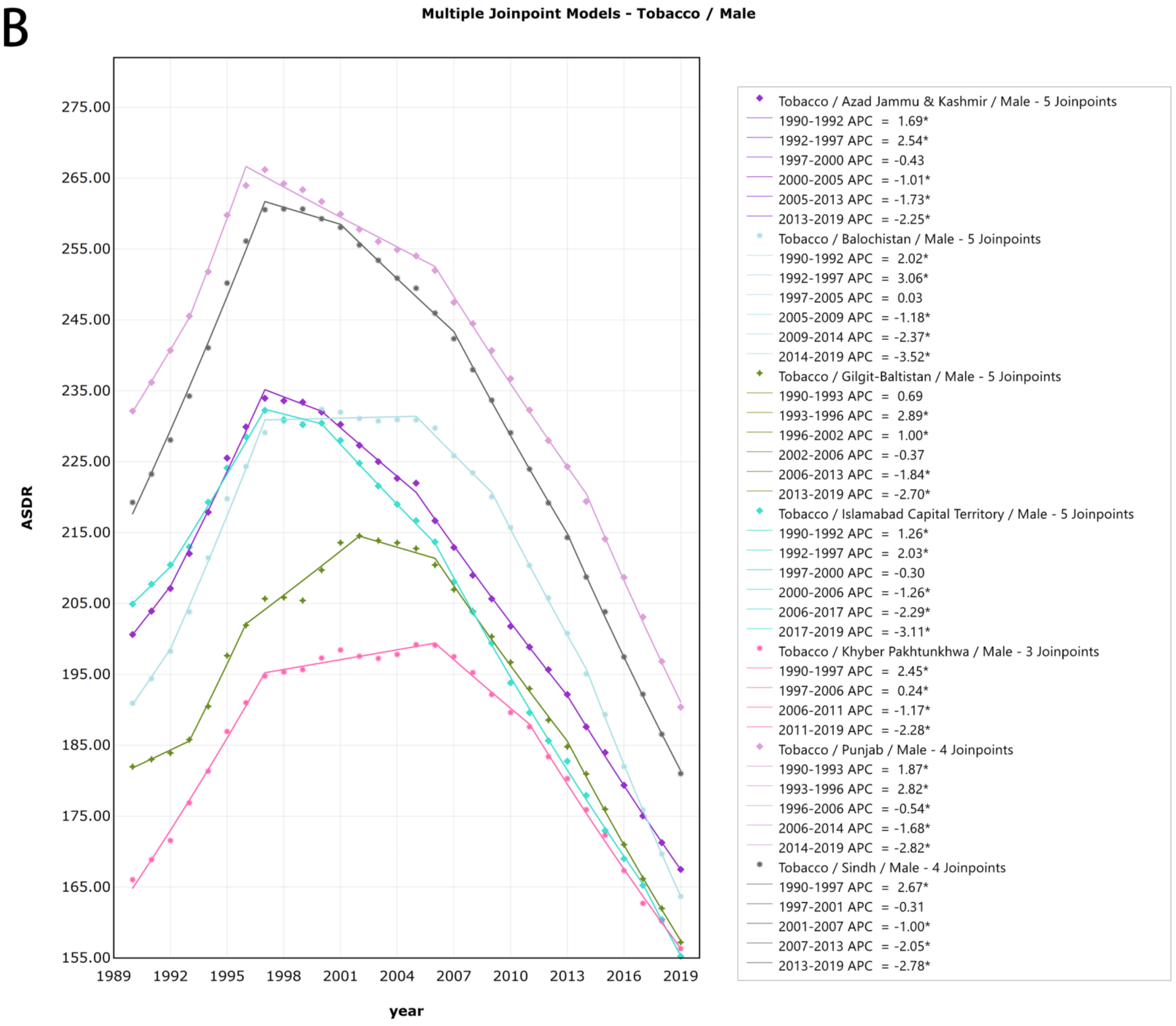

Multiple Joinpoint Models - Tobacco / Male
ASDR
year
275.00
265.00
255.00
245.00
235.00
225.00
215.00
205.00
195.00
185.00
175.00
165.00
155.00
1989
1992
1995
1998
2001
2004
2007
2010
2013
2016
2019
Tobacco / Azad Jammu & Kashmir / Male - 5 Joinpoints
1990-1992 APC = 1.69*
1992-1997 APC = 2.54*
1997-2000 APC = -0.43
2000-2005 APC = -1.01*
2005-2013 APC = -1.73*
2013-2019 APC = -2.25*
Tobacco / Balochistan / Male - 5 Joinpoints
1990-1992 APC = 2.02*
1992-1997 APC = 3.06*
1997-2005 APC = 0.03
2005-2009 APC = -1.18*
2009-2014 APC = -2.37*
2014-2019 APC = -3.52*
Tobacco / Gilgit-Baltistan / Male - 5 Joinpoints
1990-1993 APC = 0.69
1993-1996 APC = 2.89*
1996-2002 APC = 1.00*
2002-2006 APC = -0.37
2006-2013 APC = -1.84*
2013-2019 APC = -2.70*
Tobacco / Islamabad Capital Territory / Male - 5 Joinpoints
1990-1992 APC = 1.26*
1992-1997 APC = 2.03*
1997-2000 APC = -0.30
2000-2006 APC = -1.26*
2006-2017 APC = -2.29*
2017-2019 APC = -3.11*
Tobacco / Khyber Pakhtunkhwa / Male - 3 Joinpoints
1990-1997 APC = 2.45*
1997-2006 APC = 0.24*
2006-2011 APC = -1.17*
2011-2019 APC = -2.28*
Tobacco / Punjab / Male - 4 Joinpoints
1990-1993 APC = 1.87*
1993-1996 APC = 2.82*
1996-2006 APC = -0.54*
2006-2014 APC = -1.68*
2014-2019 APC = -2.82*
Tobacco / Sindh / Male - 4 Joinpoints
1990-1997 APC = 2.67*
1997-2001 APC = -0.31
2001-2007 APC = -1.00*
2007-2013 APC = -2.05*
2013-2019 APC = -2.78*

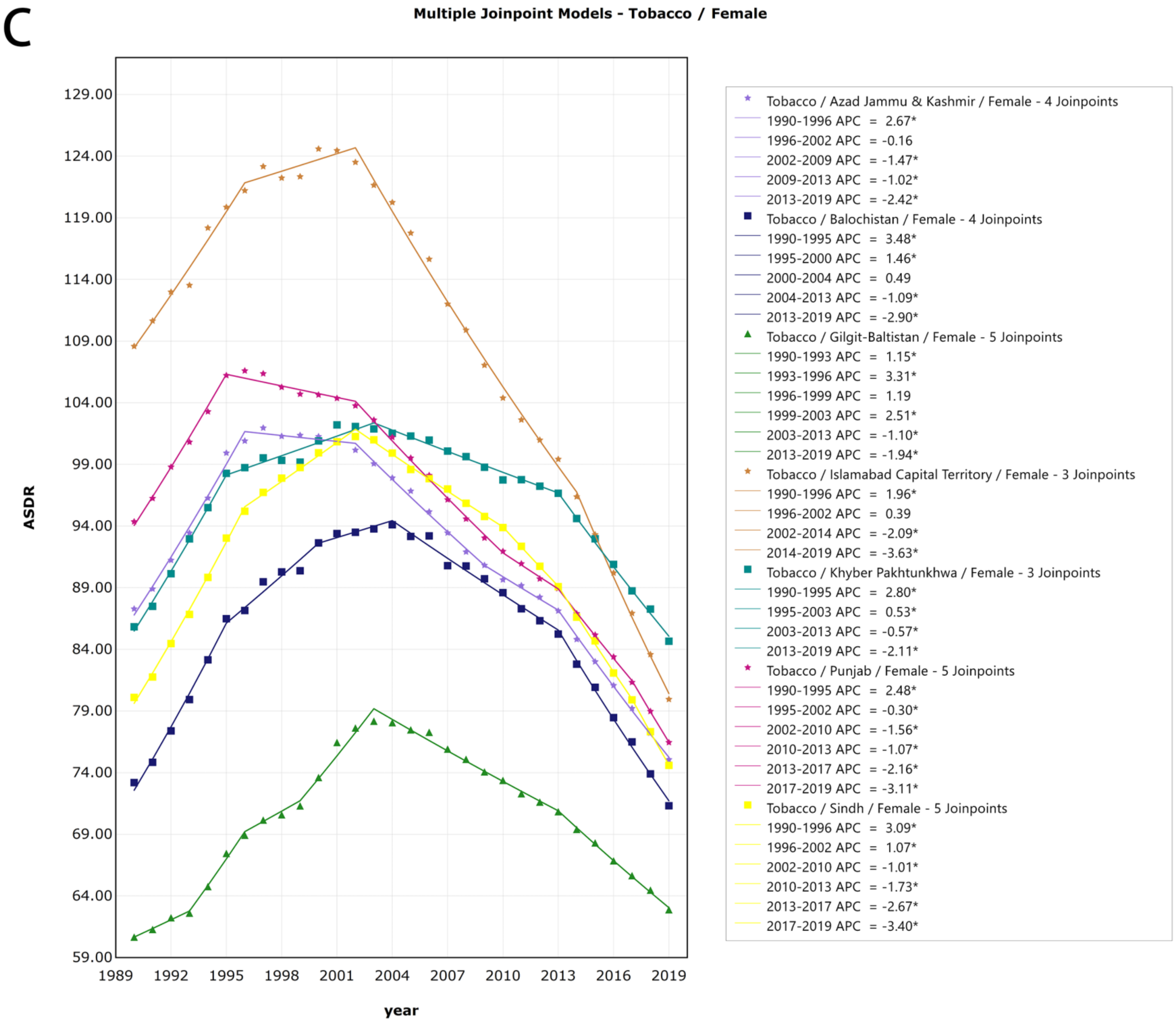
C
Multiple Joinpoint Models - Tobacco / Female
ASDR
year
129.00
124.00
119.00
114.00
109.00
104.00
99.00
94.00
89.00
84.00
79.00
74.00
69.00
64.00
59.00
1989
1992
1995
1998
2001
2004
2007
2010
2013
2016
2019
Tobacco / Azad Jammu & Kashmir / Female - 4 Joinpoints
1990-1996 APC = 2.67*
1996-2002 APC = -0.16
2002-2009 APC = -1.47*
2009-2013 APC = -1.02*
2013-2019 APC = -2.42*
Tobacco / Balochistan / Female - 4 Joinpoints
1990-1995 APC = 3.48*
1995-2000 APC = 1.46*
2000-2004 APC = 0.49
2004-2013 APC = -1.09*
2013-2019 APC = -2.90*
Tobacco / Gilgit-Baltistan / Female - 5 Joinpoints
1990-1993 APC = 1.15*
1993-1996 APC = 3.31*
1996-1999 APC = 1.19
1999-2003 APC = 2.51*
2003-2013 APC = -1.10*
2013-2019 APC = -1.94*
Tobacco / Islamabad Capital Territory / Female - 3 Joinpoints
1990-1996 APC = 1.96*
1996-2002 APC = 0.39
2002-2014 APC = -2.09*
2014-2019 APC = -3.63*
Tobacco / Khyber Pakhtunkhwa / Female - 3 Joinpoints
1990-1995 APC = 2.80*
1995-2003 APC = 0.53*
2003-2013 APC = -0.57*
2013-2019 APC = -2.11*
Tobacco / Punjab / Female - 5 Joinpoints
1990-1995 APC = 2.48*
1995-2002 APC = -0.30*
2002-2010 APC = -1.56*
2010-2013 APC = -1.07*
2013-2017 APC = -2.16*
2017-2019 APC = -3.11*
Tobacco / Sindh / Female - 5 Joinpoints
1990-1996 APC = 3.09*
1996-2002 APC = 1.07*
2002-2010 APC = -1.01*
2010-2013 APC = -1.73*
2013-2017 APC = -2.67*
2017-2019 APC = -3.40*

D

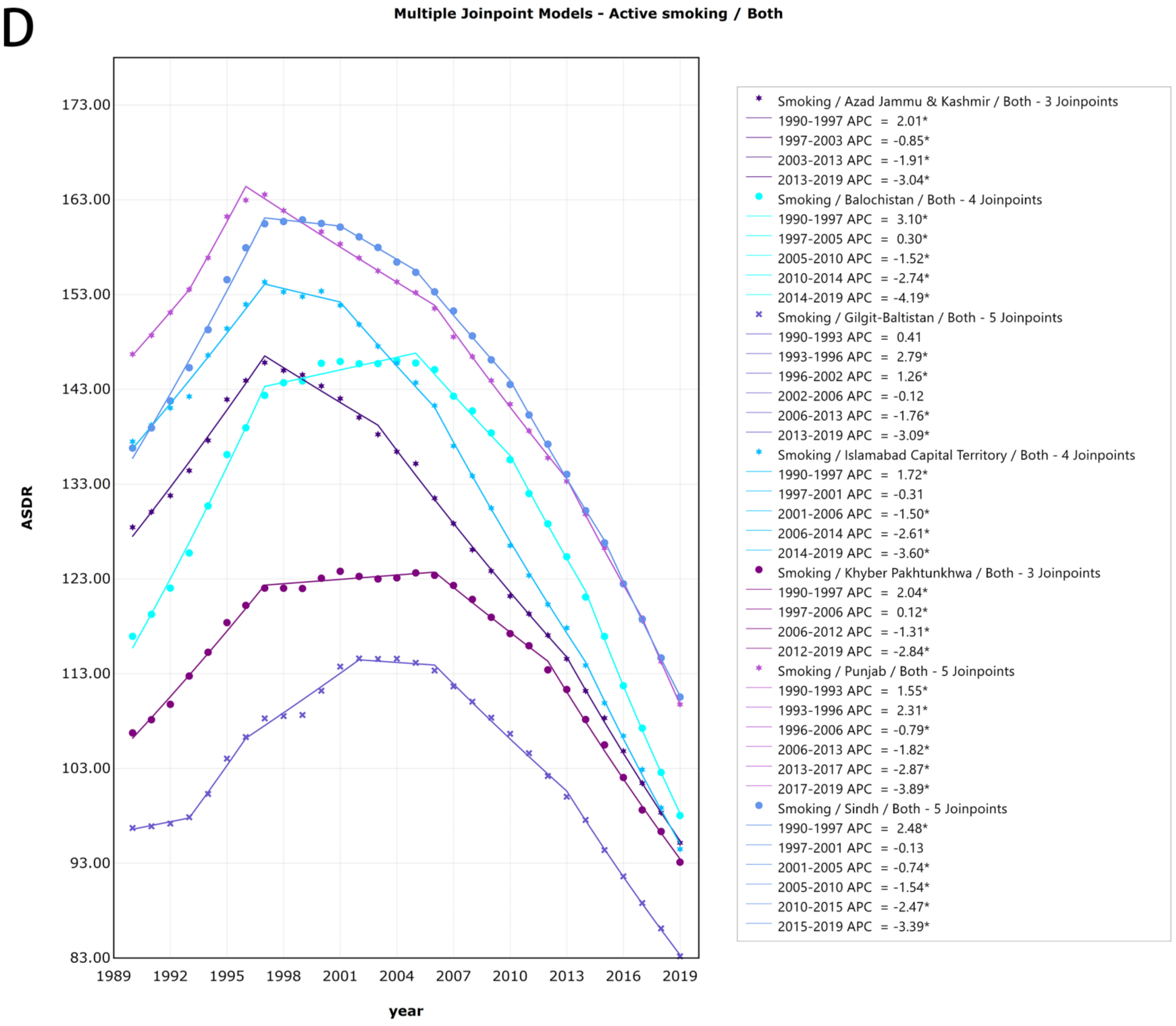

Multiple Joinpoint Models - Active smoking / Both
ASDR
year
173.00
163.00
153.00
143.00
133.00
123.00
113.00
103.00
93.00
83.00
1989
1992
1995
1998
2001
2004
2007
2010
2013
2016
2019
Smoking / Azad Jammu & Kashmir / Both - 3 Joinpoints
1990-1997 APC = 2.01*
1997-2003 APC = -0.85*
2003-2013 APC = -1.91*
2013-2019 APC = -3.04*
Smoking / Balochistan / Both - 4 Joinpoints
1990-1997 APC = 3.10*
1997-2005 APC = 0.30*
2005-2010 APC = -1.52*
2010-2014 APC = -2.74*
2014-2019 APC = -4.19*
Smoking / Gilgit-Baltistan / Both - 5 Joinpoints
1990-1993 APC = 0.41
1993-1996 APC = 2.79*
1996-2002 APC = 1.26*
2002-2006 APC = -0.12
2006-2013 APC = -1.76*
2013-2019 APC = -3.09*
Smoking / Islamabad Capital Territory / Both - 4 Joinpoints
1990-1997 APC = 1.72*
1997-2001 APC = -0.31
2001-2006 APC = -1.50*
2006-2014 APC = -2.61*
2014-2019 APC = -3.60*
Smoking / Khyber Pakhtunkhwa / Both - 3 Joinpoints
1990-1997 APC = 2.04*
1997-2006 APC = 0.12*
2006-2012 APC = -1.31*
2012-2019 APC = -2.84*
Smoking / Punjab / Both - 5 Joinpoints
1990-1993 APC = 1.55*
1993-1996 APC = 2.31*
1996-2006 APC = -0.79*
2006-2013 APC = -1.82*
2013-2017 APC = -2.87*
2017-2019 APC = -3.89*
Smoking / Sindh / Both - 5 Joinpoints
1990-1997 APC = 2.48*
1997-2001 APC = -0.13
2001-2005 APC = -0.74*
2005-2010 APC = -1.54*
2010-2015 APC = -2.47*
2015-2019 APC = -3.39*

E

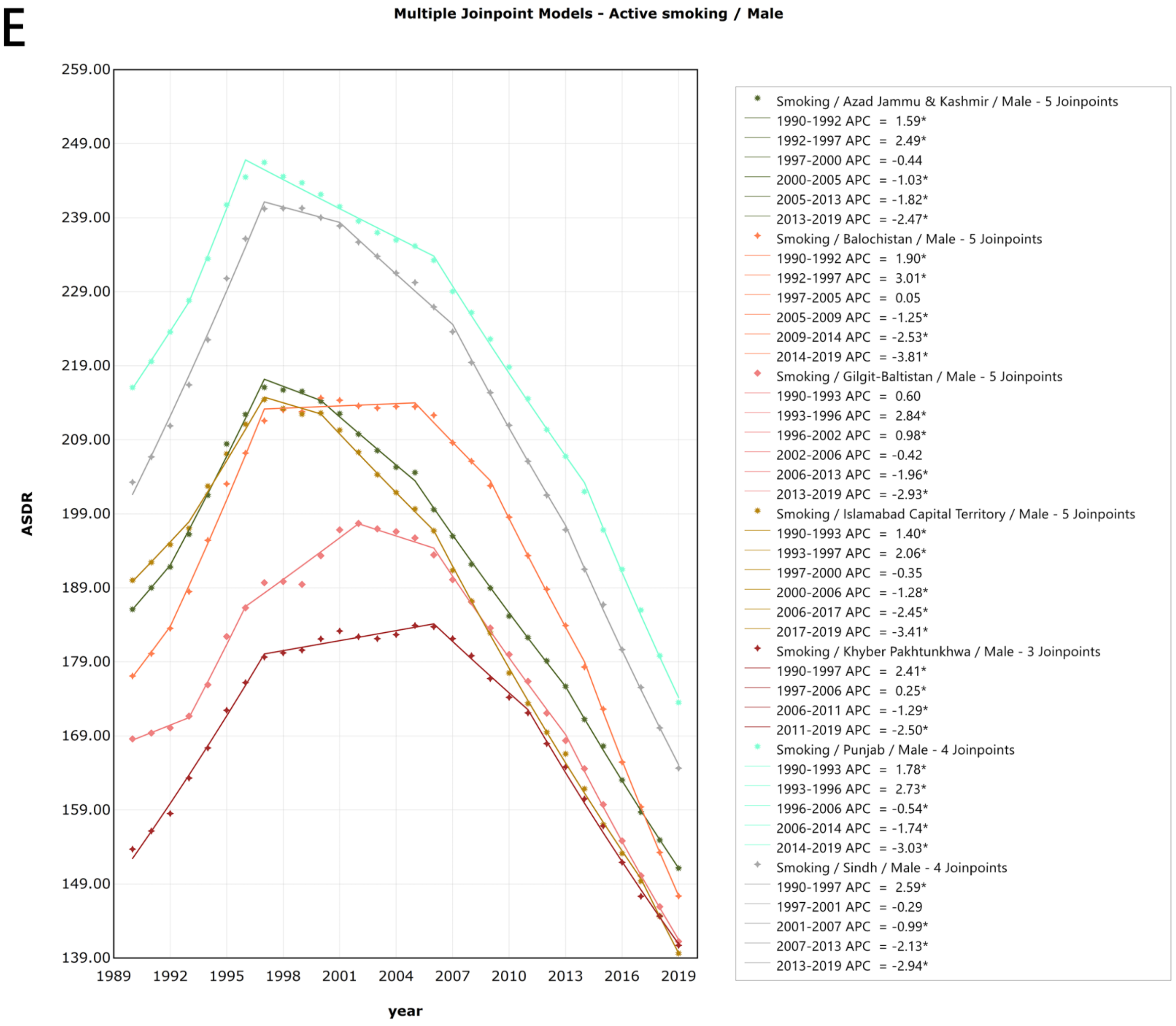

Multiple Joinpoint Models - Active smoking / Male
ASDR
259.00
249.00
239.00
229.00
219.00
209.00
199.00
189.00
179.00
169.00
159.00
149.00
139.00
1989
1992
1995
1998
2001
2004
2007
2010
2013
2016
2019
year
Smoking / Azad Jammu & Kashmir / Male - 5 Joinpoints
1990-1992 APC = 1.59*
1992-1997 APC = 2.49*
1997-2000 APC = -0.44
2000-2005 APC = -1.03*
2005-2013 APC = -1.82*
2013-2019 APC = -2.47*
Smoking / Balochistan / Male - 5 Joinpoints
1990-1992 APC = 1.90*
1992-1997 APC = 3.01*
1997-2005 APC = 0.05
2005-2009 APC = -1.25*
2009-2014 APC = -2.53*
2014-2019 APC = -3.81*
Smoking / Gilgit-Baltistan / Male - 5 Joinpoints
1990-1993 APC = 0.60
1993-1996 APC = 2.84*
1996-2002 APC = 0.98*
2002-2006 APC = -0.42
2006-2013 APC = -1.96*
2013-2019 APC = -2.93*
Smoking / Islamabad Capital Territory / Male - 5 Joinpoints
1990-1993 APC = 1.40*
1993-1997 APC = 2.06*
1997-2000 APC = -0.35
2000-2006 APC = -1.28*
2006-2017 APC = -2.45*
2017-2019 APC = -3.41*
Smoking / Khyber Pakhtunkhwa / Male - 3 Joinpoints
1990-1997 APC = 2.41*
1997-2006 APC = 0.25*
2006-2011 APC = -1.29*
2011-2019 APC = -2.50*
Smoking / Punjab / Male - 4 Joinpoints
1990-1993 APC = 1.78*
1993-1996 APC = 2.73*
1996-2006 APC = -0.54*
2006-2014 APC = -1.74*
2014-2019 APC = -3.03*
Smoking / Sindh / Male - 4 Joinpoints
1990-1997 APC = 2.59*
1997-2001 APC = -0.29
2001-2007 APC = -0.99*
2007-2013 APC = -2.13*
2013-2019 APC = -2.94*

F

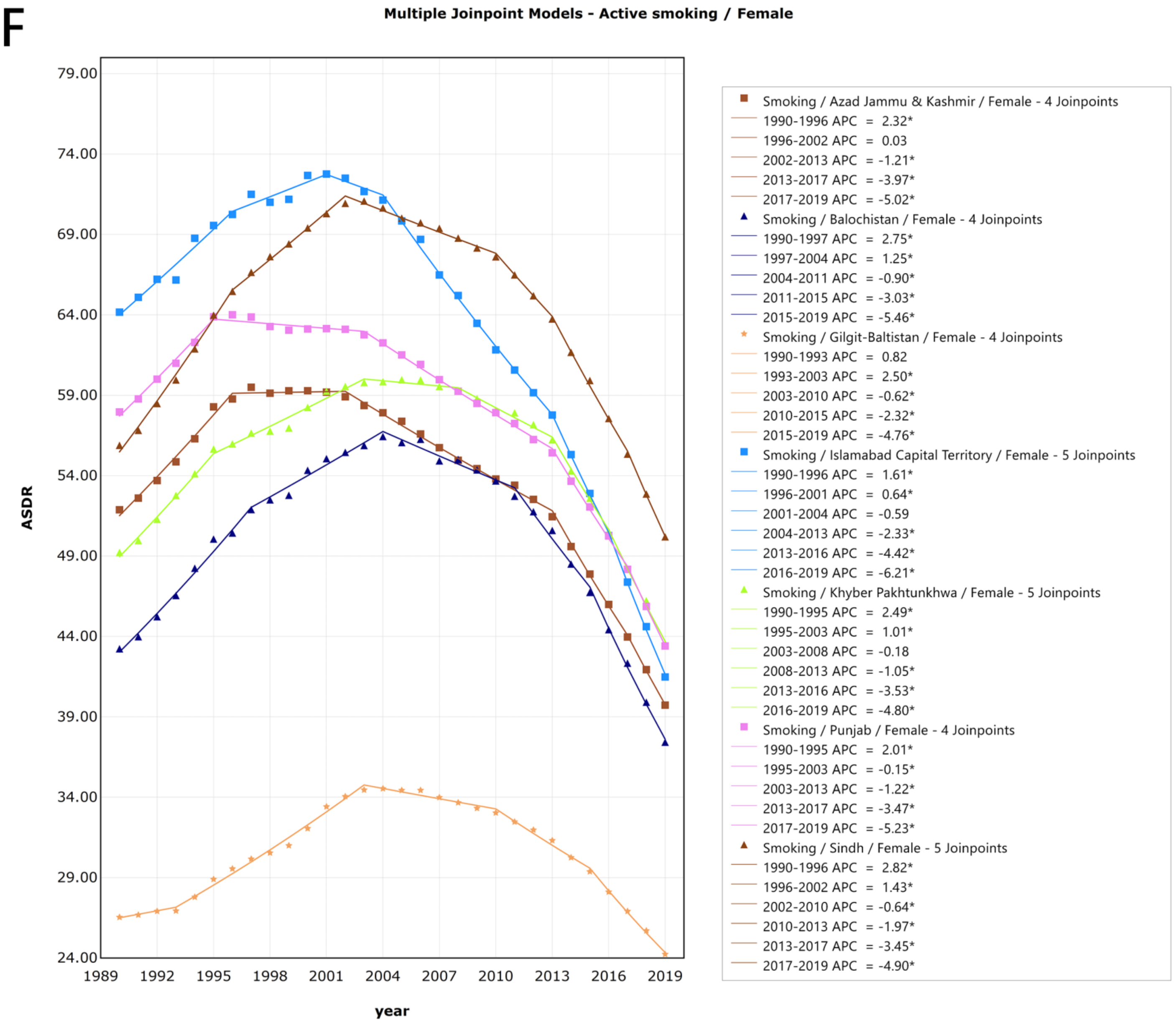

Multiple Joinpoint Models - Active smoking / Female
ASDR
year
79.00
74.00
69.00
64.00
59.00
54.00
49.00
44.00
39.00
34.00
29.00
24.00
1989
1992
1995
1998
2001
2004
2007
2010
2013
2016
2019
Smoking / Azad Jammu & Kashmir / Female - 4 Joinpoints
1990-1996 APC = 2.32*
1996-2002 APC = 0.03
2002-2013 APC = -1.21*
2013-2017 APC = -3.97*
2017-2019 APC = -5.02*
Smoking / Balochistan / Female - 4 Joinpoints
1990-1997 APC = 2.75*
1997-2004 APC = 1.25*
2004-2011 APC = -0.90*
2011-2015 APC = -3.03*
2015-2019 APC = -5.46*
Smoking / Gilgit-Baltistan / Female - 4 Joinpoints
1990-1993 APC = 0.82
1993-2003 APC = 2.50*
2003-2010 APC = -0.62*
2010-2015 APC = -2.32*
2015-2019 APC = -4.76*
Smoking / Islamabad Capital Territory / Female - 5 Joinpoints
1990-1996 APC = 1.61*
1996-2001 APC = 0.64*
2001-2004 APC = -0.59
2004-2013 APC = -2.33*
2013-2016 APC = -4.42*
2016-2019 APC = -6.21*
Smoking / Khyber Pakhtunkhwa / Female - 5 Joinpoints
1990-1995 APC = 2.49*
1995-2003 APC = 1.01*
2003-2008 APC = -0.18
2008-2013 APC = -1.05*
2013-2016 APC = -3.53*
2016-2019 APC = -4.80*
Smoking / Punjab / Female - 4 Joinpoints
1990-1995 APC = 2.01*
1995-2003 APC = -0.15*
2003-2013 APC = -1.22*
2013-2017 APC = -3.47*
2017-2019 APC = -5.23*
Smoking / Sindh / Female - 5 Joinpoints
1990-1996 APC = 2.82*
1996-2002 APC = 1.43*
2002-2010 APC = -0.64*
2010-2013 APC = -1.97*
2013-2017 APC = -3.45*
2017-2019 APC = -4.90*

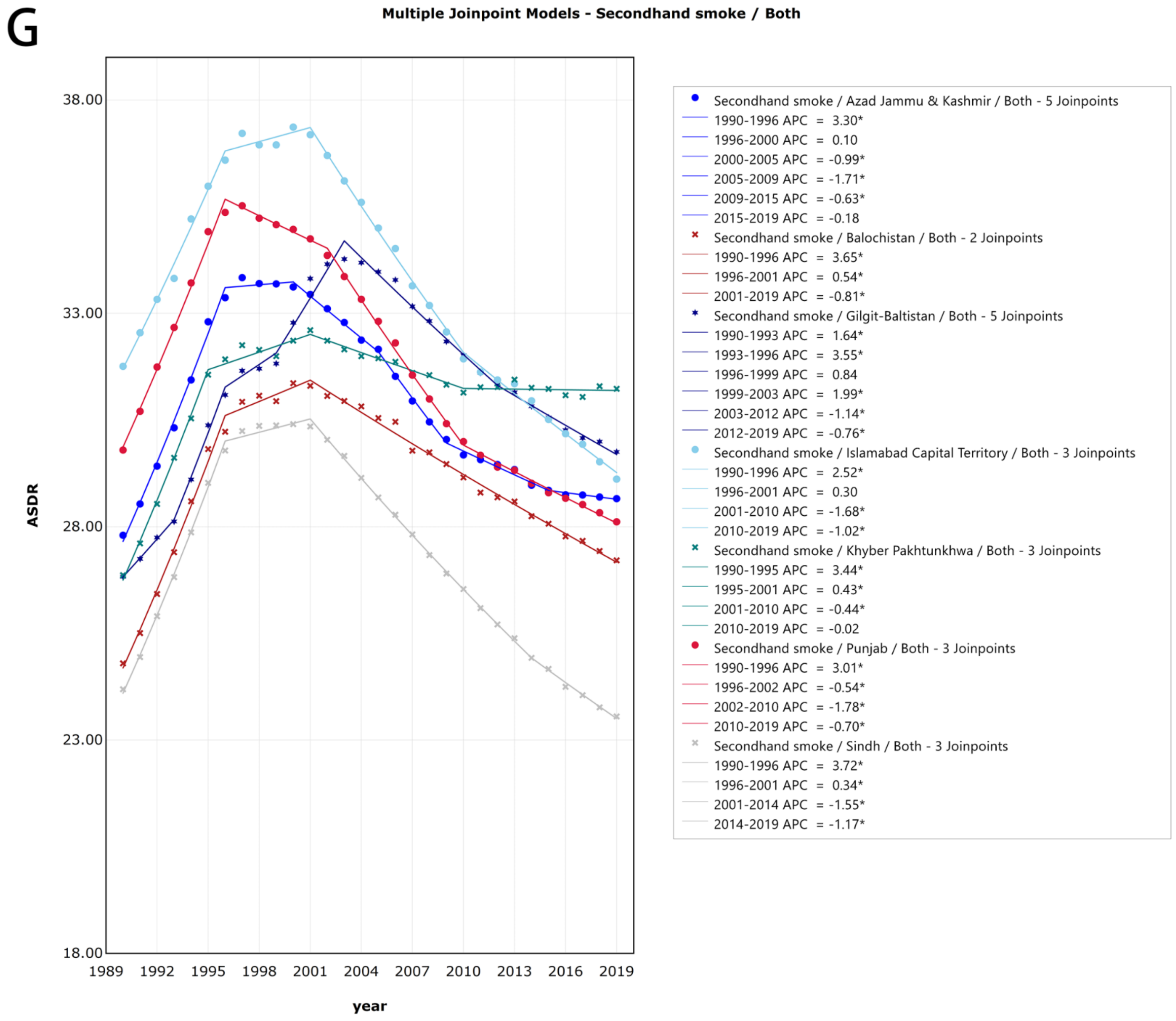

G
Multiple Joinpoint Models - Secondhand smoke / Both
ASDR
year
38.00
33.00
28.00
23.00
18.00
1989
1992
1995
1998
2001
2004
2007
2010
2013
2016
2019
Secondhand smoke / Azad Jammu & Kashmir / Both - 5 Joinpoints
1990-1996 APC = 3.30*
1996-2000 APC = 0.10
2000-2005 APC = -0.99*
2005-2009 APC = -1.71*
2009-2015 APC = -0.63*
2015-2019 APC = -0.18
Secondhand smoke / Balochistan / Both - 2 Joinpoints
1990-1996 APC = 3.65*
1996-2001 APC = 0.54*
2001-2019 APC = -0.81*
Secondhand smoke / Gilgit-Baltistan / Both - 5 Joinpoints
1990-1993 APC = 1.64*
1993-1996 APC = 3.55*
1996-1999 APC = 0.84
1999-2003 APC = 1.99*
2003-2012 APC = -1.14*
2012-2019 APC = -0.76*
Secondhand smoke / Islamabad Capital Territory / Both - 3 Joinpoints
1990-1996 APC = 2.52*
1996-2001 APC = 0.30
2001-2010 APC = -1.68*
2010-2019 APC = -1.02*
Secondhand smoke / Khyber Pakhtunkhwa / Both - 3 Joinpoints
1990-1995 APC = 3.44*
1995-2001 APC = 0.43*
2001-2010 APC = -0.44*
2010-2019 APC = -0.02
Secondhand smoke / Punjab / Both - 3 Joinpoints
1990-1996 APC = 3.01*
1996-2002 APC = -0.54*
2002-2010 APC = -1.78*
2010-2019 APC = -0.70*
Secondhand smoke / Sindh / Both - 3 Joinpoints
1990-1996 APC = 3.72*
1996-2001 APC = 0.34*
2001-2014 APC = -1.55*
2014-2019 APC = -1.17*

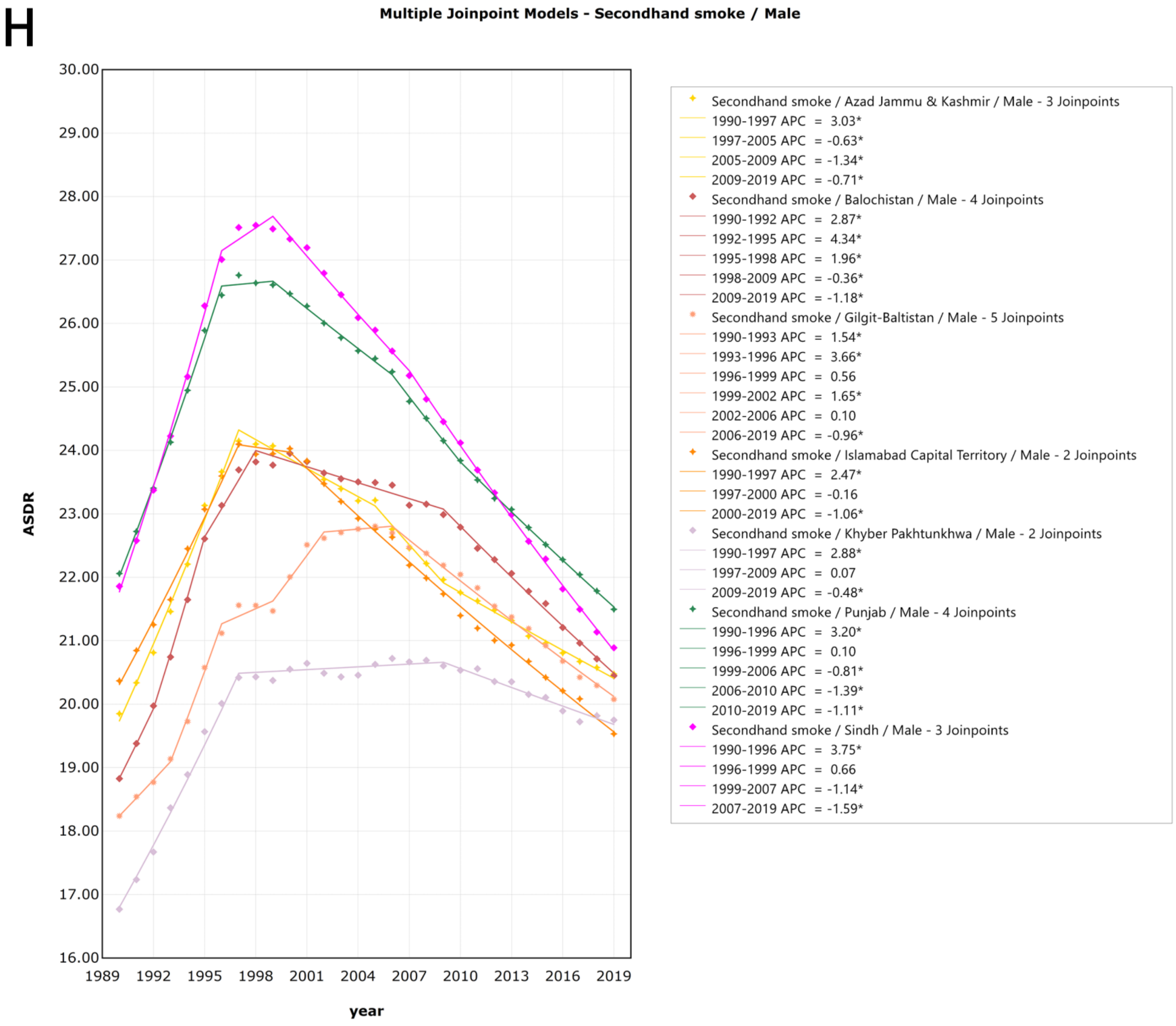
H
Multiple Joinpoint Models - Secondhand smoke / Male
ASDR
year
30.00
29.00
28.00
27.00
26.00
25.00
24.00
23.00
22.00
21.00
20.00
19.00
18.00
17.00
16.00
1989
1992
1995
1998
2001
2004
2007
2010
2013
2016
2019
Secondhand smoke / Azad Jammu & Kashmir / Male - 3 Joinpoints
1990-1997 APC = 3.03*
1997-2005 APC = -0.63*
2005-2009 APC = -1.34*
2009-2019 APC = -0.71*
Secondhand smoke / Balochistan / Male - 4 Joinpoints
1990-1992 APC = 2.87*
1992-1995 APC = 4.34*
1995-1998 APC = 1.96*
1998-2009 APC = -0.36*
2009-2019 APC = -1.18*
Secondhand smoke / Gilgit-Baltistan / Male - 5 Joinpoints
1990-1993 APC = 1.54*
1993-1996 APC = 3.66*
1996-1999 APC = 0.56
1999-2002 APC = 1.65*
2002-2006 APC = 0.10
2006-2019 APC = -0.96*
Secondhand smoke / Islamabad Capital Territory / Male - 2 Joinpoints
1990-1997 APC = 2.47*
1997-2000 APC = -0.16
2000-2019 APC = -1.06*
Secondhand smoke / Khyber Pakhtunkhwa / Male - 2 Joinpoints
1990-1997 APC = 2.88*
1997-2009 APC = 0.07
2009-2019 APC = -0.48*
Secondhand smoke / Punjab / Male - 4 Joinpoints
1990-1996 APC = 3.20*
1996-1999 APC = 0.10
1999-2006 APC = -0.81*
2006-2010 APC = -1.39*
2010-2019 APC = -1.11*
Secondhand smoke / Sindh / Male - 3 Joinpoints
1990-1996 APC = 3.75*
1996-1999 APC = 0.66
1999-2007 APC = -1.14*
2007-2019 APC = -1.59*

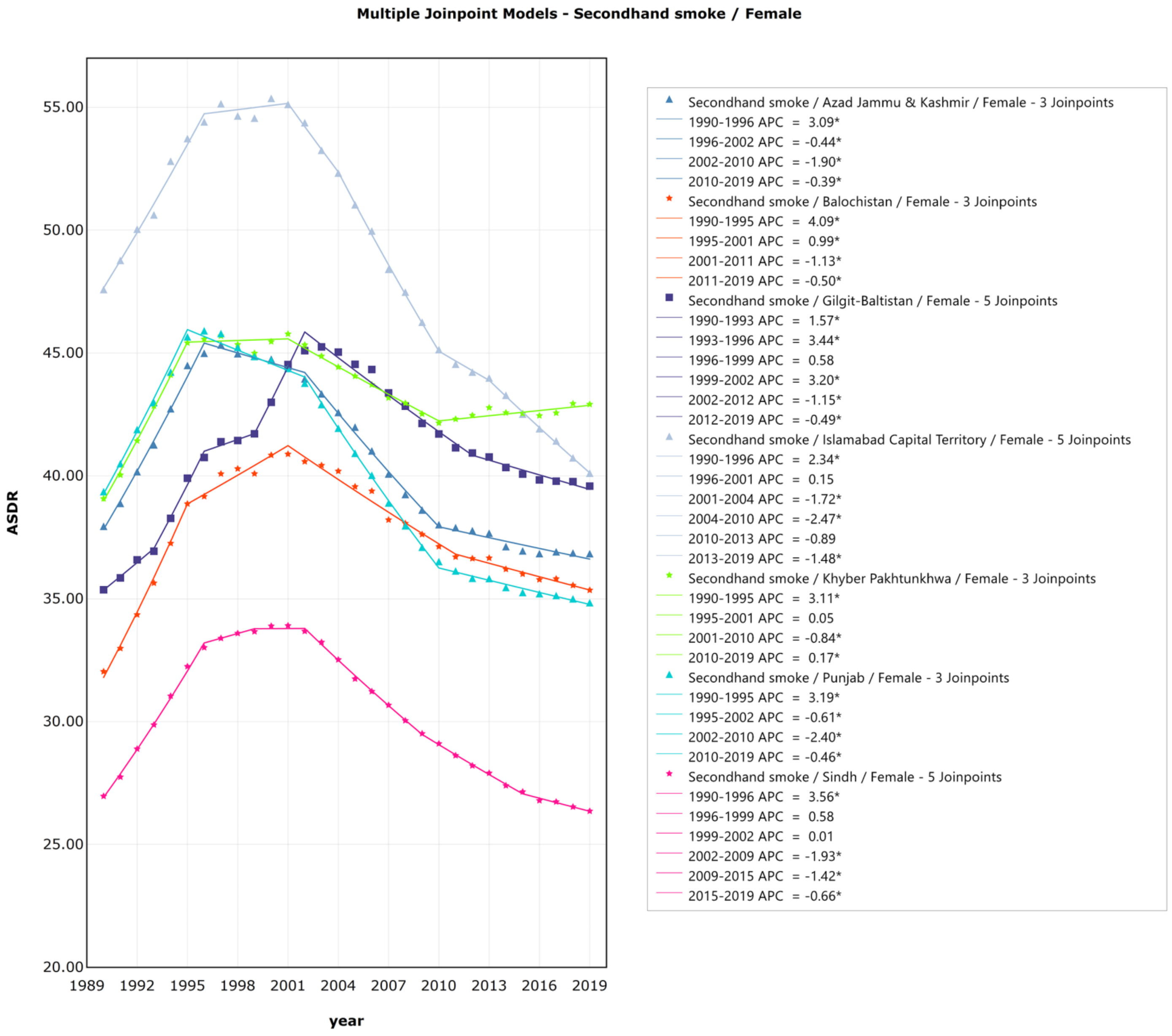

Multiple Joinpoint Models - Secondhand smoke / Female
ASDR
55.00
50.00
45.00
40.00
35.00
30.00
25.00
20.00
1989
1992
1995
1998
2001
2004
2007
2010
2013
2016
2019
year
Secondhand smoke / Azad Jammu & Kashmir / Female - 3 Joinpoints
1990-1996 APC = 3.09*
1996-2002 APC = -0.44*
2002-2010 APC = -1.90*
2010-2019 APC = -0.39*
Secondhand smoke / Balochistan / Female - 3 Joinpoints
1990-1995 APC = 4.09*
1995-2001 APC = 0.99*
2001-2011 APC = -1.13*
2011-2019 APC = -0.50*
Secondhand smoke / Gilgit-Baltistan / Female - 5 Joinpoints
1990-1993 APC = 1.57*
1993-1996 APC = 3.44*
1996-1999 APC = 0.58
1999-2002 APC = 3.20*
2002-2012 APC = -1.15*
2012-2019 APC = -0.49*
Secondhand smoke / Islamabad Capital Territory / Female - 5 Joinpoints
1990-1996 APC = 2.34*
1996-2001 APC = 0.15
2001-2004 APC = -1.72*
2004-2010 APC = -2.47*
2010-2013 APC = -0.89
2013-2019 APC = -1.48*
Secondhand smoke / Khyber Pakhtunkhwa / Female - 3 Joinpoints
1990-1995 APC = 3.11*
1995-2001 APC = 0.05
2001-2010 APC = -0.84*
2010-2019 APC = 0.17*
Secondhand smoke / Punjab / Female - 3 Joinpoints
1990-1995 APC = 3.19*
1995-2002 APC = -0.61*
2002-2010 APC = -2.40*
2010-2019 APC = -0.46*
Secondhand smoke / Sindh / Female - 5 Joinpoints
1990-1996 APC = 3.56*
1996-1999 APC = 0.58
1999-2002 APC = 0.01
2002-2009 APC = -1.93*
2009-2015 APC = -1.42*
2015-2019 APC = -0.66*

Supplementary Figure 7. APC of ASDR in ischemic stroke due to tobacco use, active smoking and second-hand smoke by gender in various regions of Pakistan from 1990 to 2019.

A. APC of ASDR in ischemic stroke due to tobacco use in various regions of Pakistan from 1990 to 2019.

B. APC of ASDR in ischemic stroke caused by tobacco use among men in various regions of Pakistan from 1990 to 2019.

C. APC of ASDR in ischemic stroke caused by tobacco use among women in various regions of Pakistan from 1990 to 2019.

D. APC of ASDR in ischemic stroke due to active smoking in various regions of Pakistan from 1990 to 2019.

E. APC of ASDR in ischemic stroke caused by active smoking among men in various regions of Pakistan from 1990 to 2019.

F. APC of ASDR in ischemic stroke caused by active smoking among women in various regions of Pakistan from 1990 to 2019.

G. APC of ASDR in ischemic stroke due to secondhand smoke in various regions of Pakistan from 1990 to 2019.

H. APC of ASDR in ischemic stroke caused by secondhand smoke among men in various regions of Pakistan from 1990 to 2019.

I. APC of ASDR in ischemic stroke caused by secondhand smoke among women in various regions of Pakistan from 1990 to 2019.

© 2024 Hou S. et al.
